# Targeting casein kinase 1**α**-mediated FADD phosphorylation restores sensitivity in select CDK4/6 inhibitor-resistant breast cancer models

**DOI:** 10.1172/JCI199483

**Published:** 2026-07-28

**Authors:** Sahezeel Awadia, Elizabeth K. Ziemke, Nicole M. Curnutt, Emily Kirk, Julianne Thomas, Anna Zimmerman, Maya J. Mileski, Sundaresh Ram, Reine Abou Zeidane, Craig Galban, Christina M. Woo, Corey W. Speers, Judith Sebolt-Leopold, Alnawaz Rehemtulla

**Affiliations:** 1 Department of Radiation Oncology and; 2 Department of Radiology, University of Michigan Medical School, Ann Arbor, Michigan, USA.; 3 Department of Chemistry and Chemical Biology, Harvard University, Cambridge, Massachusetts, USA.; 4 Department of Radiology and Imaging Sciences, Department of Biomedical Engineering, Emory University and Georgia Institute of Technology, Atlanta, Georgia, USA.; 5 Department of Radiation Oncology, University Hospitals Cleveland Medical Center and Case Western Reserve University, Cleveland, Ohio, USA.; 6 Department of Radiation Oncology, University of Alabama at Birmingham, Birmingham, Alabama, USA.

**Keywords:** Cell biology, Oncology, Breast cancer, Cell cycle, Therapeutics

## Abstract

Resistance to CDK4/6 inhibitors (CDK4/6i) combined with endocrine therapy presents a major barrier to improving outcomes in ER^+^ breast cancer. We identified FADD phosphorylation at Ser194 (phospho-FADD) as a mediator of CDK4/6i resistance in the models examined. Phospho-FADD acted as a pseudosubstrate inhibitor of the APC/C-Cdh1 complex, promoting G1/S transition and bypassing the canonical CDK4/6-Rb-E2F pathway. This CDK4/6-independent pathway was associated with PI3K hyperactivation. Clinical relevance of this bypass pathway was supported by increased phospho-FADD and pAKT in 73% of paired patient biopsies at posttreatment recurrence, while the remaining cases exhibited high baseline phospho-FADD and pAKT with intrinsic nonresponse. Inhibition of FADD phosphorylation with the CK1α degrader DEG-77, or PI3K inhibition, restored CDK4/6i sensitivity in resistant cells. In CDK4/6i-refractory xenografts, CK1α degradation combined with CDK4/6i resulted in profound tumor regressions and increased progression-free survival compared with either single agent, including complete tumor regressions in 92% of tumors. These findings support CK1α-mediated FADD phosphorylation as a targetable resistance mechanism in a subset of CDK4/6i-resistant ER^+^/HER2^–^ breast cancer.

## Abstract 2

Blocking FADD phosphorylation restored CDK4/6 inhibitor sensitivity and improved tumor control in resistant ER+ breast cancer models

## Graphical abstract

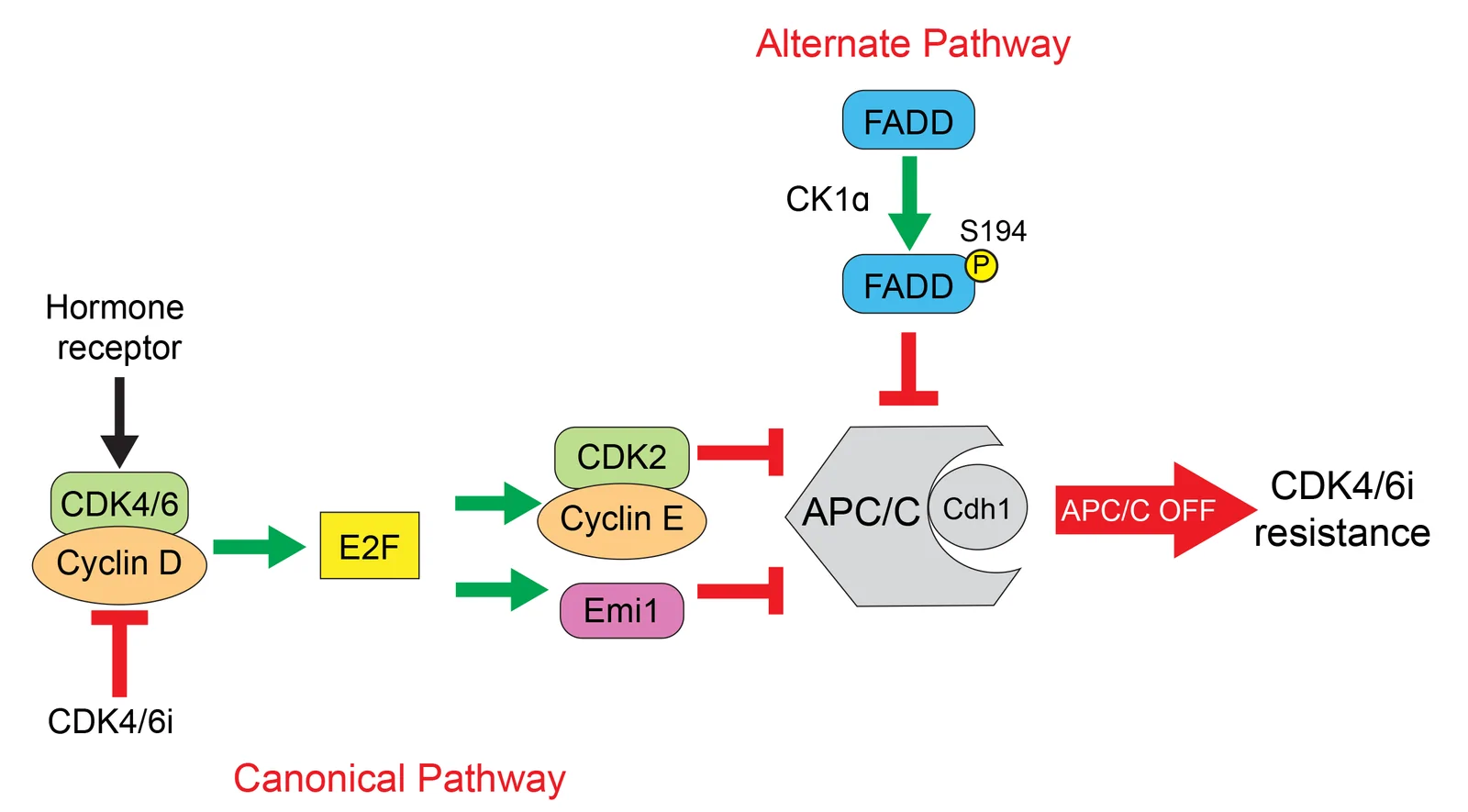

## Introduction

**Figure 1:**
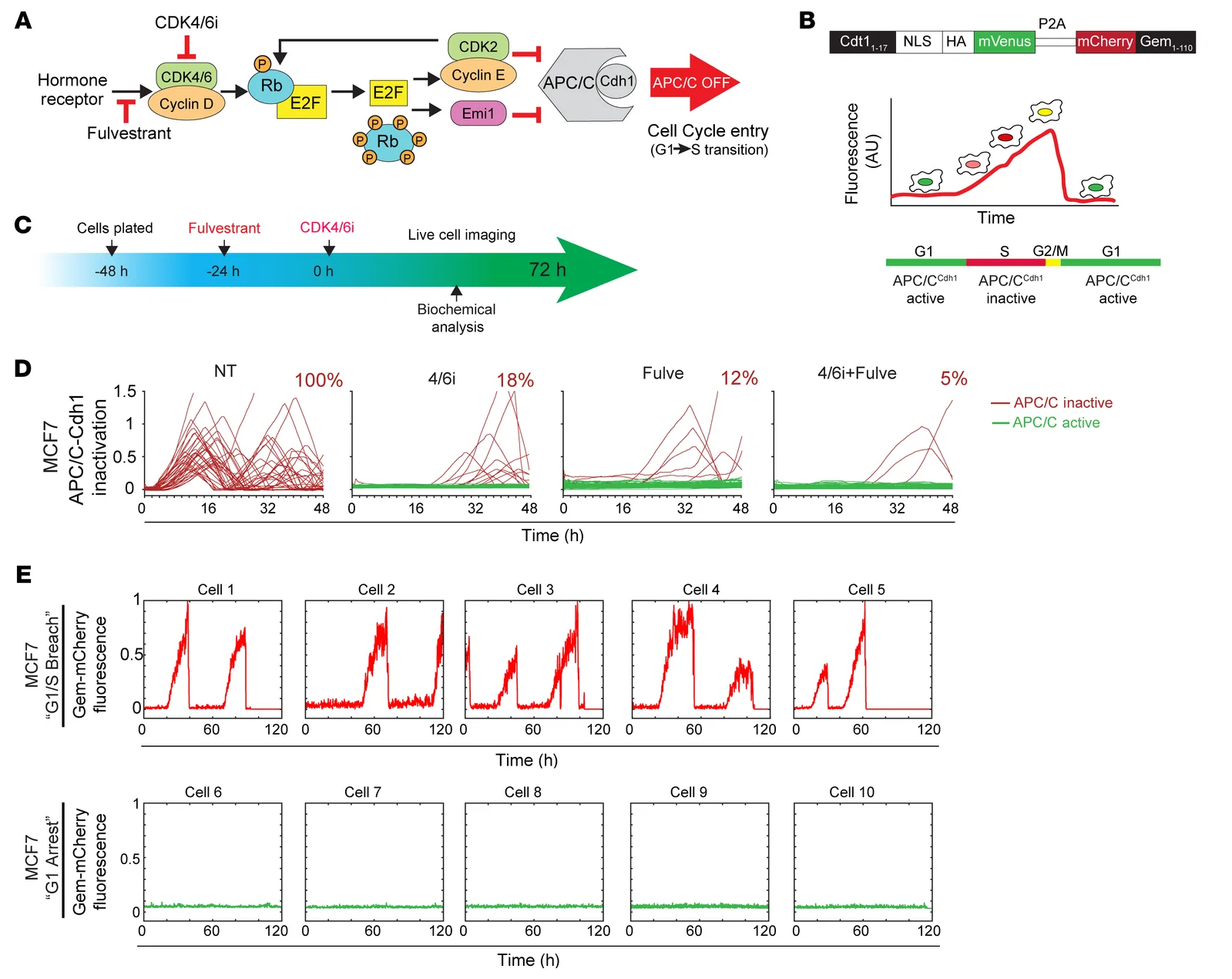
Persistent APC/C-Cdh1 inactivation and G1 to S transition is observed in a subpopulation of cells despite CDK4/6 inhibition. (**A**) Schematic of the canonical Cyclin D-CDK4/6-Rb-E2F pathway for G1/S transition and canonical pathway inhibitors. (**B**) Schematic of the PIP-FUCCI live-cell reporter for APC/C-Cdh1 (mCherry-Geminin) and CRL4-Cdt2 (mVenus-PIP) activity. (**C**) Experimental outline for live-cell imaging of MCF7 cells. (**D**) Representative single-cell traces (mCherry intensity) from MCF7-P cells under indicated treatments (NT, 4/6i, Fulve, 4/6i+Fulve). (**E**) Long-term (120 hours) single-cell traces of mCherry intensity from MCF7 cells treated with CDK4/6i, showing cells that continue cycling (top) or arrest in G1 (bottom). Drug concentrations: 4/6i (ribociclib), 200 nM; Fulvestrant, 100 nM. Data from ≥ 3 biological replicates are shown.

Uncontrolled cell proliferation, a hallmark of cancer, occurs when cells abnormally progress from the G0/G1 phase to the S phase of the cell cycle (1). The E2F family of transcription factors promotes the expression of genes needed for S-phase, including those involved in DNA replication. E2F is kept in check by the nonphosphorylated Retinoblastoma (Rb) protein in resting cells (2–5). The transition from G1 to S phase is primarily regulated by CDK4 and CDK6 kinases, which, in partnership with D-type cyclins, phosphorylate Rb. Rb phosphorylation releases E2F, allowing it to transcribe genes necessary for entry into S phase, as depicted in Figure 1A (6–10).

Small-molecule inhibitors targeting CDK4/6 kinases (CDK4/6i) namely, palbociclib, ribociclib, and abemaciclib are approved for ER^+^/HER2^–^ breast cancer in combination with endocrine therapy (ET), such as aromatase inhibitors (letrozole) or a selective estrogen receptor degrader (SERD) (fulvestrant) (11–13). Despite their effectiveness, over 30% of patients experience disease recurrence within 2 years (14, 15). This resistance is often driven by alterations in the cell cycle and upstream signaling pathways. Well-characterized mechanisms include loss of RB1, amplification of CCNE1 (cyclin E) or CDK6, activation of cyclin E-CDK2, and alterations in upstream signaling pathways such as FGFR, RAS, and AKT (3, 16–23). Notably, the phosphoinositide 3-kinase (PI3K) pathway has often been attributed as a mechanism of resistance to CDK4/6 inhibitors (24–27). These studies culminated in the approval of the PI3K inhibitor inavolisib combined with palbociclib and fulvestrant for PIK3CA-mutant, HR^+^/HER2^–^ locally advanced or metastatic breast cancer that progresses despite CDK4/6i and endocrine therapy (28). However, an understanding of how PI3K signaling effectuates a G1 to S transition in the absence of CDK4/6 activity is lacking. Since PI3K inhibition is commonly accompanied by adverse events in patients, such as hyperglycemia, diarrhea, stomatitis, and rash, elucidation of molecular events that bypass the canonical CDK4/6-dependent pathway should reveal additional therapeutic targets for improved combination strategies for CDK4/6-refractory patients.

The anaphase-promoting complex or cyclosome (APC/C-Cdh1), a large E3 ubiquitin ligase, plays a crucial role in maintaining a unidirectional G1 to S transition by targeting key cell cycle regulators for destruction via ubiquitin-mediated proteasomal destruction (29–31). During mitotic exit and the G1 phase, Cdh1 serves as the substrate-binding subunit of APC/C, preventing inappropriate entry into S phase and preserving genomic integrity (32). Inactivation of APC/C-Cdh1 is essential for the G1/S transition and involves E2F-mediated transcription of Emi1, a pseudosubstrate inhibitor of APC/C-Cdh1, and Cyclin E, which activates the CDK2 kinase (33–35). Phosphorylation of Cdh1 by CyclinE/CDK2 leads to APC/C-Cdh1 inactivation, allowing accumulation of proteins necessary for S phase entry and DNA replication (36–41).

We previously reported that the proapoptotic adaptor protein FADD (Fas-associated protein with death domain) functions as an APC/C-Cdh1 inhibitor upon its phosphorylation at Ser194 by casein kinase 1α (CK1α) (42). In the current study, we found that ER^+^/HER2^–^ breast cancer models and patient-derived biopsies from patients with CDK4/6 inhibitor-resistant disease exhibited enhanced phosphorylation of FADD (phospho-FADD) and activation of PI3K signaling. We further found that activation of PI3K was required for increased FADD phosphorylation in CDK4/6i-resistant cells, and that inhibition of PI3K signaling or therapeutic targeting of CK1α reduced FADD phosphorylation and reestablished the G1/S checkpoint in cells resistant to CDK4/6 inhibition. Combining CDK4/6 inhibitors with the CK1α-targeted agent DEG-77 induced cell cycle exit and senescence in cultured cells and mouse xenograft models. These mechanistic insights into alternate mechanisms for cell cycle entry and CDK4/6i resistance support a therapeutic strategy for a subset of patients with CDK4/6i-resistant ER^+^/HER2^–^ breast cancer.

## Results

### A breach in the G1/S checkpoint contributes to CDK4/6i resistance.

To delineate regulatory circuits for the G1 to S cell cycle transition and their contribution to CDK4/6i resistance (43–46), we embarked on quantitative studies with high temporal resolution at the single-cell level using time-lapse imaging of live cycling cells using the PIP-FUCCI cell cycle reporter (47). This reporter consists of a Cdt1 (PIP)-degron fused to an mVenus fluorescent tag and a Geminin degron fused to a mCherry fluorescent tag. Cdt1-mVenus is stable and accumulates during G1, while Geminin-mCherry is degraded by the APC/C-Cdh1 E3 ubiquitin ligase from anaphase until the end of G1. Therefore, this reporter allows for quantitative monitoring of APC/C-Cdh1 inactivation using mCherry accumulation as a surrogate for the G1 to S transition (Figure 1B). To quantitatively assess how standard-of-care therapies, including CDK4/6 inhibitors (CDK4/6i) and endocrine agents like fulvestrant, impact the G1/S checkpoint in ER^+^ breast cancer models, we monitored APC/C-Cdh1 dynamics (Figure 1C). The PIP-FUCCI reporter was stably expressed in 2 estrogen-dependent ER^+^ breast cancer cell lines (MCF7 and T47D). Rapid degradation of Geminin-mCherry was observed in both untreated cell lines upon mitosis, which persisted throughout G1, indicative of active APC/C-Cdh1. Upon S-phase entry, accumulation of Geminin-mCherry was observed in untreated cells, indicative of APC/C-Cdh1 inactivation at approximately 6–8 hours after mitosis (Figure 1D, NT). In the presence of CDK4/6i (ribociclib), a majority of MCF7 cells experienced an arrest in G1, as observed by a lack of Geminin-mCherry accumulation (i.e., APC/C-Cdh1 remained active greater than 48 hours after mitosis). Interestingly, a subset of MCF7 cells and T47D cells experienced APC/C-Cdh1 inactivation despite the inhibition of CDK4/6, as indicated by a delayed but robust accumulation of mCherry-Geminin within 16–24 hours after mitosis (Figure 1D, 4/6i for MCF7; Supplemental Figure 1A, 4/6i for T47D; supplemental material available online with this article; https://doi.org/10.1172/JCI199483DS1). A similar phenotype was observed in response to fulvestrant (SERD), wherein the majority of MCF7 and T47D cells arrested in G1, while a subset of cells (12% for MCF7 and 33% for T47D) demonstrated a delayed but continued Geminin-mCherry accumulation indicative of APC/C-Cdh1 inactivation (Figure 1D, Fulve for MCF7; Supplemental Figure 1A, Fulve for T47D). Interestingly, a combination of CDK4/6i and fulvestrant resulted in a robust G1 arrest compared with either treatment alone (Figure 1D, 4/6i+Fulve for MCF7; Supplemental Figure 1A, 4/6i+Fulve for T47D).

**Figure 2:**
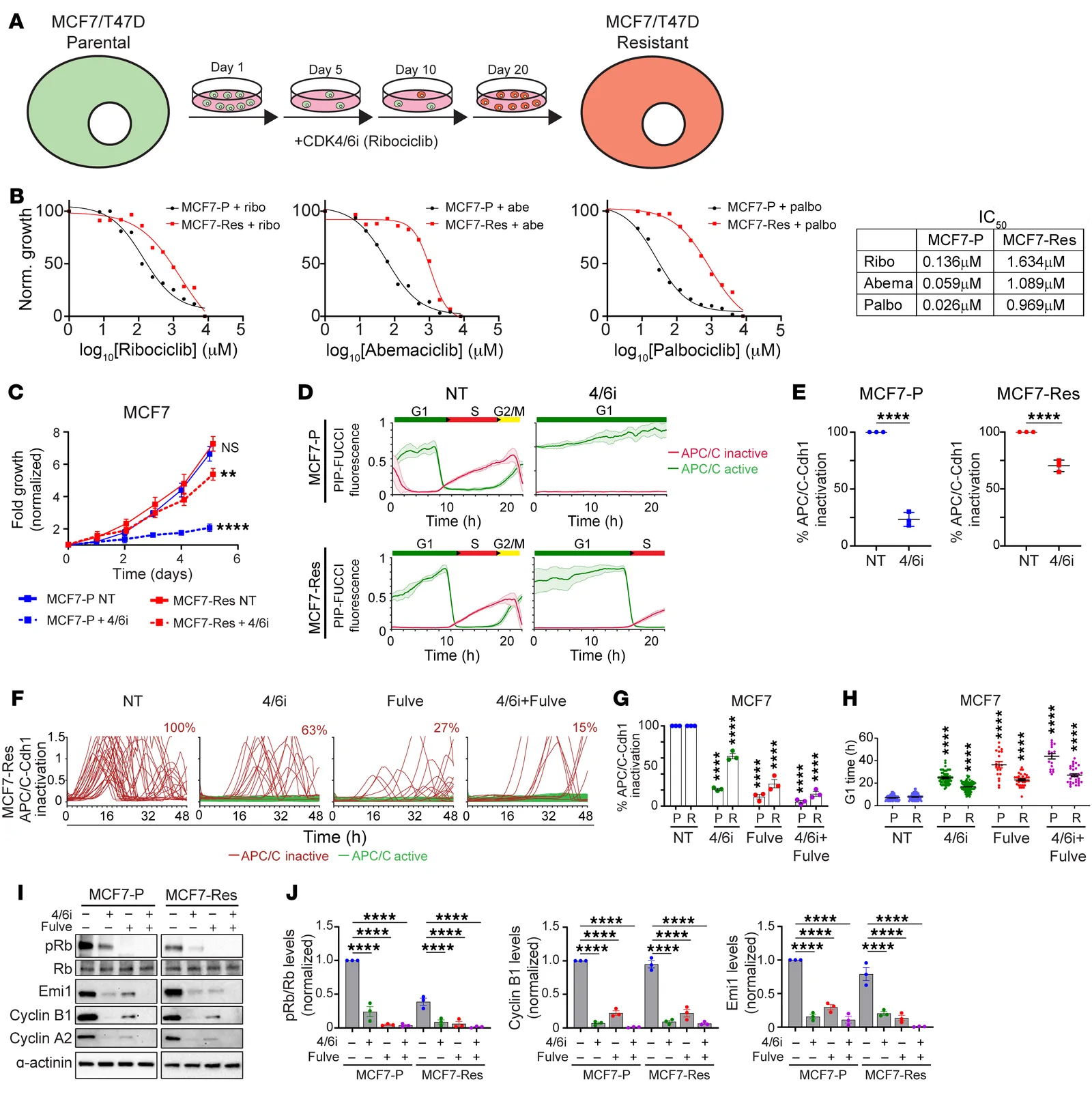
CDK4/6 inhibitor–resistant cells bypass the CDK4/6-Rb-E2F axis via a breached G1/S checkpoint. (**A**) Schematic of the derivation of ribociclib-resistant MCF7-Res and T47D-Res cell lines. (**B**) Dose-response curves and IC_50_ values for CDK4/6i in parental (P) and resistant (Res) MCF7 cells. (**C**) Growth kinetics of MCF7-P and MCF7-Res cells treated with vehicle or CDK4/6i (200 nM). Data are mean ± SEM (*n* = 3). (**D**) Mean live-cell imaging traces (mCherry and mVenus intensity) from MCF7-P and MCF7-Res cells, untreated or treated with CDK4/6i. (**E**) Percentage of MCF7-P and MCF7-Res cells that undergo G1/S transition (APC/C-Cdh1 inactivation). (**F**) Representative mCherry intensity traces from MCF7-Res cells under indicated treatments. (**G**) Quantification of the percentage of MCF7-P and MCF7-Res cells undergoing APC/C-Cdh1 inactivation under treatments in Figure 1D and Figure 2F. (**H**) G1 residence time (hours to mCherry accumulation) for cells in Figure 1D and Figure 2F. (**I**) Immunoblot analysis of phosphorylated Rb (pRb) and total Rb (O/N); E2F targets (Cyclin A2, B1, Emi1) in MCF7-P and MCF7-Res cells after 48 hours treatment. (**J**) Quantification of immunoblots from panel **I** for pRb/tRb, Cyclin B1, and Emi1. Data normalized to NT control. Drug concentrations: 4/6i (ribociclib), 200 nM; Fulvestrant, 100 nM. For quantitative comparisons in **C**, **E**, **G**, **H**, and **J**, statistical significance was determined by 2-way ANOVA with Šídák’s multiple-comparisons correction. Data are mean ± SEM from ≥ 3 biological replicates. *P < 0.05; **P < 0.01; ***P < 0.001; ****P < 0.0001.

We hypothesized that the small population of cells exhibiting APC/C-Cdh1 inactivation and G1/S transition despite CDK4/6 inhibition, may be inherently resistant to CDK4/6i. To evaluate this, we monitored cycles of APC/C-Cdh1 inactivation and activation (upon metaphase/anaphase transition) in individual MCF7 cells over a 120 hours time period. Cells that demonstrated a breached G1/S checkpoint in the presence of CDK4/6i also exhibited this phenotype in subsequent cell cycles (Figure 1E). Conversely, cells that demonstrated an intact G1/S checkpoint remained arrested at G1 in the presence of CDK4/6i over the entire 120 hour imaging period (Figure 1E). To elucidate the mechanistic underpinnings for the observed refractory phenotype to CDK4/6 inhibition, we enriched MCF7 and T47D cultures for this phenotype through selection in the presence of CDK4/6i (ribociclib) for 4 weeks (Figure 2A). The derived cell lines, i.e. ribociclib-resistant MCF7 or T47D cells (hereafter referred to as MCF7-Res or T47D-Res) demonstrated cross resistance to palbociclib and abemaciclib, as evidenced by a 10-fold increase in IC_50_ values compared with the parental cultures (hereafter referred to as MCF7-P or T47D-P cells) (Figure 2B). MCF7-Res cells displayed growth characteristics analogous to MCF7-P cells in the absence of treatment but exhibited minimal growth delay in the presence of CDK4/6i (Figure 2C). Live cell imaging studies revealed that treatment with a CDK4/6i led the majority of MCF7-P cells to display a G1 residence time of greater than 24 hours compared with 6–8 hours seen in untreated MCF7-P cells, consistent with an absence of Geminin-mCherry accumulation after mitosis, indicative of active APC/C-Cdh1 for the entire imaging period (Figure 2, D and E). In MCF7-Res cells, a breach in the G1/S checkpoint in the presence of CDK4/6i was observed (Figure 2F). Treatment with fulvestrant also revealed a breach in the G1/S checkpoint, as fulvestrant regulates the cell cycle upstream of CDK4/6. A larger fraction of MCF7-Res cells exhibited APC/C-Cdh1 inactivation compared with MCF7-P cells with a shorter G1 residence time (Figure 2, F and G). This finding indicated that the breach in the G1/S checkpoint in cells selected for ribociclib resistance was also evident in response to fulvestrant. Interestingly, the combination of fulvestrant and CDK4/6i in MCF7-Res cells revealed an additive effect on the percentage of cells exhibiting APC/C-Cdh1 inactivation (Figure 2, F and G) as well as the G1 residence time (Figure 2, F and H). Comparable results were obtained in T47D-Res cells treated with fulvestrant alone or in combination with CDK4/6i (Supplemental Figure 1, A–C), further suggesting that the resistant phenotype likely resulted from signaling alterations downstream rather than upstream of CDK4/6-Rb-E2F.

**Figure 3:**
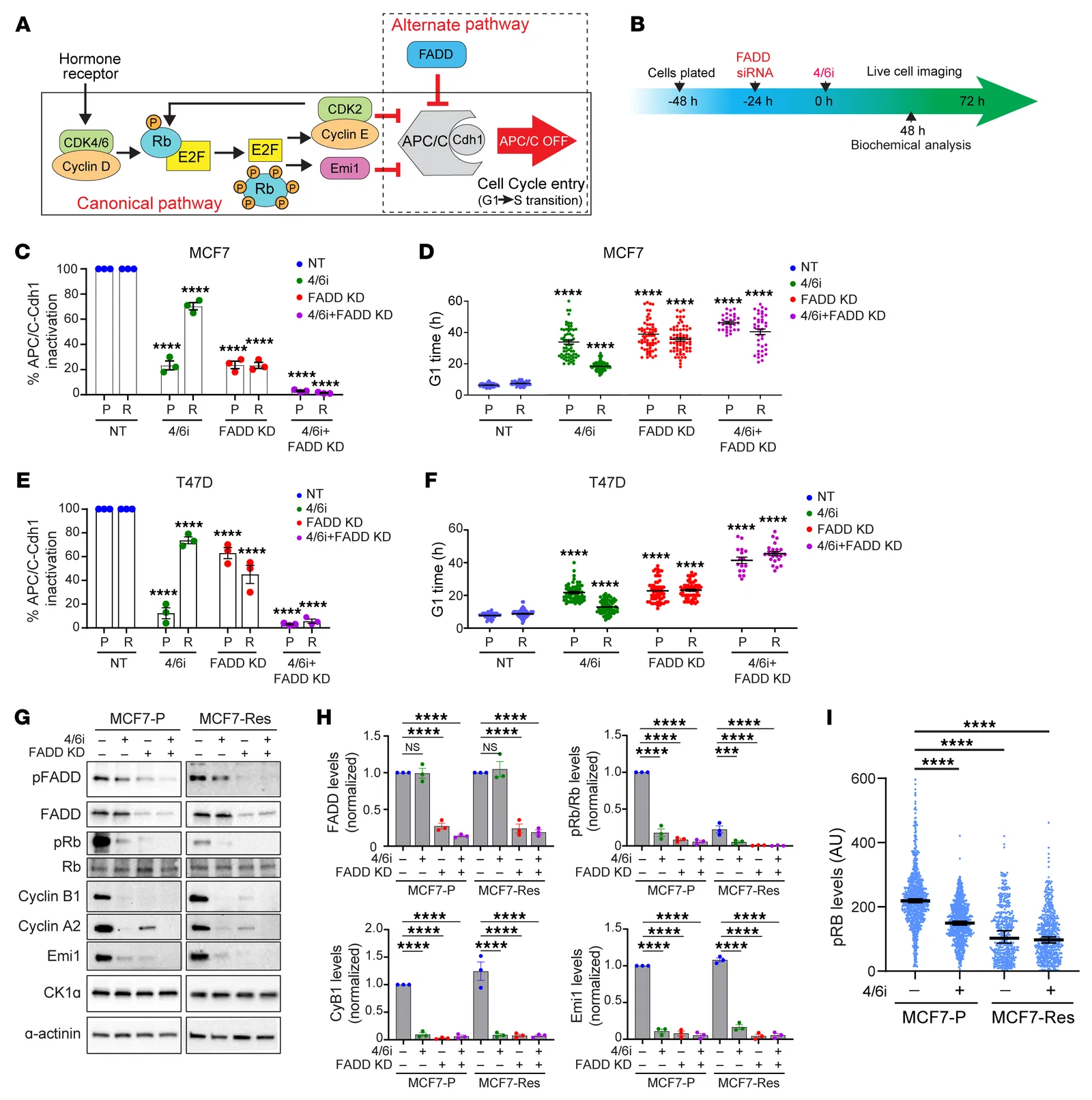
FADD-mediated APC/C-Cdh1 inactivation provides a CDK4/6-independent mechanism for cell cycle entry contributing to CDK4/6i resistance. (**A**) Schematic model of FADD as an alternate APC/C-Cdh1 inhibitor, bypassing the canonical cell cycle pathway. (**B**) Experimental outline for siRNA-mediated FADD knockdown (KD) and live-cell imaging. (**C**) Percentage of MCF7-P and MCF7-Res cells undergoing APC/C-Cdh1 inactivation after FADD KD and/or CDK4/6i treatment. (**D**) G1 residence time for conditions in **C**. (**E**) Percentage of T47D-P and T47D-Res cells undergoing APC/C-Cdh1 inactivation after FADD KD and/or CDK4/6i treatment. (**F**) G1 residence time for conditions in **E**. (**G**) Immunoblot analysis of the indicated proteins in MCF7-P and MCF7-Res cells after FADD KD and/or CDK4/6i treatment. pRb/Rb: O/N; downstream markers: 48 hours. (**H**) Quantification of immunoblots from **G**. *n* ≥ 3. (**I**) Representative immunofluorescence images and quantification of pRb (Ser807/811) in MCF7-P and MCF7-Res cells treated with CDK4/6i. Drug concentration: 4/6i, 200 nM. For **C**–**F** and **H**, 2-way ANOVA with Šídák’s correction was used; for **I**, 2-tailed unpaired *t* test with Welch’s correction was used. Data are mean ± SEM from ≥ 3 biological replicates. *P < 0.05; **P < 0.01; ***P < 0.001; ****P < 0.0001.

Biochemical validation was performed at early time points aligned with the PIP-FUCCI imaging window, when MCF7-Res cells underwent APC/C-Cdh1 inactivation despite CDK4/6 inhibition. pRb and total Rb were measured from matched lysates. In response to CDK4/6 inhibition, as well as fulvestrant, MCF7-P and MCF7-Res cells demonstrated a decrease in pRb/Rb levels as well as a lack of expression of cyclin A2, cyclin B1 and Emi1 (Figure 2, I and J, and Supplemental Figure 1D). Notably, total Rb protein levels remained constant (Figure 2I), indicating the reduction was in phosphorylated state of Rb, not protein abundance. The absence of E2F target genes (Cyclin A2, Cyclin B1, and Emi1) and Rb phosphorylation in the presence of CDK4/6i or fulvestrant in MCF7-Res cells indicated that the canonical (CDK4/6-Rb-E2F) cell cycle entry pathway was intact and not a contributor to the CDK4/6i-resistant phenotype. Inactivation of APC/C-Cdh1 (Geminin-mCherry accumulation; Figure 2, F and G) in MCF7-Res cells treated with CDK4/6i or fulvestrant, despite the absence of Emi1 (an E2F target), suggested that the observed resistance to CDK4/6i may be attributed to an Rb-E2F-independent bypass mechanism for entry into S phase (Figure 3A).

### FADD mediates APC/C-Cdh1 inactivation and provides an alternate path for G1 to S transition.

Evidence that entry into the cell cycle can occur in the absence of CDK4/6 activity has previously been suggested as a basis for CDK4/6i-resistance (3, 48). However, a molecular basis for alternate (CDK4/6-Rb-E2F independent) mechanisms for APC/C-Cdh1 inactivation is lacking. Our and other studies (42, 49–52) have revealed that CK1α-mediated phosphorylation at Ser194 transforms FADD from an adaptor for death receptor–induced apoptotic cell death into a regulator of the cell cycle. We have subsequently demonstrated that phospho-FADD functions as a pseudosubstrate inhibitor of the APC/C-Cdh1 (summarized in Figure 3A) (42). We therefore hypothesized that FADD may provide an alternate, CDK4/6-independent mechanism for APC/C-Cdh1 inactivation and cell cycle entry, thereby contributing to CDK4/6-resistance in ER^+^/HER2^–^ breast cancer models. To test this hypothesis, we performed siRNA-mediated knockdown of FADD in MCF7-P and MCF7-Res cells harboring the PIP-FUCCI reporter (outlined in Figure 3B). Compared with untreated cells (NT), FADD depletion (FADD KD) resulted in a defect in APC/C-Cdh1 inactivation as evidenced by Geminin-mCherry accumulation in 25% of cells which was delayed to greater than 30 hours, compared with 8 hours in 100% of untreated cells (Supplemental Figure 3A and quantified in Figure 3, C and D). Importantly, combined inhibition of the canonical CDK4/6 pathway using ribociclib and the FADD-dependent alternate pathway using siRNA (4/6i+FADD KD) resulted in a near-complete loss of APC/C-Cdh1 inactivation for the entire imaging period in both MCF7-P and MCF7-Res cells (Figure 3, C and D, individual cell traces in Supplemental Figure 2A). To confirm the direct role of FADD in G1 to S transition, we performed a rescue experiment in MCF7-Res cells. Following siRNA-mediated knockdown of endogenous FADD using a 3′ UTR–targeted siRNA, reexpression of WT Halo-FADD via doxycycline induction (Supplemental Figure 2, C–G) restored efficient APC/C-Cdh1 inactivation. This was evidenced by a significant increase in the percentage of cells undergoing the G1/S transition and a corresponding decrease in G1 residence time compared with FADD-depleted cells (Supplemental Figure 2, D–F). This conditional rescue confirmed that the observed cell cycle defect is directly attributable to the loss of FADD function. Similar results were also observed in T47D-P and T47D-Res cells, wherein a combination of FADD depletion and CDK4/6 inhibition resulted in 98% of cells failing to inactivate APC/C-Cdh1 and therefore experiencing a G1 arrest (Figure 3, E and F, individual cell traces in Supplemental Figure 2B). These findings demonstrated a role for FADD in efficient inactivation of APC/C-Cdh1 during the G1 to S transition in parental and resistant cells, suggesting that FADD provided an alternate mechanism for cell cycle entry. Most interestingly, inhibition of parental and resistant MCF7 and T47D cells using CDK4/6i (canonical pathway) and FADD depletion (alternate pathway) restored the sensitivity of resistant cells to CDK4/6i.

Immunoblot analysis confirmed efficient knockdown of total FADD and a corresponding reduction in its phosphorylated form (pFADD), alongside decreased expression of the canonical E2F transcriptional targets cyclin A2, cyclin B1, and Emi1. Evaluation of Rb phosphorylation status by immunoblot analysis demonstrated that phosphorylation of Rb (pRb) was highest in untreated MCF7-P cells, wherein a dramatic loss of pRb was observed in response to CDK4/6 inhibition, as expected (Figure 3G). Interestingly, FADD depletion also led to a decrease in pRb levels, suggesting that, in the absence of efficient APC/C-Cdh1 inactivation in FADD-depleted cells, pRb levels fail to accumulate efficiently. When CDK4/6 inhibition was combined with FADD depletion, pRb levels were lower than those observed in response to either treatment. Significantly lower levels of basal Rb phosphorylation was observed in untreated MCF7-Res cells compared with MCF7-P cells (Figure 3G and quantification in Figure 3H and Supplemental Figure 3A), which further suggested that MCF7-Res cells were less dependent on the canonical CDK4/6-Rb-E2F pathway for entry into the cell cycle. Importantly, immunoblot analysis confirmed that total Rb protein levels remained constant in these conditions (Figure 3G, and quantification in Figure 3H), indicating that the observed reductions were specific to the phosphorylation state of Rb, not its abundance. Single-cell IHC for pRb further demonstrated that untreated MCF7-Res cells exhibited lower levels of pRb compared with MCF7-P cells (Figure 3I and Supplemental Figure 3B) suggesting a greater dependence on the alternate FADD-dependent pathway for cell cycle entry rather than the canonical CDK4/6-Rb-E2F pathway in CDK4/6i-resistant cells. CDK4/6i-treatment of MCF7-Res cells resulted in a dramatic loss of pRb levels to an extent that was greater than similarly treated MCF7-P cells, despite the fact that a majority of MCF7-Res cells continued to enter the cell cycle, as evidenced by APC/C-Cdh1 inactivation. In aggregate, these findings provide evidence that FADD’s function as an APC/C-Cdh1 inhibitor promoted a G1 to S cell cycle transition independent of the canonical CDK4/6-Rb-E2F pathway. Depletion of FADD inactivated the alternate FADD-mediated pathway, which, when combined with CDK4/6i, resulted in restoration of the breached G1/S checkpoint in CDK4/6i-resistant MCF7 and T47D cells (Figure 3, C and D, and Supplemental Figure 2B respectively).

### Enhanced FADD phosphorylation and PI3K signaling were associated with CDK4/6i resistance.

**Figure 4:**
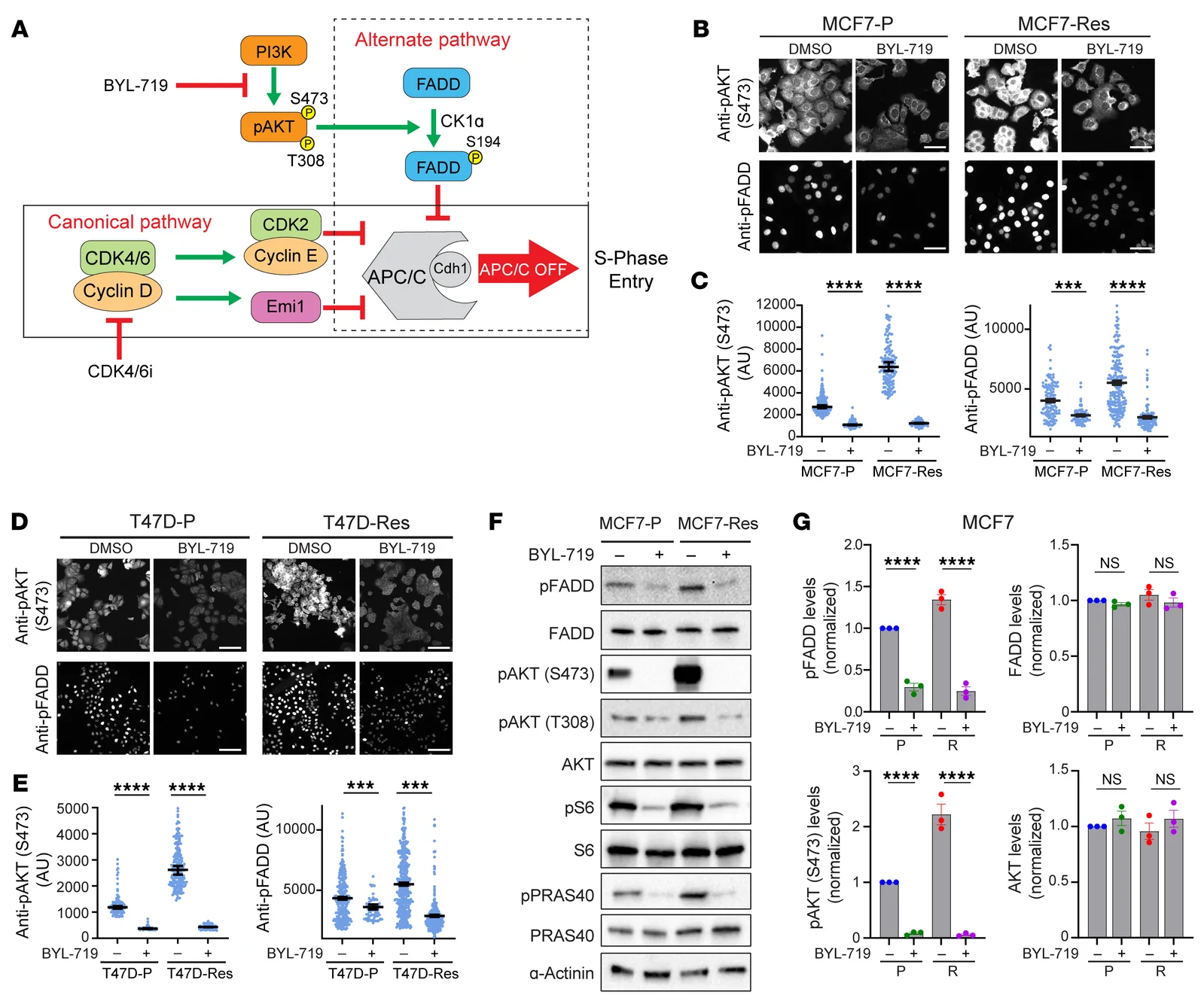
Resistance to CDK4/6 inhibitors is associated with increased PI3K signaling and phosphorylation of FADD. (**A**) Schematic model linking PI3K hyperactivation to CK1α-mediated FADD phosphorylation and APC/C-Cdh1 inactivation in resistance. (**B**) Representative immunofluorescence images of pAKT(S473) and pFADD(S194) in MCF7-P and MCF7-Res cells treated with the PI3K inhibitor BYL-719 (300 nM). (**C**) Scatter plot quantification of single-cell pAKT and pFADD intensity from **B**. Each point represents one cell. (**D**) Representative immunofluorescence images of pAKT and pFADD in T47D-P and T47D-Res cells treated with BYL-719. (**E**) Scatter plot quantification of single-cell pAKT and pFADD intensity from **D**. (**F**) Immunoblot analysis of PI3K/AKT pathway components and phospho-FADD in MCF7-P and MCF7-Res cells treated with BYL-719 for 48 hours. (**G**) Quantification of immunoblots from **F** for pFADD, FADD, pAKT(S473), and total AKT. *n* ≥ 3. Drug concentration: BYL-719, 300 nM. For **C**, **E**, and **G**, 2-way ANOVA with Šídák’s multiple-comparisons correction was performed on biological replicate means. Single-cell values are shown in **C** and **E**. Data are mean ± SEM from ≥ 3 biological replicates. *P < 0.05; **P < 0.01; ***P < 0.001; ****P < 0.0001.

FADD’s function as a pseudosubstrate inhibitor of APC/C-Cdh1 and regulator of the G1 to S cell cycle transition (42) requires its phosphorylation by CK1α at Serine 194, resulting in its translocation to the nucleus (refs. 49, 50, 53, 54; summarized in Figure 4A). We therefore hypothesized that the alternate, FADD-mediated pathway may be enhanced in MCF7-Res cells, thereby contributing to cell cycle entry despite CDK4/6-inhibition. Indeed, MCF7-Res cells exhibited increased levels of nuclear localized total FADD by immunostaining compared with MCF7-P cells (Supplemental Figure 4, A and B). FADD staining was diffused in MCF7-P cells (cytosolic and nuclear), in contrast with MCF7-Res cells wherein FADD was seen predominantly nuclear localized in untreated as well as CDK4/6i-treated cells. We also observed a significant increase in nuclear-localized FADD upon treatment with CDK4/6i in both parental and resistant MCF7 cells (Supplemental Figure 4, A and B). Nuclear localization of FADD was evident in both G1 and S phases of the cell cycle, suggesting that nuclear localization of FADD precedes the G1 to S phase transition in MCF7-Res cells (Supplemental Figure 4B). Additionally, phospho-FADD–specific antibodies (Ser194) confirmed that nuclear-localized FADD is Ser194 phosphorylated (Supplemental Figure 4C) in both G1 phase and S phase cells (Supplemental Figure 4D). Increased nuclear localization of phospho-FADD was also observed in T47D-Res cells compared with T47D-P cells (Supplemental Figure 4, E and F). The observed variation in FADD and phospho-FADD staining intensities reflects heterogeneity within the asynchronous cell population, consistent with the dynamic, cell cycle phase–dependent regulation of FADD and its phosphorylation (42). These findings supported our hypothesis that enhanced FADD phosphorylation and nuclear localization provided an alternate (CDK4/6-Rb-E2F independent) pathway for cell cycle entry and that the breach in the G1/S checkpoint observed in resistant cells may be attributed to enhanced phosphorylation of FADD at Ser194 by CK1α.

Given the frequent dysregulation of the PI3K/AKT/mTOR pathway in breast cancer and its established role in CDK4/6i bypass mechanisms (24–27, 55), we hypothesized that PI3K signaling may be associated with increased FADD phosphorylation to drive resistance (Figure 4A). In agreement with previous findings, we observed enhanced signaling through the PI3K pathway in CDK4/6i refractory MCF7 and T47D cells (Figure 4, B–E). An increase in PI3K signaling was associated with an increase in levels of phospho-FADD. To evaluate if PI3K signaling mediates the increase in FADD phosphorylation, single-cell immunocytochemistry was undertaken, which showed that PI3K inhibition using alpelisib (BYL-719) resulted in a reduction in both pAKT (S473) and phospho-FADD(S194) (Figure 4B, quantified in Figure 4C). Parallel immunofluorescence studies in T47D-P and T47D-Res cells showed analogous reductions in pAKT (S473) and phospho-FADD upon PI3K inhibition (Figure 4F, quantified in Figure 4G). Western blot analysis confirmed that increased phosphorylation at pAKT (S473) and pAKT(T308) correlated with increased phospho-FADD(S194) in resistant MCF7 cells, and that alpelisib treatment simultaneously suppressed AKT phosphorylation levels and phospho-FADD levels without altering total FADD and total AKT levels (Figure 4F and quantification in Figure 4G and Supplemental Figure 4G). Collectively, these data established that PI3K signaling regulates FADD phosphorylation in both parental and CDK4/6i-resistant ER^+^ breast cancer models.

**Figure 5:**
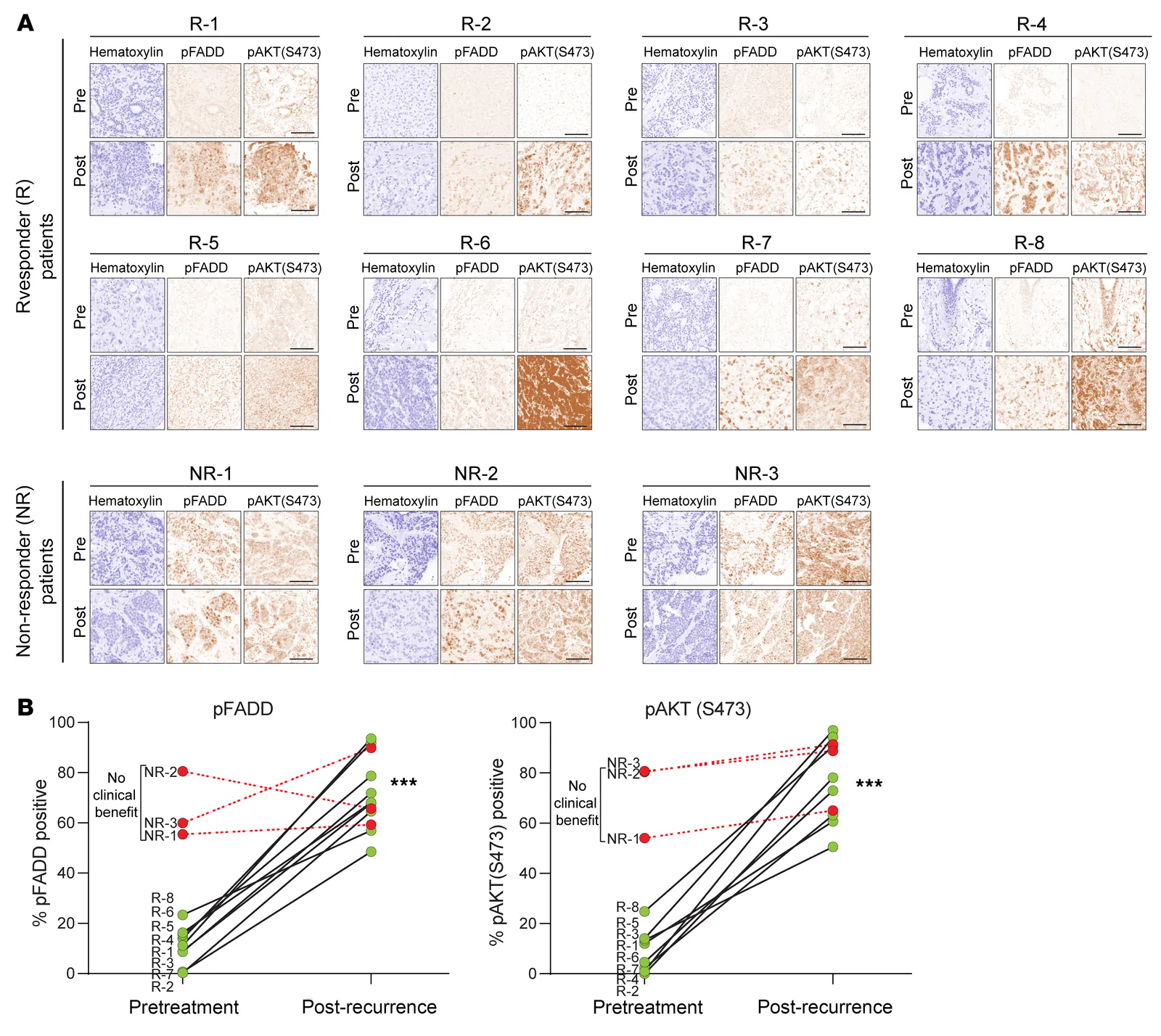
Enhanced PI3K signaling correlates with increased FADD phosphorylation and CDK4/6 inhibitor resistance in patient biopsies. (**A**) Representative immunohistochemistry images (high-power fields) for pFADD(S194) and pAKT(S473) in matched pretreatment (Pre) and postprogression (Post) biopsies from 11 patients. Adjacent hematoxylin-counterstained sections are shown. R1-R8; Responder patients, NR1-NR3; Nonresponder patients. Scale bar: 50 μm. (**B**) Scatter plot of the percentage of pFADD-positive and pAKT-positive tumor cells for each patient’s paired samples (Pre versus Post). Each point represents the mean from greater than 4,000 cells across four ROIs. Green, patients with clinical benefit (R); red, nonresponders (NR). Details of analysis in materials and methods. For **B**, patient-level Pre versus Post values were compared using paired 2-tailed *t* tests with Holm-Šídák’s correction for pFADD and pAKT comparisons. *n* = 11 paired patient samples. Data are mean ± SEM. *P < 0.05; **P < 0.01; ***P < 0.001.

To explore the clinical relevance of the observed enhanced phosphorylation of FADD and activation of the PI3K pathway in CDK4/6i-refractory cells, breast cancer biopsy samples at time of diagnosis and post-recurrence procured at time of metastatic progression while on CDK4/6i therapy were used to investigate phospho-FADD levels and PI3K pathway activation. All available paired biopsies meeting the inclusion criteria were analyzed without exclusion; inclusion required ER^+^/HER2^–^ breast cancer, treatment with a CDK4/6 inhibitor, and availability of matched pretreatment and postprogression biopsy tissue. Compared with their matched pretreatment CDK4/6i-naive samples, posttreatment progression showed increased percentages of phospho-FADD–positive cells in 8 of 11 paired cases, accompanied by increased pAKT levels, consistent with enhanced PI3K signaling (Figure 5A). These images are representative ROIs from the 11 paired cases analyzed at pretreatment (Pre) and postrecurrence (Post) (low magnification entire biopsy in Supplemental Figure 5A). Positivity was quantified as detailed in the Methods. Quantification of phospho-FADD levels over multiple ROIs for each set of individual paired samples demonstrated increased phospho-FADD and pAKT levels in the posttreatment progression samples compared with their paired pretreatment CDK4/6i naive samples (Figure 5B, raw data shown in Supplemental Figure 5B). Strikingly, the 3 patients with tumors showing the highest baseline phospho-FADD and pAKT levels at diagnosis that failed to show further elevation at progression (designated NR-1, NR-2 and NR-3; Figure 5, A and B) derived no clinical benefit from CDK4/6i treatment (abemaciclib or ribociclib nonresponders). All 3 nonresponder patients demonstrated disease progression at their first restaging scan (within 3 months of initiating CDK4/6i therapy), while the remaining 8 patients (R-1 to R-8) showed clinical benefit. Clinical characteristics and treatment history for the 11 ER^+^/HER2^–^ breast cancer cases analyzed in Figure 5 are summarized in Supplemental Table 1. Patients labeled R1–R8 experienced clinical benefit on CDK4/6i (defined as no progression before 6 months), whereas NR1–NR3 represent primary nonresponders (disease progression on CDK 4/6i in less than 6 months) with rapid progression at first restaging. These findings offered a clinical correlation for our laboratory observations that phospho-FADD and pAKT levels were elevated in CDK4/6i refractory cell lines and were consistent with a mechanistic basis for the development of CDK4/6i resistance in patients. Immunohistopathology of these patient samples did not show statistically significant changes in total FADD or total AKT levels (Supplemental Figure 6A and quantification in Supplemental Figure 6B).

**Figure 6:**
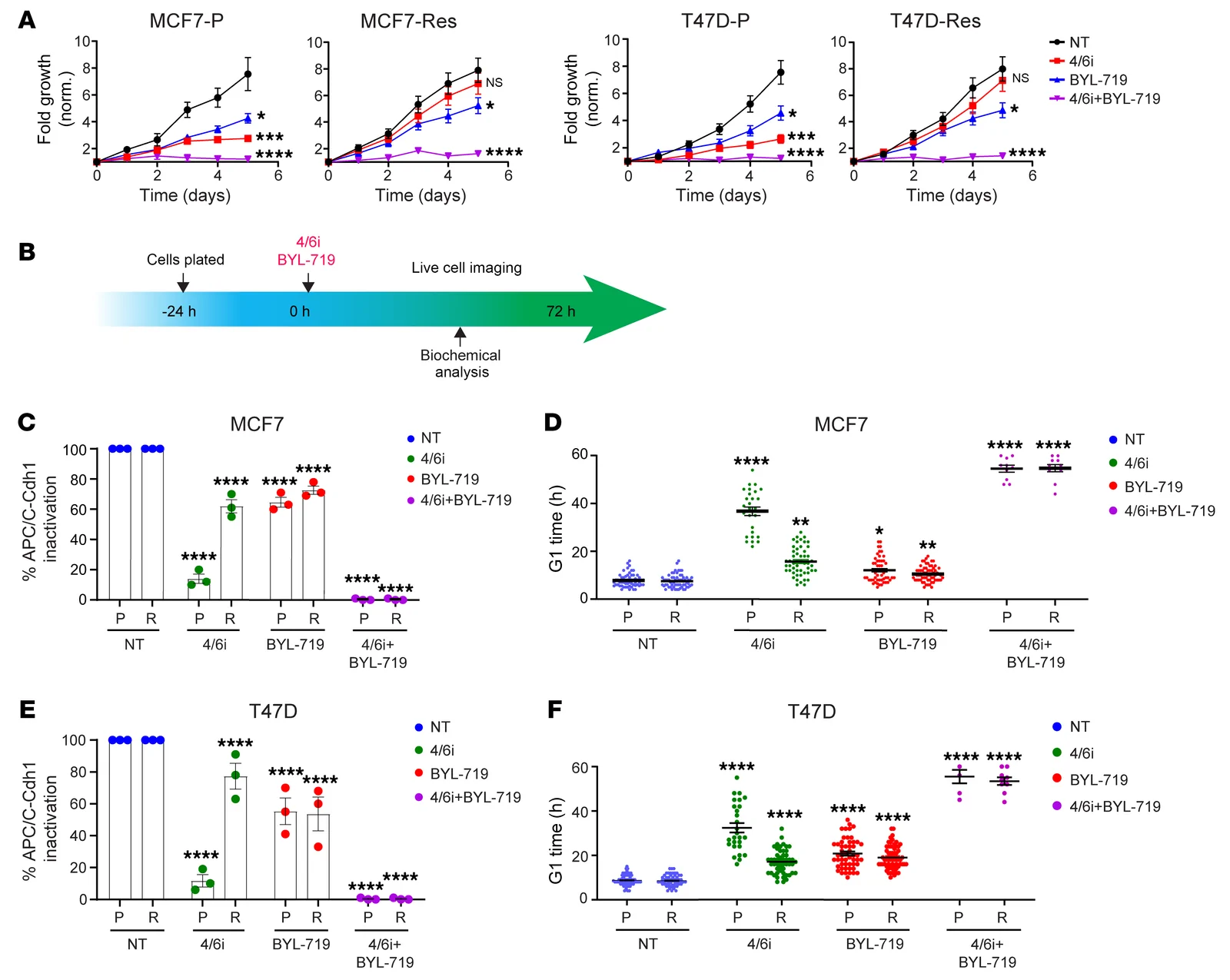
PI3K signaling promotes FADD phosphorylation and a CDK4/6i-resistant phenotype. (**A**) Growth kinetics of MCF7 and T47D parental (P) and resistant (Res) cells treated with CDK4/6i (200 nM), BYL-719 (300 nM), or their combination. (**B**) Experimental outline for live-cell imaging experiments treating MCF7 and T47D cells with BYL-719, CDK4/6i or their combinations. (**C**) Percentage of MCF7-P and MCF7-Res cells undergoing APC/C-Cdh1 inactivation under indicated treatments. (**D**) G1 residence time for conditions in **C**. (**E**) Percentage of T47D-P and T47D-Res cells undergoing APC/C-Cdh1 inactivation under indicated treatments. (**F**) G1 residence time for conditions in **E**. *Drug concentrations: 4/6i, 200 nM; BYL-719, 300 nM. For **A**, 2-way repeated-measures ANOVA was used; for **C**–**F**, 2-way ANOVA was used. All post hoc comparisons used Šídák multiple-comparisons correction. Data are mean ± SEM from ≥ 3 biological replicates. *P < 0.05; **P < 0.01; ***P < 0.001; ****P < 0.0001.

Next, to test if the requirement for PI3K signaling in FADD phosphorylation was mechanistically linked to a CDK4/6i-resistant phenotype, we evaluated cell proliferation rates (Figure 6A) as well as the efficiency of G1 to S transition (Figure 6, B–F, and Supplemental Figure 7) in the presence of the PI3K inhibitor alone or in combination with CDK4/6i. These findings showed that the inhibition of FADD phosphorylation (alternate pathway for G1 to S) using a PI3K inhibitor alone or in combination with CDK4/6i impacted the G1 to S transition. BYL-719 as a single agent had a modest effect on cell growth (Figure 6A) and efficiency of APC/C-Cdh1 inhibition and transition into the cell cycle (Figure 6, C–F, and Supplemental Figure 7) in MCF7 and T47D cells. However, simultaneous inhibition of the PI3K pathway and the CDK4/6-Rb pathway resulted in a robust inhibition of cell growth (Figure 6A) in either cell line, as a result of a G1 arrest in parental and resistant MCF7 cells (Figure 6, C and D; individual traces in Supplemental Figure 7A) as well as T47D cells (Figure 6, E and F; individual traces in Supplemental Figure 7B).

### DEG-77–mediated degradation of CK1α resulted in loss of FADD phosphorylation and restoration of the G1/S checkpoint.

**Figure 7:**
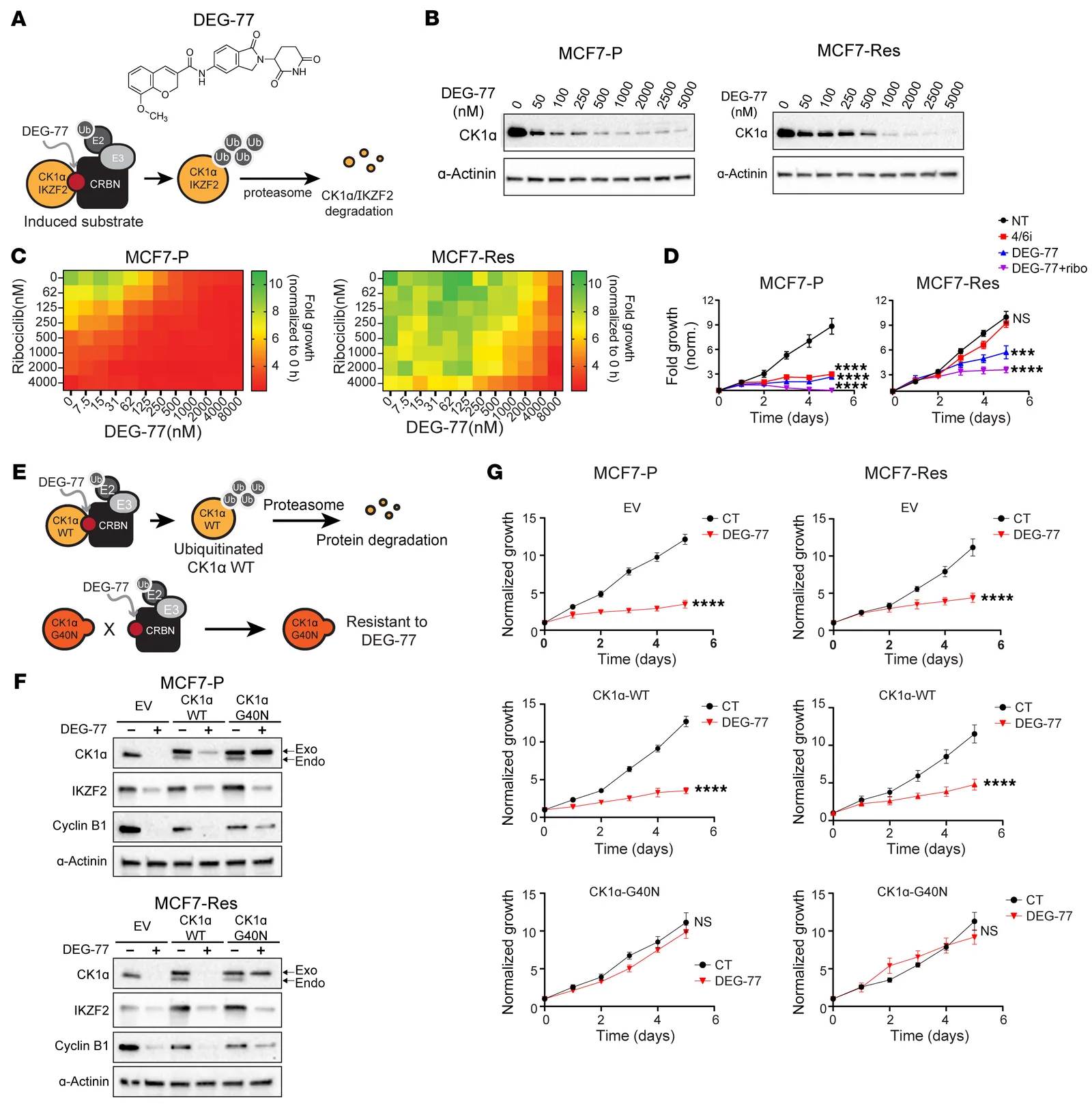
CK1α degradation using DEG-77 inhibits FADD-phosphorylation and alters the cell cycle. (**A**) Chemical structure of DEG-77 and schematic of its mechanism via CRL4-CRBN. (**B**) Immunoblot showing dose-dependent degradation of CK1α in MCF7-P and MCF7-Res cells after 72 hours DEG-77 treatment. (**C**) Dose-response matrix (fold growth at 72 hours) of MCF7-P and MCF7-Res cells treated with DEG-77 and CDK4/6i. (**D**) Proliferation of MCF7-P and MCF7-Res cells treated with DEG-77, CDK4/6i, or combination. (**E**) Schematic of the degradation-resistant CK1α-G40N mutant. (**F**) Immunoblot analysis of CK1α (endogenous/Endo and exogenous/Exo) and IKZF2 in MCF7-P and MCF7-Res cells stably expressing empty vector (EV), CK1α-WT, or CK1α-G40N, treated with DEG-77. (**G**) Proliferation of the cell lines in **F** treated with DEG-77, CDK4/6i, or combination. Drug concentrations: DEG-77, 1 μM (except **B** and **C**); 4/6i, 200 nM (except **C**). For **D** and **G**, 2-way repeated-measures ANOVA with Šídák’s multiple-comparisons correction was used. **C** is descriptive. Data are mean ± SEM from ≥ 3 biological replicates. *P < 0.05; **P < 0.01; ***P < 0.001; ****P < 0.0001**.**

Since adverse events are commonly observed upon PI3K inhibition and can require discontinuation of therapy, we evaluated small molecule degraders of CK1α as an alternate and potentially safe combination therapy to restore the G1/S checkpoint. CK1α degraders are approved for clinical use based on their ability to mediate its ubiquitination and proteasomal degradation via the CRL4-CRBN E3 ubiquitin ligase (Figure 7A) (56–58). Our recently described degrader of CK1α, DEG-77 (57, 58), effectively degraded CK1α at nanomolar concentrations in MCF7-P and MCF7-Res cells (Figure 7B). Next, we investigated the efficacy of dual inhibition of the canonical CDK4/6-Rb-E2F pathway using CDK4/6i and the alternate FADD-dependent pathway using DEG-77. Dose-response matrix analysis (Figure 7C) and cell growth assays (Figure 7D) showed that MCF7-P cells displayed marked growth inhibition at 500 nM of CDK4/6i (vertical axis) compared with untreated control cells. In contrast, MCF7-Res cells displayed minimal inhibition of cell growth in response to CDK4/6i. DEG-77 inhibited the growth of both MCF7-P and MCF7-Res cells as a single agent. Notably, the combination of CDK4/6i with DEG-77 suppressed the growth of both parental and CDK4/6-resistant MCF7 cells more effectively than either compound alone (Figure 7D), suggesting that blockade of the canonical and alternate pathways for APC/C-Cdh1 inactivation and G1/S transition was required for efficient inhibition of cell proliferation.

In addition to CK1α, IKAROS family zinc finger 2 protein (IKZF2) is also a neosubstrate of the CRL4-CRBN E3 ligase in the presence of DEG-77 (Figure 7E) (58). To confirm that the growth inhibitory effect of DEG-77 is mediated by CK1α specifically, we generated stable MCF7 cells expressing either WT CK1α (CK1α-WT) or a G40N mutant of CK1α (CK1α-G40N), which blocks recruitment of CK1α to the CRL4-CRBN complex by DEG-77 (58, 59). Treatment using DEG-77 in both MCF7-P and MCF7-Res cells demonstrated almost complete degradation of endogenous CK1α as well as the exogenously expressed CK1α-WT protein (Figure 7F and quantification in Supplemental Figure 8A). In contrast, stable cells expressing CK1α-G40N failed to show degradation of the mutant protein (Exo), although endogenous CK1α (Endo) was efficiently degraded. Cell growth was inhibited in response to DEG-77 in empty vector (EV) control cells as well as CK1α-WT-expressing cells but was not affected in G40N mutant-expressing cells, in both the MCF7-P and MCF7-Res lines (Figure 7G). PIP-FUCCI live cell imaging studies further confirmed that CK1α-G40N–expressing cells were refractory to DEG-77 treatment (Supplemental Figure 8B), even though IKZF2 was degraded in response to drug treatment (Figure 7F). This further confirmed that the observed inhibition of cell growth and APC/C-Cdh1 inactivation in response to DEG-77 was mediated by degradation of CK1α and independent of IKZF2.

**Figure 8:**
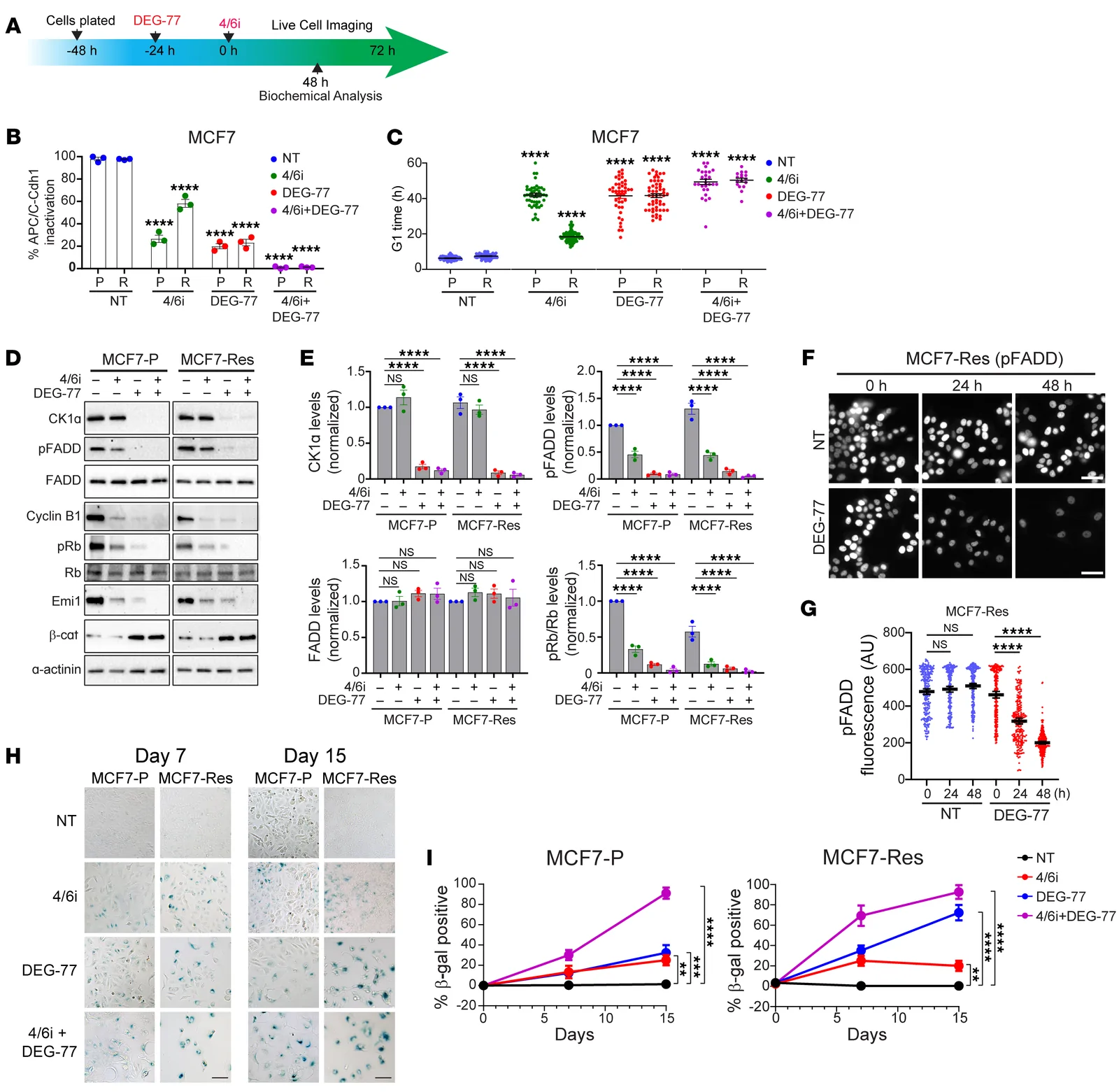
Dual targeting of canonical CDK4/6 and alternate CK1α pathways restores G1/S checkpoint and induces senescence in CDK4/6i-resistant cells. (**A**) Experimental outline for live-cell imaging with DEG-77 and CDK4/6i treatments. (**B**) Percentage of MCF7-P and MCF7-Res cells undergoing APC/C-Cdh1 inactivation under indicated treatments. (**C**) G1 residence time for conditions in **B**. (**D**) Immunoblot analysis of cell cycle and pathway proteins in MCF7-P and MCF7-Res cells treated with DEG-77, CDK4/6i, or combination. pRb and total Rb were analyzed after overnight treatment; downstream markers were analyzed after 48 hours. (**E**) Quantification of immunoblots from **D** for CK1α, pFADD, FADD, and pRb/tRb. (**F**) Representative immunofluorescence images of pFADD(S194) in MCF7-Res cells over time after DEG-77 treatment. Scale bar: 30 μm. (**G**) Quantification of nuclear pFADD intensity from **F** (> 200 cells/condition). (**H**) Representative images of SA-β-gal staining in MCF7-P and MCF7-Res cells at days 7 and 15 after treatment. Scale bar: 30 μm. (**I**) Quantification of SA-β-gal-positive cells from **H**. Data from ≥ 3 replicates. Drug concentrations: DEG-77, 1 μM; 4/6i, 200 nM. For **B**, **C**, **E**, **G**, and **I**, 2-way ANOVA with Šídák’s multiple-comparisons correction was used. Data are mean ± SEM from ≥ 3 biological replicates; single-cell values are shown in **G**. *P < 0.05; **P < 0.01; ***P < 0.001; ****P < 0.0001.

We next evaluated the efficiency of APC/C-Cdh1 inactivation in the presence of DEG-77 and CDK4/6i (Figure 8A). As observed previously in MCF7-P cells, CDK4/6i treatment resulted in 74% of cells experiencing a G1 arrest, while 26% of cells demonstrated a delayed but continued APC/C-Cdh1 inactivation at greater than 20 hours after mitosis (compared with 8 hours for untreated cells). In contrast, CDK4/6 inhibition in MCF7-Res cells resulted in a majority of cells (58%) exhibiting APC/C-Cdh1 inactivation and G1/S transition at greater than 16 hours, confirming a breached G1/S checkpoint, as observed previously (Figure 8B; individual traces in Supplemental Figure 8C). DEG-77 treatment of either MCF7-P or MCF7-Res cells resulted in a majority (80%) of the cells experiencing a G1 arrest (failure to inactivate APC/C-Cdh1), while 20% of cells that did experience APC/C-Cdh1 inactivation required greater than 16 hours to do so compared with 8 hours for untreated cells (Figure 8B and Supplemental Figure 8C). Quantitative analysis of multiple biological replicates showed decreased efficiency for APC/C-Cdh1 inactivation and a shorter G1 residence time in MCF7-Res cells in response to CDK4/6i. However, these parameters were not appreciably different in MCF7-P and MCF7-Res cells in response to DEG-77 as single agent or in combination with CDK4/6i. (Figure 8, B and C). The combination of CDK4/6i and DEG-77 resulted in an almost complete failure to inactivate APC/C-Cdh1 and defective G1/S transition for the entire imaging time in both parental and CDK4/6i-resistant MCF7 cells (Figure 8, B and C; individual traces in Supplemental Figure 8C). This suggested that inactivation of the alternate FADD-dependent pathway for APC/C-Cdh1 inactivation restored sensitivity to CDK4/6i in MCF7-Res cells. The finding that CK1α degradation resulted in a defect in the G1/S transition was independently confirmed using BTX161, an independently developed CK1α degrader (60–62), in MCF7-P and MCF7-Res cells (Supplemental Figure 9, A–E).

Biochemical studies to evaluate the levels of proteins involved in the G1/S transition confirmed that CDK4/6i treatment of MCF7-P and MCF7-Res cells resulted in inhibition of the CDK4/6-Rb-E2F canonical pathway for cell cycle entry, as evidenced by reduced levels of E2F target proteins Emi1 and cyclin B1 (Figure 8, D and E). However, as observed above (Figure 2, I and J), MCF7-Res cells exhibited a lower pRb/Rb ratio (Figure 8, D and E) compared with MCF7-P cells in the absence of treatment, despite a similar rate of cell proliferation (Figure 2C) suggesting a decreased dependence on the CDK4/6-Rb-E2F canonical pathway for cell cycle entry.

The finding that MCF7-Res cells treated with CDK4/6i continued to proliferate and experience delayed but robust APC/C-Cdh1 inactivation in the absence of CDK4/6 activity, as evident by a lack of Rb phosphorylation and E2F target proteins, further suggested that the observed G1/S checkpoint breach in MCF7-Res cells could not be attributed to a difference in efficiency of CDK4/6 inhibition or a defect in Rb function. These biochemical studies also demonstrated that treatment with DEG-77 resulted in degradation of CK1α, leading to reduced FADD phosphorylation in both MCF7-P and MCF7-Res cells without altering total FADD levels (Figure 8D and quantifications in Figure 8E, and Supplemental Figure 10A). Immunocytochemistry also showed reduced nuclear phospho-FADD levels in MCF7-Res cells over a period of 48 hours (Figure 8, F and G), a finding that was confirmed in DEG-77–treated T47D-Res cells (Supplemental Figure 10, B and C). An increase in β-catenin (a CK1α substrate) (63) levels confirmed that DEG-77 treatment resulted in a decrease in CK1α activity in both parental and resistant MCF7 cells (Figure 8D). Importantly, when CDK4/6i and DEG-77 were combined, reduced levels of pRb, Emi1 and Cyclin B1 (markers of inhibition of the canonical cell cycle entry pathway), as well as loss of CK1α activity (decreased FADD phosphorylation), culminated in an almost complete loss of APC/C-Cdh1 inactivation (and therefore G1 phase cell cycle arrest) in both MCF7-P and MCF7-Res cells. Overall, these results indicated that DEG-77 was a potent inhibitor of the FADD-dependent, alternate G1/S transition pathway based on its ability to degrade CK1α and inhibit phosphorylation of FADD.

### A combination of CDK4/6i and DEG-77 induced prolonged cell cycle arrest and senescence.

To investigate if the protracted exit from the cell cycle observed in response to DEG-77 combined with CDK4/6 inhibition resulted in the induction of senescence, we utilized MCF7-P and MCF7-Res cells. Cellular senescence was measured by β-galactosidase (β-gal) positivity at 7 and 15 days after treatment (Figure 8H, quantified in Figure 8I). Untreated cells had undetectable levels of β-gal in either cell line. In MCF7-P and MCF7-Res cells, CDK4/6i induced detectable β-gal positivity to a similar extent. DEG-77 as single agent induced β-gal positivity in MCF7-P cells at a magnitude similar to CDK4/6i treatment. In contrast, DEG-77 treatment in MCF7-Res cells resulted in a greater magnitude of β-gal positivity, especially at day 15. The combination of CDK4/6i and DEG-77 had the most pronounced effect, wherein nearly all cells exhibited β-gal positivity (Figure 8H, quantified in Figure 8I). These findings also suggested that MCF7-Res cells were more dependent on the alternate pathway for cell cycle entry (CK1α-pFADD mediated) since DEG-77 treatment as single agent resulted in an enhanced induction of β-gal positivity (Figure 8I) compared with MCF7-P cells. Next, we asked if epigenetic changes resulted in the expression of senescence-associated secretory phenotype (SASP) in MCF7-P and MCF7-Res cells. Single agent treatment of either CDK4/6i or DEG-77 resulted in a significant increase in the secretion of proteins associated with the SASP in MCF7-P cells but not in MCF7-Res cells (Supplemental Figure 11). A combination of both agents led to a pronounced SASP secretion in MCF7-P cells. In contrast, MCF7-Res cells exhibited a minimal increase in SASP protein secretion with either single agent, and a modest increase in select chemokines (but not growth factors) in response to combination therapy. Muted SASP induction in MCF7-Res cells despite a robust induction of β-gal positivity suggested that the cell cycle exit observed in MCF7-P cells has a distinct senescence phenotype from that observed in MCF7-Res cells.

### DEG-77–mediated arrest in G1 was partially dependent on p53 status.

Next, we evaluated DEG-77 efficacy on T47D-P and T47D-Res cells. Studies on APC/C-Cdh1 inactivation in untreated T47D-P and T47D-Res cells showed that APC/C-Cdh1 inactivation occurred at 8–10 hours after mitosis (Supplemental Figure 12, B–D). CDK4/6i treatment resulted in 91% of T47D-P cells undergoing a G1 arrest, as evidenced by a failure to inactivate APC/C-Cdh1 for the entire imaging time (greater than 48 hours) and a protracted G1 residence time (Supplemental Figure 12D). In contrast, only 32% of T47D-Res cells experienced a G1 arrest in response to CDK4/6i; the remaining population entered the cell cycle after a delay of 10–16 hours (Supplemental Figure 12D). Notably, DEG-77 treatment of T47D-P or T47D-Res cells, had a modest impact on APC/C-Cdh1 inactivation and G1/S transition (Supplemental Figure 12, C and D). A muted response to DEG-77 was also confirmed by the finding that combination of DEG-77 and CDK4/6i resulted in continued inactivation of APC/C-Cdh1 in approximately 50% of T47D-Res cells (Supplemental Figure 12, B and C). Since T47D cells have mutant p53 unlike MCF7 cells, we hypothesized that the muted response to DEG-77 in T47D-P and T47D-Res could be attributed to its p53 status.

In addition to a reduction in FADD phosphorylation, degradation of CK1α has been demonstrated to trigger p53-dependent cell cycle arrest and apoptosis (22, 64–66). p53-mediated induction of p21 has been ascribed to the G1 cell cycle arrest observed in the absence of CK1α activity. p21 inhibits several cyclin-dependent kinases, including CDK1, CDK2, and CDK4/6, thereby contributing to the tumor-suppressive function to the p53-p21 signaling axis (7, 67–70). MDM2, a p53-specific E3 ubiquitin ligase, antagonizes p53 function by its ubiquitination-mediated proteasomal degradation, thereby limiting p53 growth-suppressive functions. CK1α stabilizes MDM2 and phosphorylates MDM4, its homolog. MDM4 and MDM2 form a heterodimer essential for their negative regulation of p53 activity. Loss of CK1a-mediated phosphorylation of MDM4 and CK1a-induced stability of MDM2 destabilizes the p53-MDM4-MDM2 complex, resulting in accumulation of p53 and transcription of p21, leading to cell cycle arrest (refs. 66, 71–73; summarized in Supplemental Figure 12E). To investigate if the differential response to CK1α degradation between MCF7-Res and T47D-Res cells could be attributed to the role of the p53-p21 signaling axis, we treated MCF7-Res and T47D-Res cells with DEG-77 and found that p53 and p21 were upregulated in MCF7-Res cells in the presence of DEG-77–mediated CK1α degradation but not in T47D-Res cells (Supplemental Figure 12F).

To directly test the role of p21, we performed siRNA knockdown of p21 in DEG-77–treated MCF7-Res cells and compared them with T47D-Res cells (Supplemental Figure 13A). Western blot analysis confirmed the loss of p21 protein upon treatment with p21 siRNA in either cell line (Supplemental Figure 13B). In response to DEG-77, p53 accumulation was observed with a corresponding upregulation of p21 in MCF7-Res cells but not in T47D-Res cells, as expected, due to differences in their p53 mutation status. Treatment of either cell line with DEG-77 combined with knockdown of p21 resulted in reduced p21 levels in each of the models (Supplemental Figure 13B). Quantitative analysis of APC/C-Cdh1 inactivation as measured by Geminin-mCherry accumulation showed that, in untreated MCF7-Res and T47D-Res cells, p21 knockdown alone had no measurable effect (Supplemental Figure 13, C–E). As observed previously, DEG-77 treatment alone prevented efficient inactivation of APC/C-Cdh1 in MCF7-Res cells and less so in T47D-Res cells. Compared with treatment with DEG-77 alone, p21 depletion combined with DEG-77 resulted in a significant restoration of APC/C-Cdh1 inactivation, as evidenced by Geminin-mCherry accumulation in 73% of cells, compared to 24% with DEG-77 alone. In contrast, p21 knockdown combined with DEG-77 treatment in T47D-Res cells minimally impacted G1 arrest, supporting the notion that the CK1α-p53-p21 axis contributes to DEG-77–induced inhibition of G1/S transition in a p53-intact context, whereas the absence of functional p53 in T47D-Res cells limits DEG-77 efficacy. This finding further indicated that the observed G1 arrest in 15% of T47D-Res cells upon DEG-77 treatment can be attributed to the noncanonical CK1α-FADD–dependent pathway in this mutant p53 cell line. To provide further support for the role of p21 in DEG-77–mediated cell cycle delay in MCF7 but not T47D cells, overexpression of p21 in T47D cells using a doxycycline-inducible p21 construct (p21 HA) significantly enhanced the G1 arrest induced by DEG-77, confirming the required contribution of the p21 and FADD-dependent pathways to the full antiproliferative response of DEG-77–mediated CK1a degradation (Supplemental Figure 13, F and G).

To confirm that p53 is the upstream regulator responsible for this p21-mediated effect, we next performed direct TP53 knockdown in both MCF7-Res and T47D-Res cells (Supplemental Figure 14, A and B). In MCF7-Res cells, TP53 knockdown reduced the efficacy of DEG-77, increasing the population undergoing APC/C-Cdh1 inactivation from 24% to 69%. In T47D-Res cells, p53 knockdown had no additive effect on the muted DEG-77 response (87% versus 89% cycling), consistent with the sole dependence on the alternate, FADD-mediated pathway (Supplemental Figure 14, C–E).

To define the respective contributions of the p53-p21 cell cycle checkpoint and the FADD-bypass pathway to the response following CK1α degradation, we performed combinatorial knockdown and treatment experiments in MCF7-Res cells (Supplemental Figure 15, A and B). Consistent with its role as a direct APC/C-Cdh1 inhibitor and essential component of the alternate pathway, FADD knockdown alone significantly reduced the percentage of cells undergoing APC/C-Cdh1 inactivation and prolonged G1 phase duration. Knockdown of the cell cycle inhibitor p21 alone had no measurable effect on cell cycle progression under basal conditions (Supplemental Figure 15, C–E). Treatment with the combination of DEG-77 and CDK4/6i (4/6i) induced a near-complete G1 arrest. Notably, concomitant p21 knockdown in this context partially restored the ability of cells to inactivate APC/C-Cdh1 and enter S phase. In contrast, FADD knockdown combined with DEG-77 and 4/6i sustained a complete and durable G1 arrest. The phenotype of the triple combination (DEG-77+4/6i with p21 and FADD co-knockdown) was indistinguishable from that observed with DEG-77+4/6i plus p21 knockdown (Supplemental Figure 15, C–E). These data demonstrated that CK1α degradation enforces a robust G1 arrest through 2 convergent, nonredundant mechanisms: disabling the FADD-dependent bypass pathway and activating the canonical p53-p21 checkpoint.

### DEG-77 reversed the CDK4/6i-resistant phenotype in a mouse model of breast cancer.

**Figure 9:**
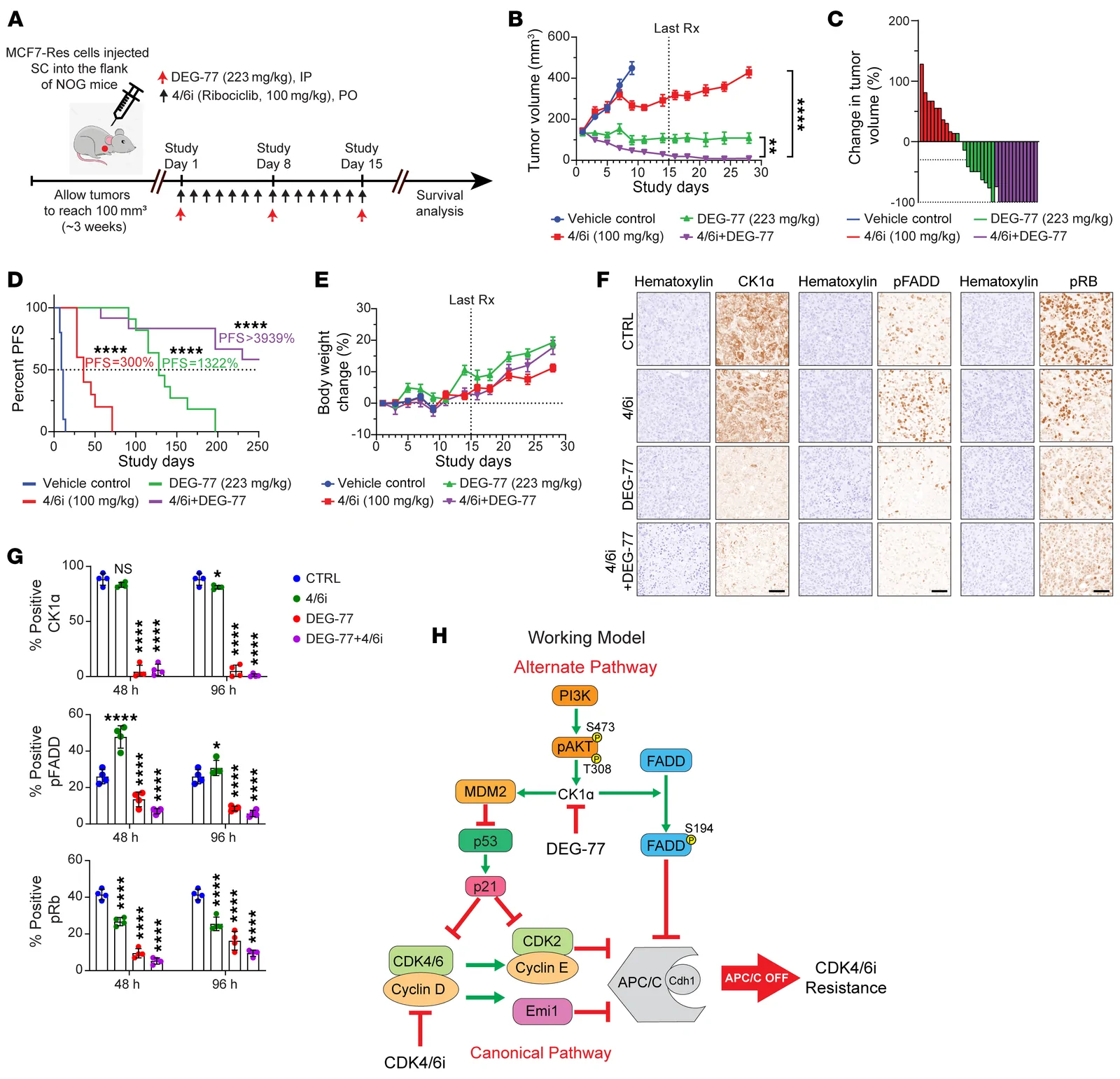
DEG-77 in combination with ribociclib improves progression-free survival in a CDK4/6i-refractory preclinical model of ER^+^/HER2^–^ breast cancer. (**A**) Schematic of the in vivo study design using NOG mice bearing MCF7-Res xenografts. (**B**) Mean tumor volume (± SEM) over time during the 14-day treatment period. (**C**) Waterfall plot of best tumor volume response at day 15. (**D**) Kaplan-Meier curves for progression-free survival (time to endpoint, tumor volume ≥ 500 mm^3^). (**E**) Mean body weight (± SEM) over the treatment period. (**F**) Representative IHC images of CK1α, pFADD(S194), and pRb in tumors 48 hours after treatment. Scale bar: 50 μm. (**G**) Quantification of IHC staining intensity from **F**. Four ROIs per animal, *n* = 3–4 animals/group. (**H**) Working model illustrating the canonical G1/S pathway, the PI3K/CK1α/pFADD-mediated bypass pathway driving CDK4/6 inhibitor (CDK4/6i) resistance, and the mechanism by which combined CK1α degradation (DEG-77) and CDK4/6i restores cell cycle arrest. Drug doses: Ribociclib, 100 mg/kg (oral, QD); DEG-77, 223 mg/kg (IP, Q3D). For **B** and **E**, 2-way repeated-measures ANOVA with Šídák’s correction was used; for **D**, log-rank Mantel-Cox test with Holm-Šídák’s correction was used; for **G**, 1-way ANOVA with Tukey’s correction was used. *P < 0.05; **P < 0.01; ***P < 0.001; ****P < 0.0001.

To investigate the effects of DEG-77 on the CDK4/6i-resistant phenotype in a preclinical mouse model, NOG mice were implanted with MCF7-Res cells as described previously (Figure 9A) (74, 75). As expected, treatment with ribociclib as a single agent was ineffective in MCF7-Res tumor-bearing animals, producing minimal antitumor efficacy (Figure 9B), as reflected by a day 15 ΔT/ΔC value of 46% and no objective tumor responses (Figure 9C). Treatment with DEG-77 as a single agent resulted in a 64% and 9% incidence of partial and complete tumor regressions, respectively, leading to a prolonged median progression-free survival (1,322%) (Figure 9D). Consistent with our in vitro findings, the combination of DEG-77 and ribociclib led to a dramatic improvement in efficacy, reflected by a 92% incidence of complete tumor regressions. Treatment of MCF7-Res tumor-bearing animals with ribociclib, DEG-77 and their combination was well tolerated, as evidenced by the absence of differences in mouse weight throughout the study (Figure 9E) and no obvious clinical signs of toxicity. Objective tumor responses in the combination treatment arm were more durable, and regrowth was significantly slower than in the mice that received DEG-77 monotherapy contributing to prolonged median progression-free survival (4,000%) in mice treated with the combination. Extended monitoring revealed that tumor regrowth was profoundly delayed in the combination group, with most tumors remaining small for months before progression (Supplemental Figure 16, A and B). Together, these functional studies showed that inhibition of the FADD-dependent pathway restored CDK4/6i sensitivity in both resistant cell line models tested, while combined CK1α degradation and CDK4/6 inhibition produced complete regressions in 92% of MCF7-Res xenografts. Pharmacodynamic analysis of CK1α and phospho-FADD levels in MCF7-Res tumors showed a marked reduction in both proteins at 48 and 96 hours following a single treatment of DEG-77 or the combination of DEG-77 and ribociclib. In contrast, ribociclib monotherapy resulted in no change in CK1α expression and a modest increase in phospho-FADD levels. pRb was also reduced with DEG-77 monotherapy as well as the combination of DEG-77 and ribociclib (Figure 9, F and G, and Supplemental Figure 16, C and D), mirroring our in vitro observations where MCF7-Res cells exhibited significant Rb phosphorylation loss when exposed to these treatments. This reduction in pRb levels paralleled the in vitro findings of diminished Rb phosphorylation and supported a role for FADD-mediated APC/C-Cdh1 inactivation as a mechanism facilitating G1/S transition, despite the loss of CDK4/6 activity.

## Discussion

In response to CDK4/6 activation, phosphorylation of Rb initiates the release of E2F from Rb inhibition (29, 76–78). The E2F transcription factor drives the expression of Emi1 and Cyclin E, which mediates APC/C-Cdh1 inactivation. Inactivation of APC/C-Cdh1 is an important control point (restriction point) in the cell cycle and a requisite for the G1 to S phase transition (78, 79). Our analysis of the G1/S cell cycle checkpoint in CDK4/6i-resistant cell lines revealed a breach, wherein continued APC/C-Cdh1 inactivation and cell proliferation was observed, despite the absence of CDK4/6 activity, Rb phosphorylation, and E2F activity, prompting us to identify an alternate mechanism for APC/C-Cdh1 inactivation.

We found that the phosphorylated form of FADD (phospho-FADD) provided an alternate mechanism for APC/C-Cdh1 inactivation in the models examined bypassing the requirement for the canonical CDK4/6-Rb-E2F pathway for G1 to S transition (Figure 9H). An increase in PI3K signaling, enhanced FADD phosphorylation as well as continued APC/C-Cdh1 inactivation and entry into the cell cycle even in the absence of CDK4/6 activity was a characteristic of each of the CDK4/6i-resistant cell lines developed. The PI3K/AKT/mTOR pathway is best appreciated as a driver of recurrence (24–27, 55), and a basis for the recent approval of combination treatment with a PI3Kα inhibitor (inavolisib), CDK4/6 inhibitor (palbociclib), and endocrine therapy (fulvestrant), in patients with PIK3CA-mutated, hormone receptor–positive, HER2-negative locally advanced or metastatic breast cancer (28). In support, our analysis of CDK4/6-refractory cell lines as well as tumor biopsy at recurrence after CDK4/6i revealed that activation of the PI3K/AKT/mTOR pathway was consistently observed upon the development of resistance. However, a molecular understanding of how enhanced signaling through the PI3K/AKT/mTOR pathway effectuates a CDK4/6-independent mechanism for entry into S from G1 is lacking. Previous studies (80, 81) provide support for our finding that differences in cell cycle regulators give rise to alternate cell cycle “paths” that allow individual tumor cells to escape CDK4/6i treatment.

We also found that inhibition of PI3K/AKT signaling resulted in a simultaneous decrease in levels of phospho-FADD (but not total FADD) and restoration of the G1/S checkpoint in CDK4/6i-resistant cells, providing a mechanistic basis for phospho-FADD’s role as an effector for overcoming the G1/S checkpoint and contributor to a CDK4/6 inhibitor-resistant phenotype. Combining PI3K-inhibition with CDK4/6 inhibition resulted in an almost complete loss in the capacity for APC/C-Cdh1 inactivation and restoration of sensitivity to CDK4/6 inhibitors. A careful temporal analysis of the G1 to S cell cycle transition at a single cell resolution in the present study supports our model for the existence of a phospho-FADD–mediated, CDK4/6-Rb-E2F–independent pathway for APC/C-Cdh1 inhibition as a mechanism contributing to resistance in the models examined in the presence of CDK4/6 inhibitors and fulvestrant, and that enhanced signaling through the PI3K promotes phosphorylation of FADD.

Casein kinase 1α (CK1α) mediates the phosphorylation of FADD at Ser194 (42, 49, 53). This phosphorylation drives its nuclear localization (50, 54). We observed a pronounced nuclear localization of FADD in resistant cell lines as well as paired breast cancer patient biopsies. Pretreatment and postrecurrence-paired biopsies demonstrated a dramatic increase in levels of phosphorylated FADD and PI3K signaling at recurrence compared with pretreatment samples in 8 of 11 patients evaluated. Most interestingly, the 3 patients who presented with elevated phospho-FADD and phospho-AKT staining at initial diagnosis with no further elevation at time of progression were the only patients studied who derived no clinical benefit from CDK4/6i treatment (abemaciclib or ribociclib). All 3 of these refractory patients demonstrated disease progression at the time of their first restaging scan (within 3 months of initiating CDK 4/6i therapy). An association of enhanced PI3K/AKT/mTOR signaling in CDK4/6i refractory cells and clinical samples with increased levels of phospho-FADD suggests that CK1α -mediated FADD phosphorylation is downstream of the PI3K signaling pathway and that FADD’s function as an APC/C-Cdh1 inhibitor may contribute to a CDK4/6i-resistant phenotype associated with PI3K pathway activation.

DEG-77, a CK1α degrader, demonstrated that inhibition of CK1α activity significantly reduced FADD phosphorylation, correspondingly decreasing the efficiency for APC/C-Cdh1 inactivation and cell proliferation, supporting a functional role for FADD phosphorylation in CDK4/6i resistance. Most notably, the combination of CDK4/6i with DEG-77 led to a robust G1 arrest in parental and resistant cell lines, highlighting the potential of simultaneous targeting of both canonical and alternative pathways for APC/C-Cdh1 inactivation to effectively overcome resistance. Future therapeutic strategies could leverage these insights, aligning with other studies advocating for dual-pathway inhibition in resistant cancer types. The combined treatment using DEG-77 and CDK4/6 inhibitors led to a sustained suppression of cell proliferation lasting over 15 days and induction of senescence. Given the role of CK1α in regulating p53 activity through an MDM2/4-dependent mechanism, we explored whether the observed cell cycle arrest and senescence were mediated by the p53-p21 axis. Two key observations support the significance of the CK1α-p53-p21 pathway in DEG-77’s effects: first, the efficacy of DEG-77 was reduced in the p53-mutant T47D cell line compared with MCF7, highlighting p53’s importance; second, siRNA-mediated p21 depletion in MCF7-Res cells significantly decreased DEG-77–induced G1 arrest, providing support for the role of p21 in mediating CDK2 inactivation. These findings highlight the potential of leveraging the p53-p21 pathway in combination therapies to enhance treatment efficacy, particularly in p53 WT tumors.

Xenograft mouse model studies incorporating the use of DEG-77 provides strong preclinical support for targeting CK1α to counteract the CDK4/6 inhibitor-resistant phenotype often seen in breast cancer patients. Finally, the clinical implications of these findings are substantial. First, FADD phosphorylation levels could serve as a candidate biomarker for inherent CDK4/6i resistance, aiding in the stratification of patients who are unlikely to benefit from standard of care CDK4/6i-based therapy. Second, the combination of CDK4/6 inhibitors with CK1α degraders represents a promising therapeutic strategy to overcome resistance. Preclinical studies demonstrate that this combination approach not only restores cell cycle arrest but also promotes durable antitumor responses through senescence and reduced tumor growth in xenograft models. These findings pave the way for the rational design of clinical trials to evaluate this combination approach in patients with CDK4/6i-resistant breast cancer. The current standard of care combines CDK4/6i with endocrine agents like the SERD fulvestrant. Both modalities converge on inhibiting the canonical cyclin D-CDK4/6-Rb axis; fulvestrant by down regulating cyclin D1 expression and CDK4/6i by directly inhibiting kinase activity of cyclin-CDK4/6 complex. Our work reveals that the PI3K/CK1α/pFADD alternate pathway for G1 to S transition operates independently of this axis, defining a targetable resistance mechanism that is likely to persist and drive progression even after the development of endocrine resistance. However, the prevalence and biomarker utility of this mechanism require further definition in larger patient cohorts and resistant model panels.

## Methods

### Sex as a biological variable.

This study utilized female mice for xenograft experiments, consistent with modeling estrogen receptor-positive (ER^+^) breast cancer.

### Cell lines and animals.

MCF7 (HTB-22) and T47D (HTB-133) cells were obtained from ATCC, HEK293FT cells were obtained from Thermo Scientific, and MCF7-Res and T47D-Res cells were generated in-house by chronic ribociclib selection as described in Supplemental Methods. Female 6–8-week-old CIEA NOG mice were obtained from Taconic.

### Reagents.

Antibodies and inhibitors used in this study are listed in Supplemental Tables 2–4, including source, catalog/clone number, application, dilution, and working concentration.

### Study approval.

All animal procedures were performed under IACUC-approved protocol PRO00010288. The use of human patient biopsy samples was approved by the University of Michigan Institutional Review Board (IRB00005467), with consent requirements handled according to the approved protocol.

### Data availability.

Values for all data points shown in graphs are provided in the Supporting Data Values file. Custom image-analysis scripts used for PIP-FUCCI quantification are available at GitHub: https://github.com/sahezeel/PIP-FUCCI-Analysis-codes All other data supporting the findings of this study are available within the article and its supplemental material.

### Statistics.

Statistical tests, *n* values, and definitions of replicates are provided in the figure legends. Data are presented as mean ± SEM unless otherwise indicated. For 2-group comparisons, 2-tailed Student’s *t*-test with Welch’s correction was used unless otherwise indicated. For comparisons involving more than 2 groups, 1-way or 2-way ANOVA with appropriate multiple-comparison correction was used, as specified in the figure legends. For paired patient biopsy comparisons, paired statistical testing was used as indicated. Survival probabilities were estimated using the Kaplan-Meier method and compared using the log-rank test. *P* values less than 0.05 were considered statistically significant.

## Author contributions

SA and AR conceptualized the study. SA and AR developed the methodology. SA, EKZ, NMC, EK, JT, AZ, MJM, SR, CG, CMW, CWS, JSL, and AR performed validation. SA, EK, JT, and MJM performed formal analysis. SA, EKZ, NMC, EK, RAZ, JT, MJM, SR, RAZ, CG, CMW, CWS, JSL, and AR performed investigations. SA and AR wrote the original draft. All authors reviewed and edited the manuscript. SA, EKZ, CMW, CWS, JSL, and AR contributed to visualization. CG, CMW, CWS, JSL, and AR supervised the work. CMW, CWS, and AR acquired funding.

## Conflict of interest

The authors have declared that no conflict of interest exists.

## Funding support

This manuscript will be deposited in PubMed Central in accordance with the NIH Public Access Policy.

- Department of Defense–US Army grant HT94252310210 (to AR, SA, and CWS).
- Department of Defense–US Army grant HT94252310263 (to CMW).

## Supplementary Material

Supplemental data

Unedited blot and gel images

Supporting data values

## Version 1 07/28/2026

In-Press Preview

## Version 2 09/15/2026

Electronic publication
