# Supplementary material for: Targeting casein kinase 1α-mediated FADD phosphorylation restores sensitivity in select CDK4/6 inhibitor-resistant breast cancer models
Source: J Clin Invest. 2026 Jul 28;136(18):e199483. doi: 10.1172/JCI199483 (PMC13574148; doi:10.1172/JCI199483)
Supplement: Supplementary file 1 [file jci-136-199483-s125.pdf]

# Supplemental Fig 1

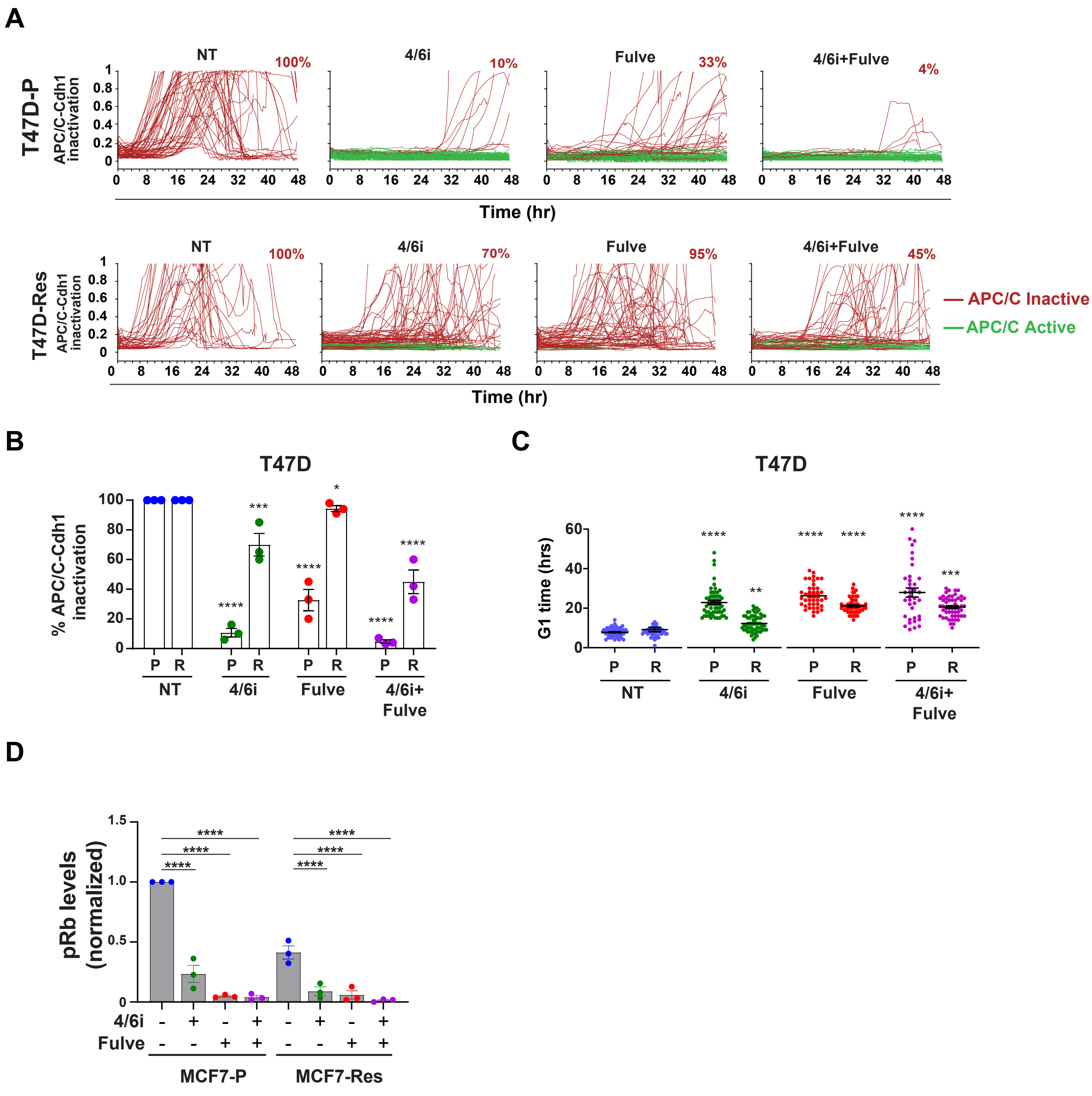

**Supplemental Fig. 1**

**(A)** Live-cell imaging illustrating APC/C-Cdh1 inactivation dynamics and G1 phase duration in T47D-P and T47D-Res cells in response to treatment with CDK4/6i (4/6i), fulvestrant (Fulve), and combination. Representative traces from three biological independent experiments shown, quantitative analysis shown in Figures S1C and S1D. **(B)** A greater percentage of T47D-Res (R) cells experience APC/C-Cdh1 inactivation compared to T47D-P (P) cells in response to CDK4/6i (4/6i), fulvestrant (Fulve), or a combination (4/6i + Fulve). Data (Figures S1A and S1B) are derived from at least three biological replicates. **(C)** Time required for APC/C-Cdh1 inactivation (G1 residence time) post-mitosis is shorter in T47D-Res (R, derived from Figures S1B), compared to MCF7-P (P, derived from Figure S1A) in response to CDK4/6i (4/6i), fulvestrant (Fulve), or a combination (4/6i + Fulve), indicating a breach in the G1 to S checkpoint in MCF7-Res.

Drug concentrations CDK4/6i = 200nM, Fulvestrant = 100nM. Error bars represent mean ±SEM. *P < 0.05; **P < 0.01; ***P < 0.001; ****P < 0.0001. Statistical significances are calculated by comparing individual or combination drug treated parental or resistant cell type with their respective NT condition.

# Supplemental Fig 2

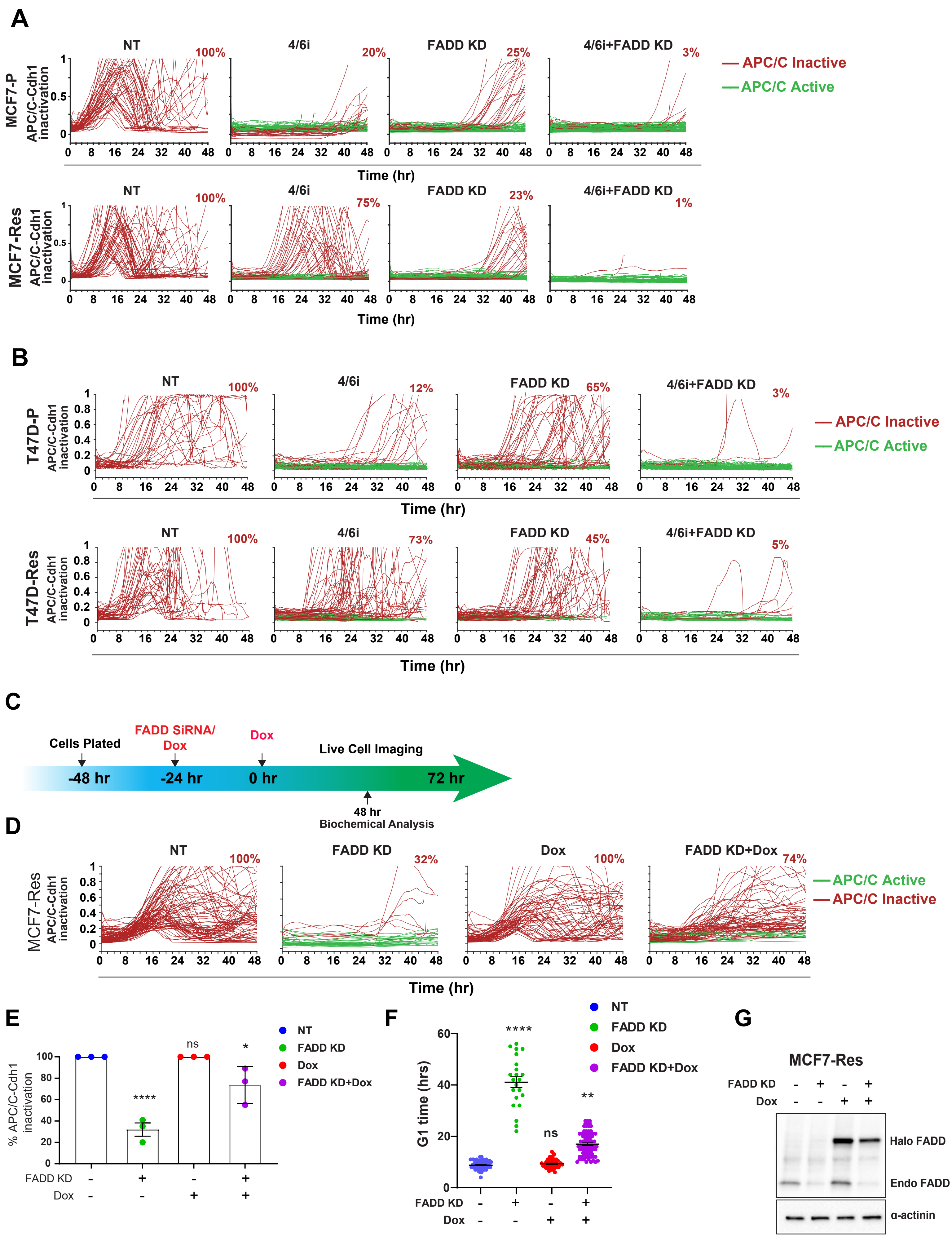

**Supplemental Fig. 2**

**(A)** Single cell traces of control (NT), CDK4/6i treated (4/6i) and/or FADD-depleted (FADD KD) using MCF7-P and MCF7-Res cells. Combined treatment resulted in a failure to inactivate APC/C-Cdh1 (G1 arrest). Representative traces from three biological replicate experiments shown **(B)** Single cell traces of control (NT), CDK4/6i treated (4/6i) and FADD-depleted (FADD KD) T47D-P and T47D-Res cells showing less efficient APC/C-Cdh1 inactivation in response to CDK4/6i treatment in T47D-P but not T47D-Res. FADD depletion using siRNA also resulted in a failure of APC/C-Cdh1 inactivation in either cell line. Combined treatment resulted in an inability to inactivate APC/C-Cdh1 (G1 arrest). Representative traces from three biological replicate experiments shown. **(C)** Schematic of the FADD rescue experiment in MCF7-Res PIP-FUCCI cells. **(D)** Representative single-cell traces of mCherry-Geminin accumulation (indicative of APC/C-Cdh1 inactivation) from the experiment in (C). Traces show cells with FADD knockdown (FADD KD) or FADD knockdown followed by doxycycline-induced re-expression of Halo-FADD (FADD KD + Dox). **(E-F)** Quantification of the percentage of cells that underwent APC/C-Cdh1 inactivation (G1/S transition) and G1 time (hrs) during the imaging period for the conditions shown above. **(G)** Immunoblot confirming efficient knockdown of endogenous FADD and doxycycline-induced re-expression of Halo-FADD.

## Supplemental Fig 3

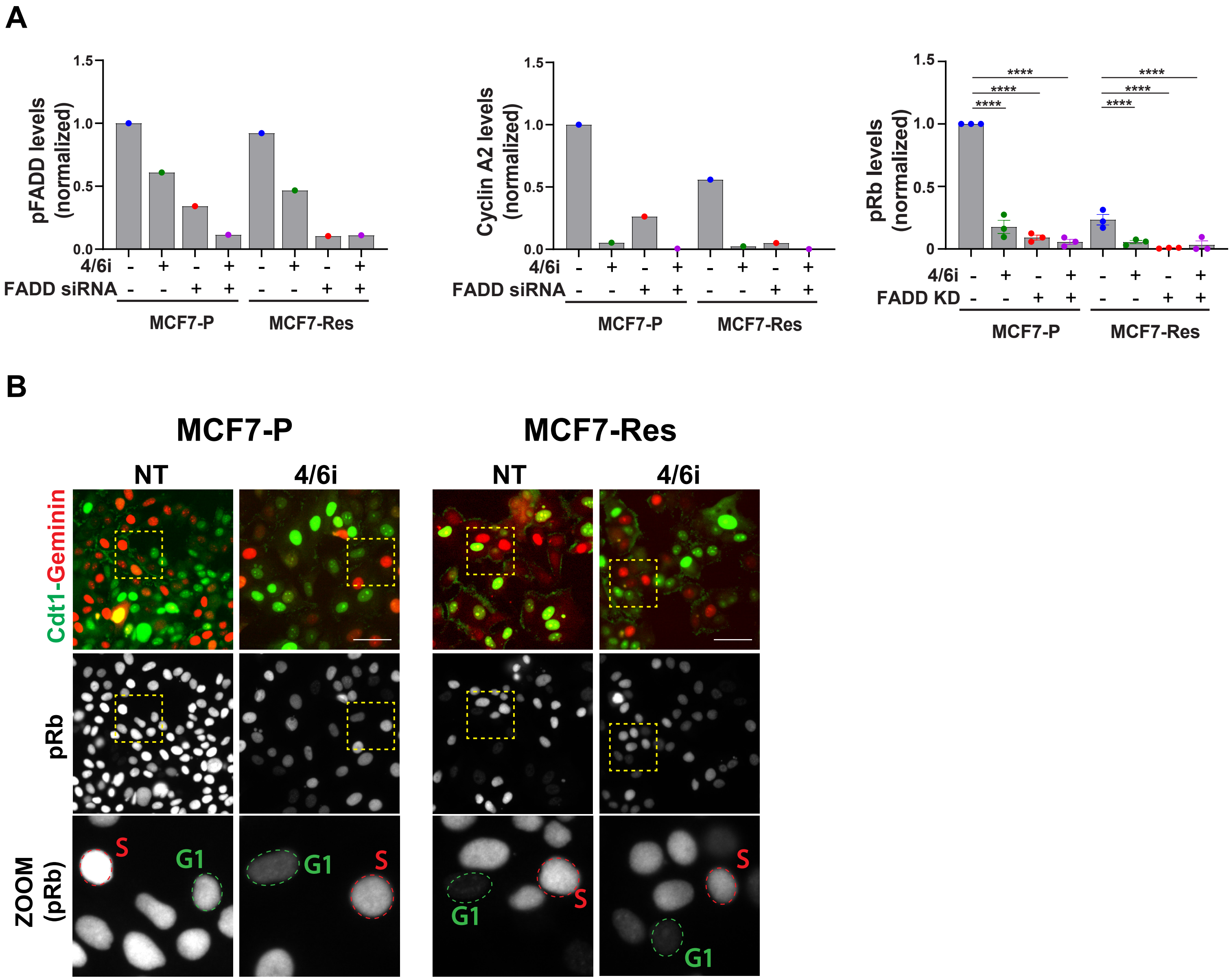

**Supplemental Fig. 3**

**(A)** Quantification of Western blots in Fig. 3G showing pFADD, Cyclin A2 (cyA) and pRB levels in MCF7-P and MCF7-Res cells. **(B)** Immunofluorescence images showing higher pRb staining in untreated MCF7-P cells (NT), and decreased pRb staining in the presence of a CDK4/6i. Lower levels of pRb (compared to MCF7-P NT) were detected in untreated MCF7-Res cells (NT) which was further decreased in the presence of CDK4/6i. Scale bar 30 μm

# Supplemental Fig 4

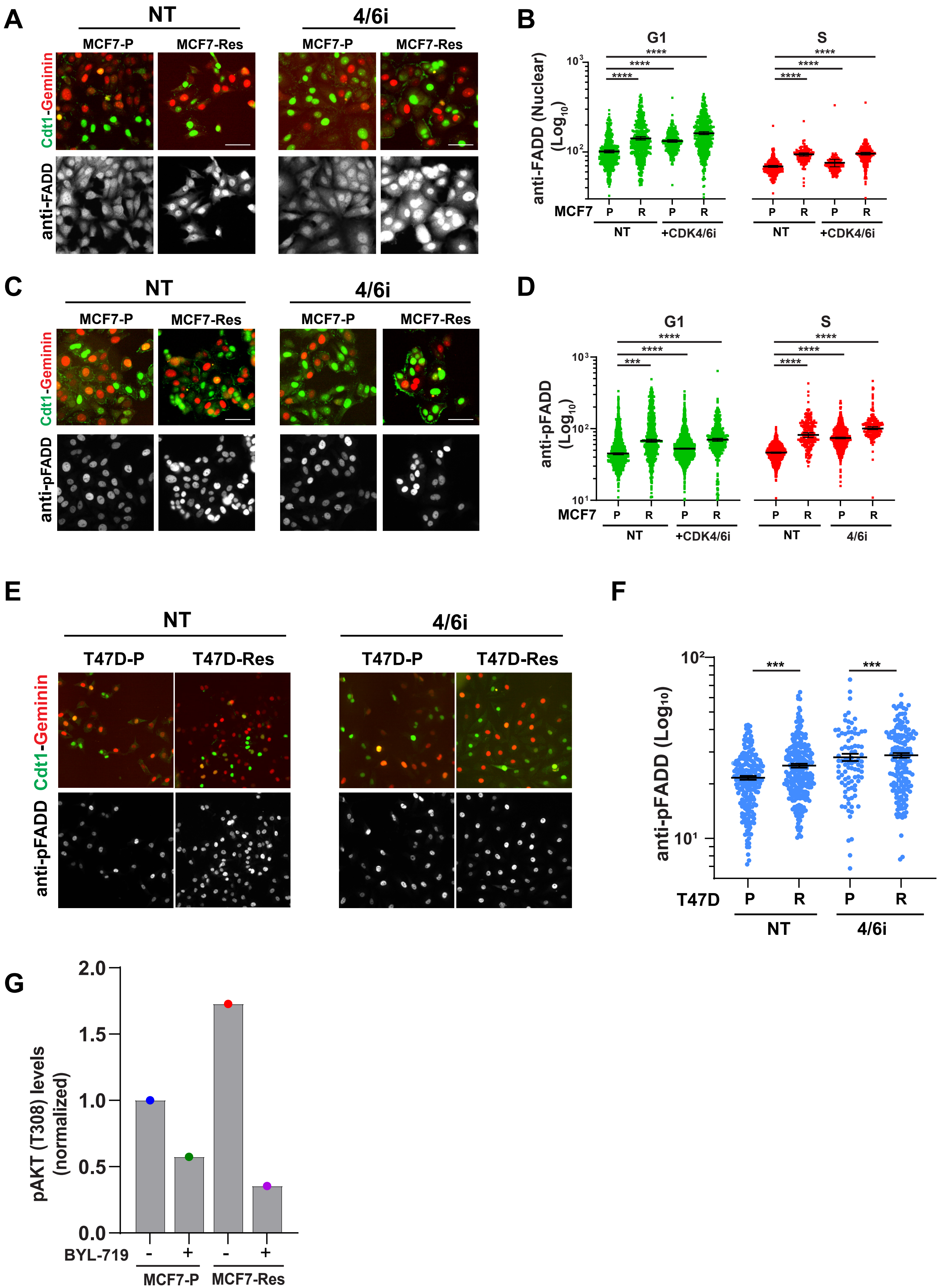

**Supplemental Fig. 4**

**(A)** Immunofluorescence of total FADD in MCF7-P and MCF7-Res cells. Untreated MCF7-P cells show diffuse cytosolic/nuclear staining, while MCF7-Res cells exhibit predominant nuclear FADD in G1 (Cdt1-GFP+, green) and S-phase (Geminin-mCherry+, red) cells. Scale bar 30μm **(B)** Scatter plot quantification of nuclear FADD intensity from (a). Each point represents an individual cell. **(C)** Immunofluorescence of pFADD (S194) in MCF7-P and MCF7-Res cells. MCF7-Res cells show elevated nuclear phospho-FADD levels that increase further with CDK4/6i treatment. Scale bar 30μm **(D)** Scatter plot quantification of nuclear pFADD(S194) intensity from (C). Each point represents an individual cell. **(E)** Immunofluorescence of pFADD(S194) in T47D-P and T47D-Res cells. Resistant cells exhibit enhanced nuclear phospho-FADD localization. Scale bar 30μm **(F)** Scatter plot quantification of nuclear pFADD(S194) intensity from (E). Each point represents an individual cell. **(G)** Quantification of Western blots in Fig. 4F showing pAKT (T308), in MCF7-P and MCF7-Res cells.

## Supplemental Fig 5-1

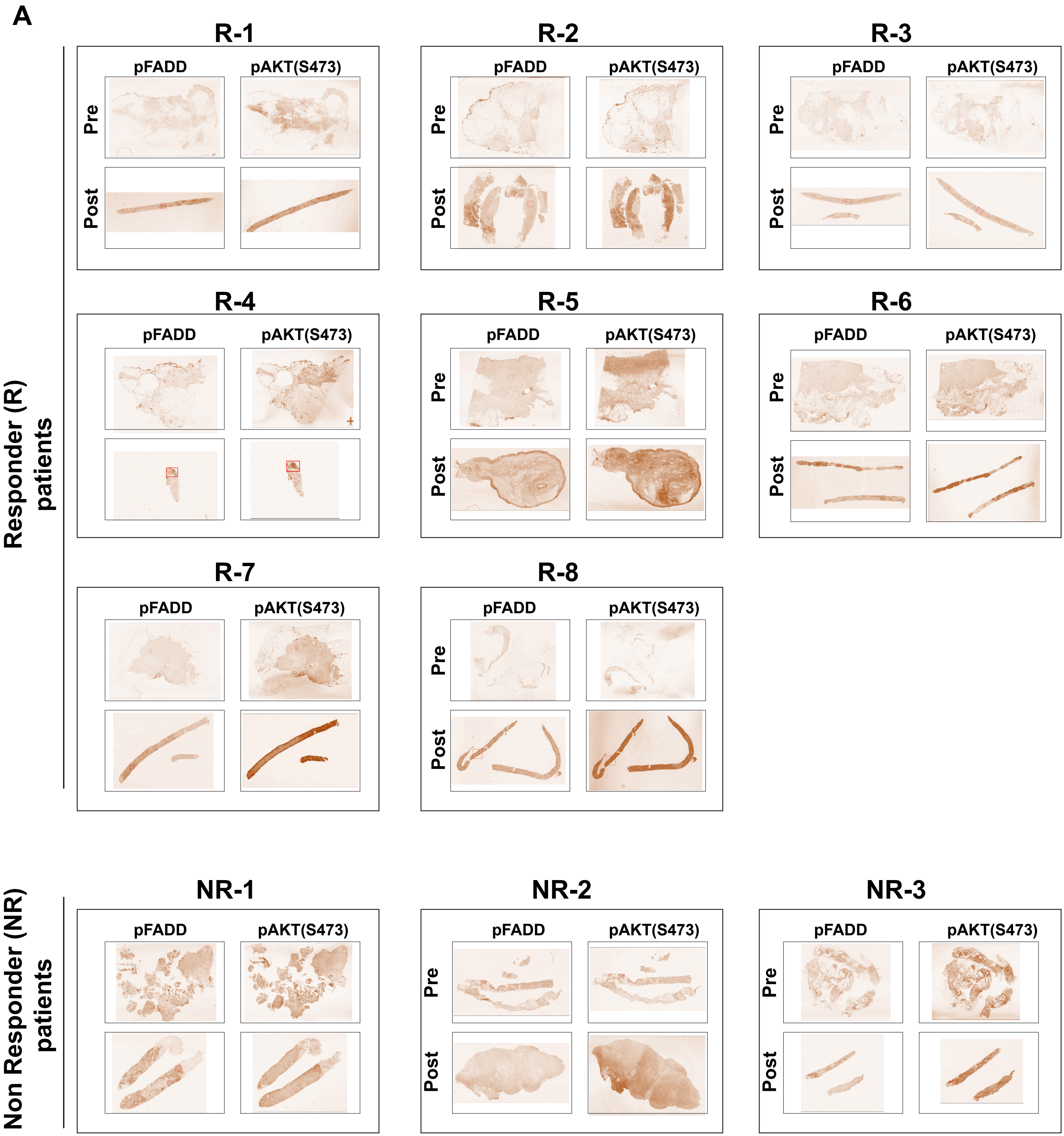

## Supplemental Fig 5-2

**B**

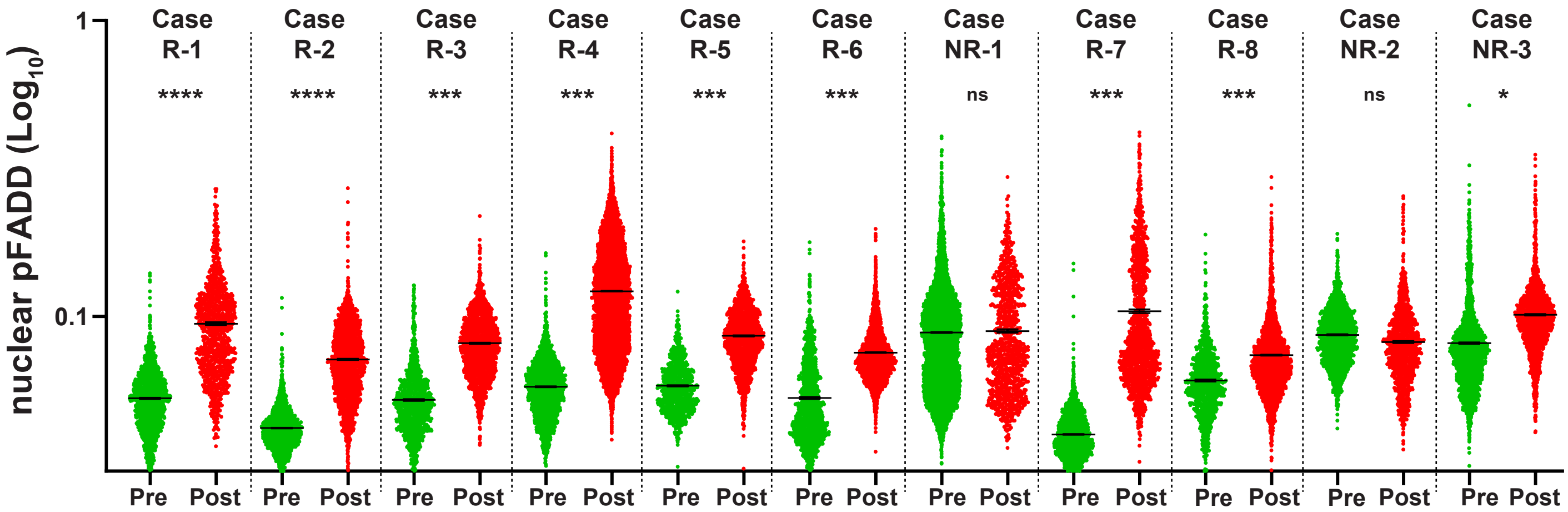


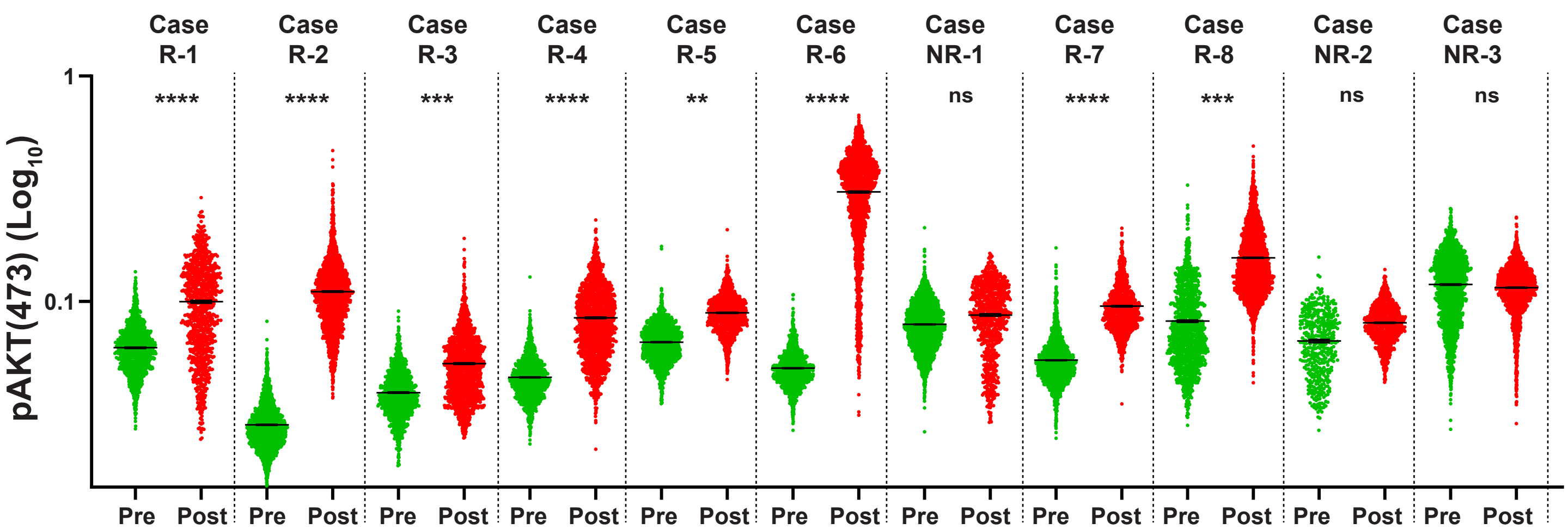


**C**

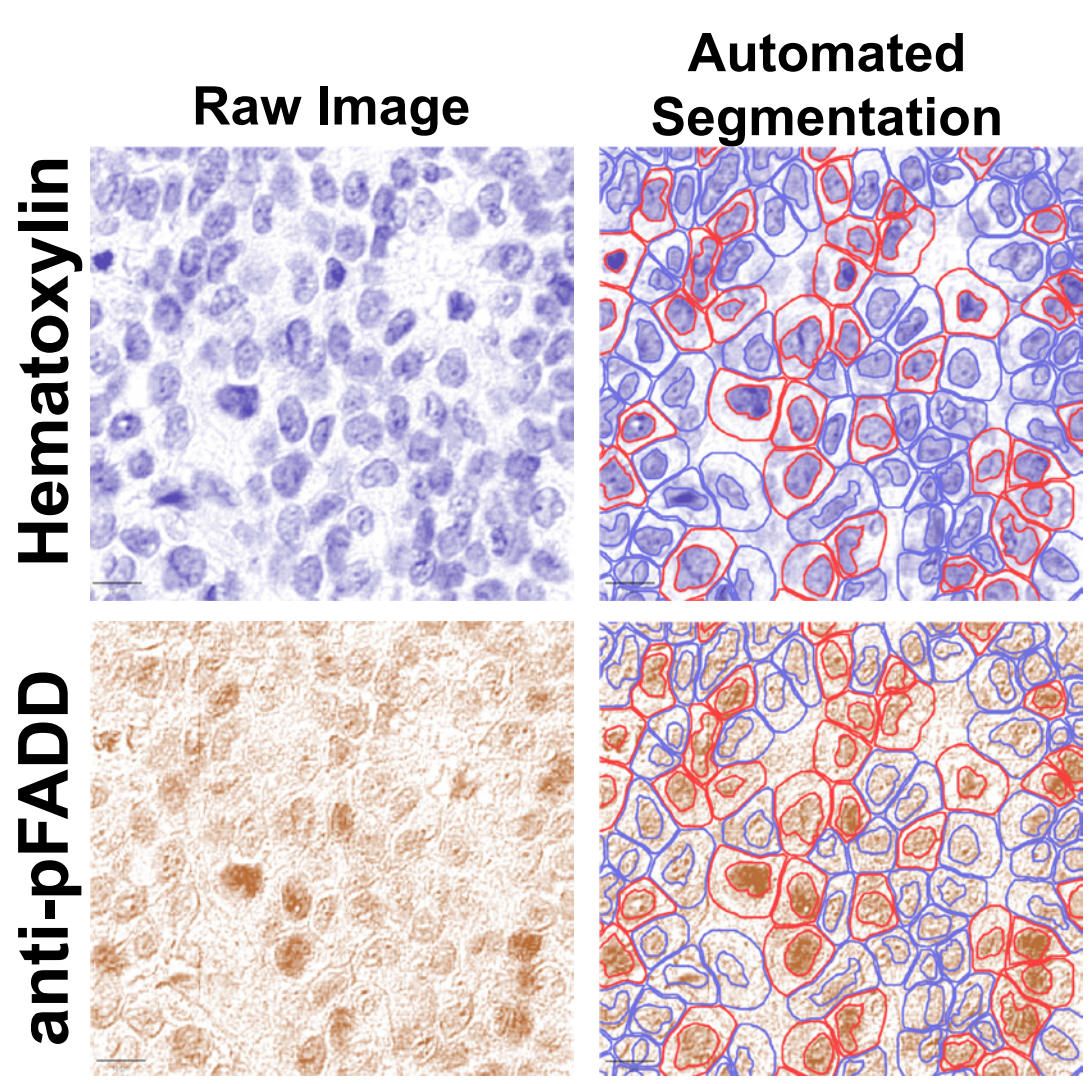

**Supplemental Fig. 5**

**(A)** Low-magnification views of the entire pre- and post-treatment biopsy cores stained for phospho-FADD (Ser194) and phospho-AKT (Ser473), demonstrating the overall staining heterogeneity within tumor sections. Red boxes indicate the approximate areas from which the representative high-power fields in Figure 5A were captured. **(B)** Quantification of pFADD and pAKT-S473 immunohistochemical staining from Figure 5B. Representative post-CDK4/6i patient sample showing Hematoxylin counterstain for nucleus and DAB detection of phospho-FADD. **(C)** Computational segmentation of cellular/nuclear boundaries enabled image quantification (Figure 3D data plotted in panel A above).

## Supplemental Fig 6-1

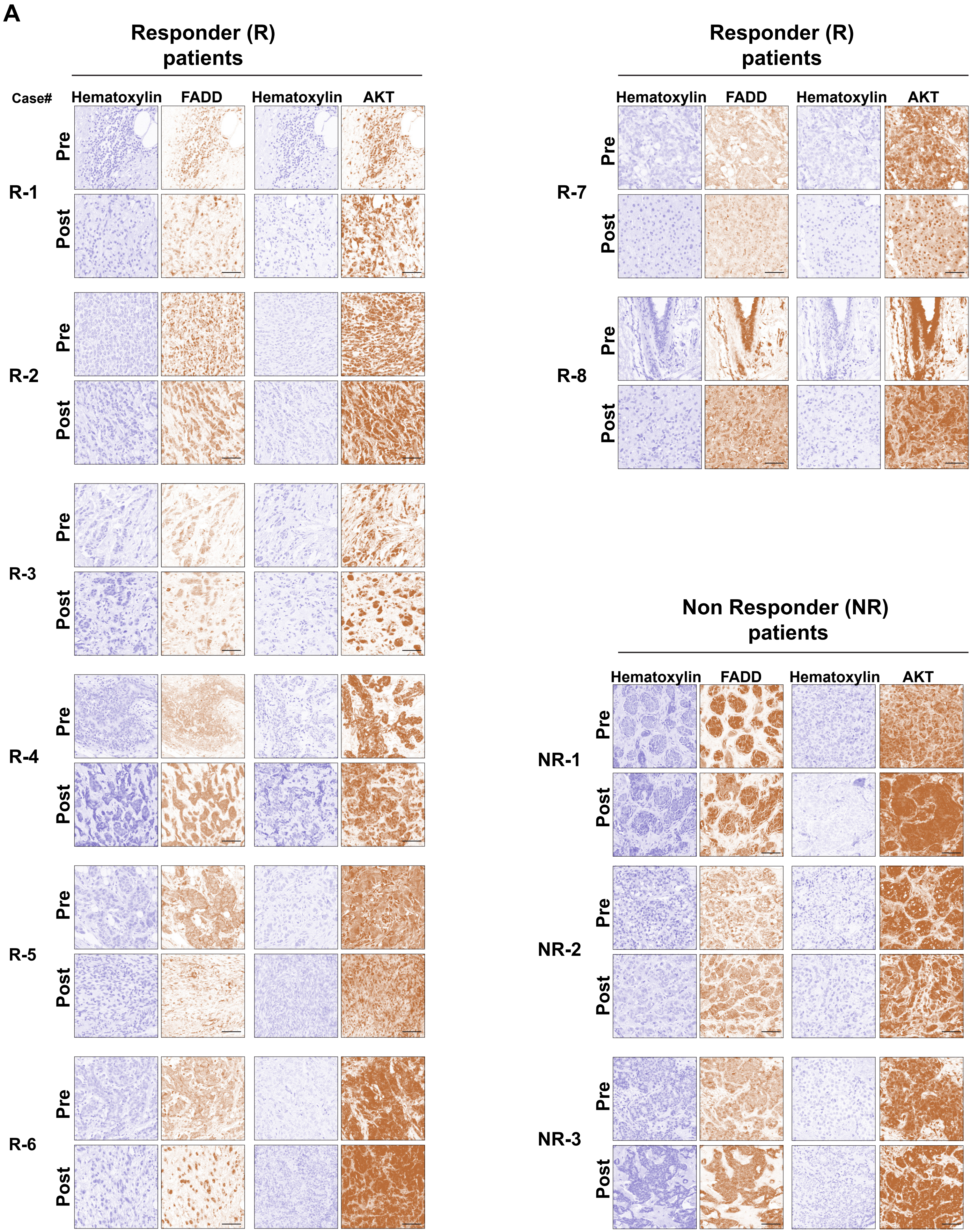

## Supplemental Fig 6-2

B

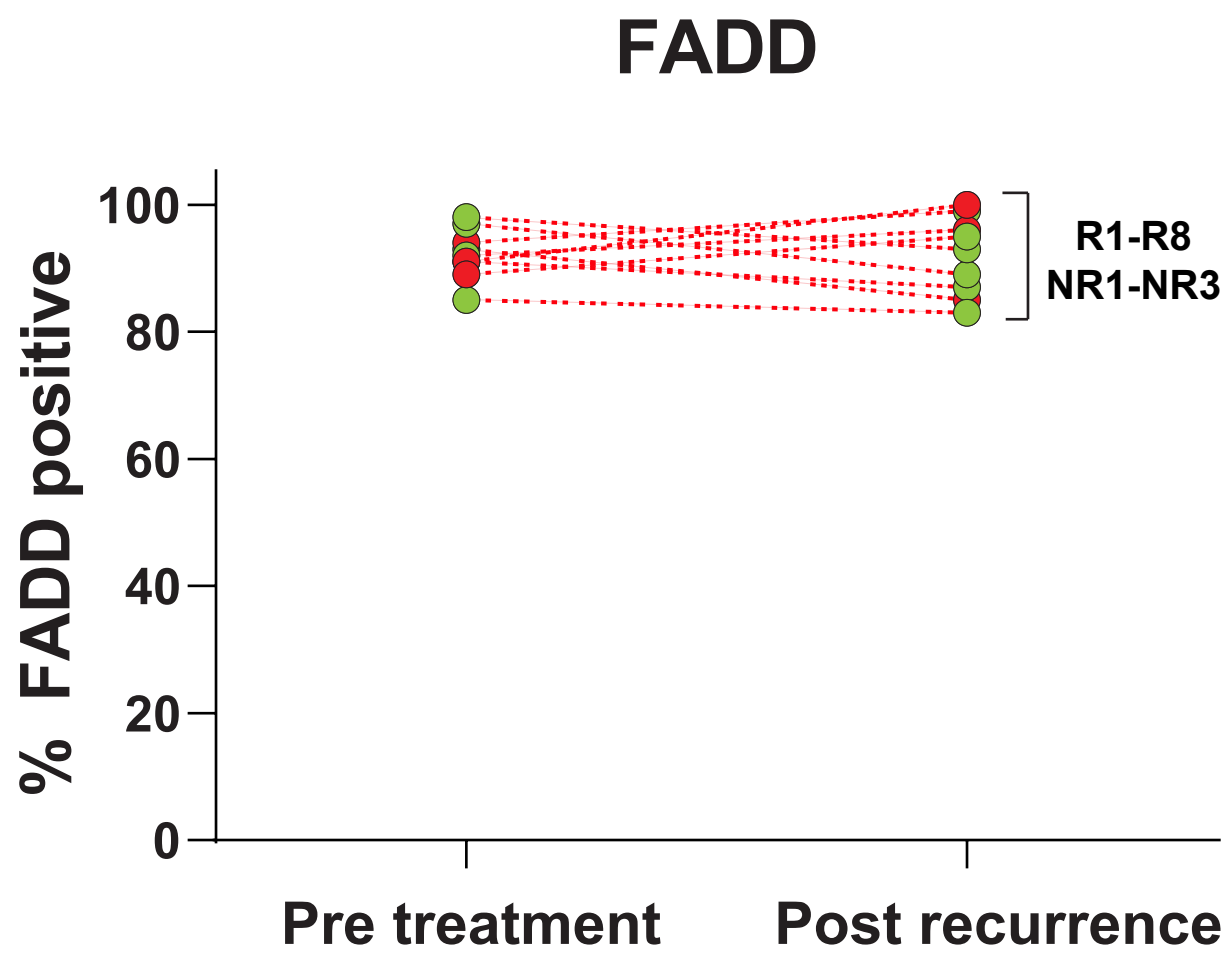


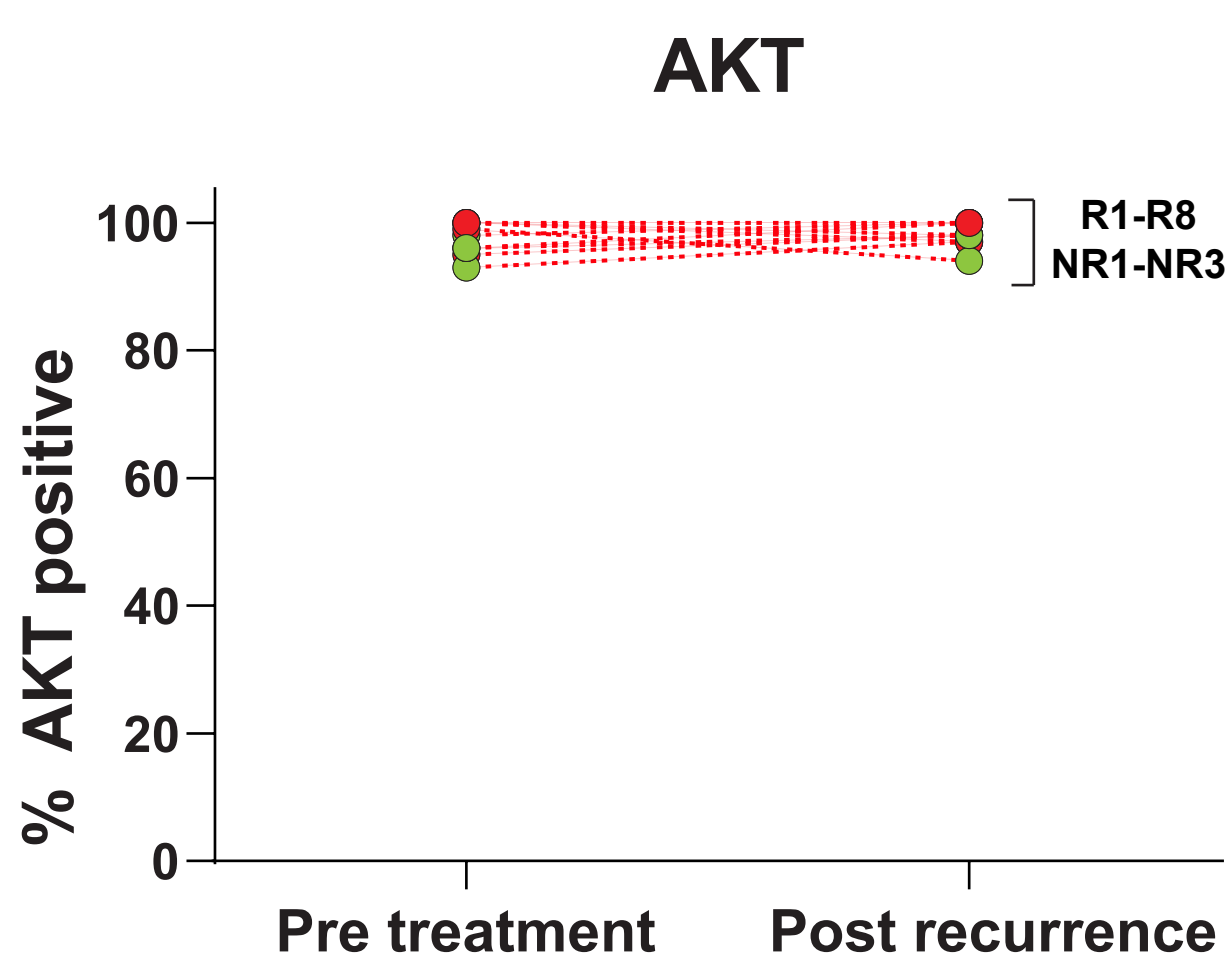

**Supplemental Fig. 6**

**(A)** Images of tumor biopsy immunohistochemical sections stained using a total-FADD or a total-AKT antibody with a corresponding Hematoxylin counterstain section. Scale bar 50 μm. **(B)** Paired dot plots show the percentage of tumor cells positive for total FADD and total AKT in pre-treatment (Pre) and post-progression (Post) biopsies. Each line connects a paired sample from the same patient. Statistical analysis by paired t-test showed no significant change (n.s.).

# Supplemental Fig 7

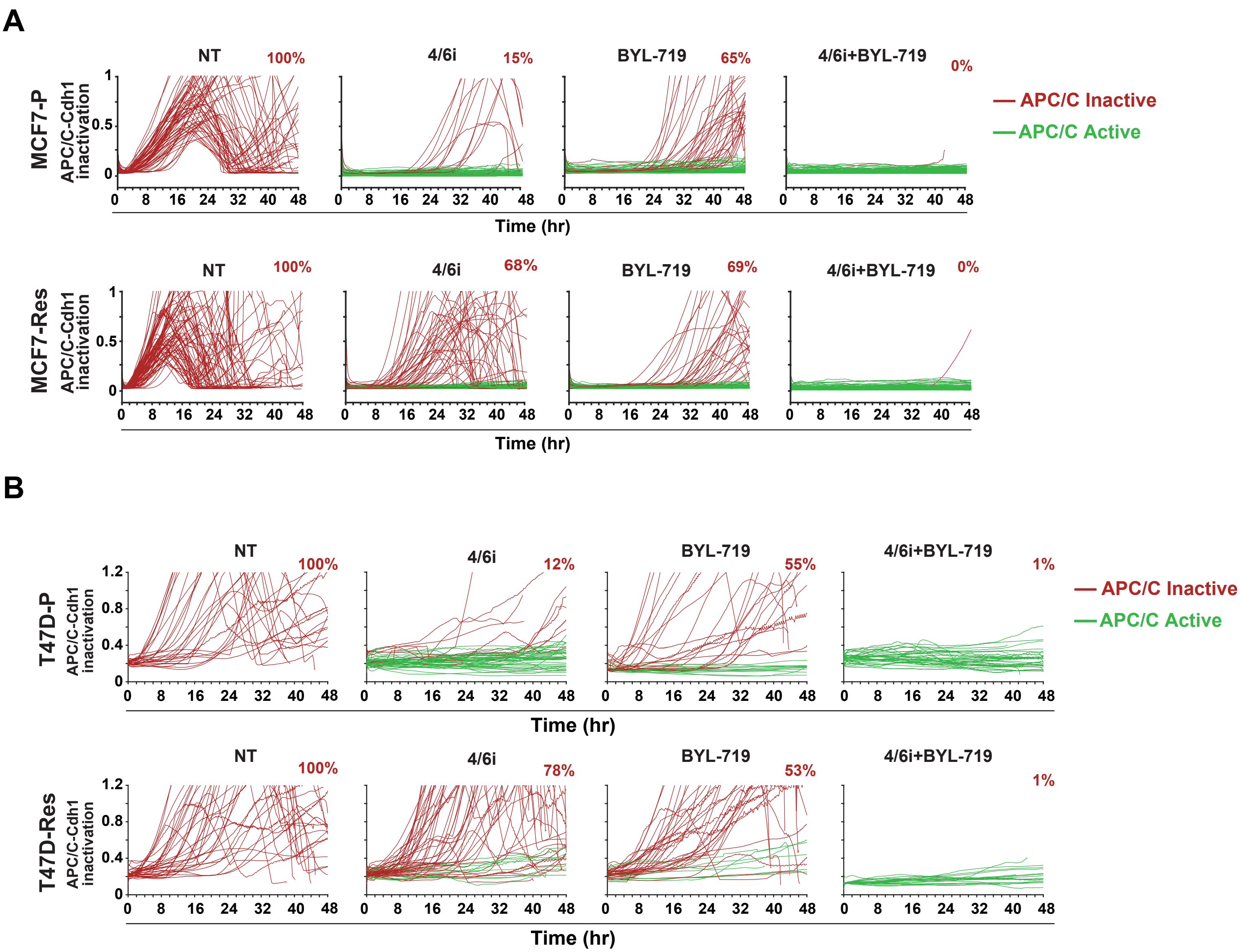

**Supplemental Fig. 7**

**(A)** Non-treated (NT), CDK4/6i treated (4/6i), PI3Kα inhibitor-treated (BYL-719) or combined treatment (4/6i+BYL-719) MCF7-P and MCF7-Res cells were used in a live cell imaging study to evaluate the efficiency and temporal dynamics of APC/C-Cdh1 inactivation over a 48 h period. The percentage of cells experiencing APC/C-Cdh1 inactivation (mCherry accumulation) is shown for each condition. **(B)** Non-treated (NT), CDK4/6i treated (4/6i), AKT-inhibitor treated (BYL-719) or combined treatment (4/6i+BYL-719) T47D-P and T47D-Res cells were used in a live cell imaging study to evaluate the efficiency and temporal dynamics of APC/C-Cdh1 inactivation over a 48 h period. The percentage of cells experiencing APC/C-Cdh1 inactivation (mCherry accumulation) is shown for each condition.

# Supplemental Fig 8

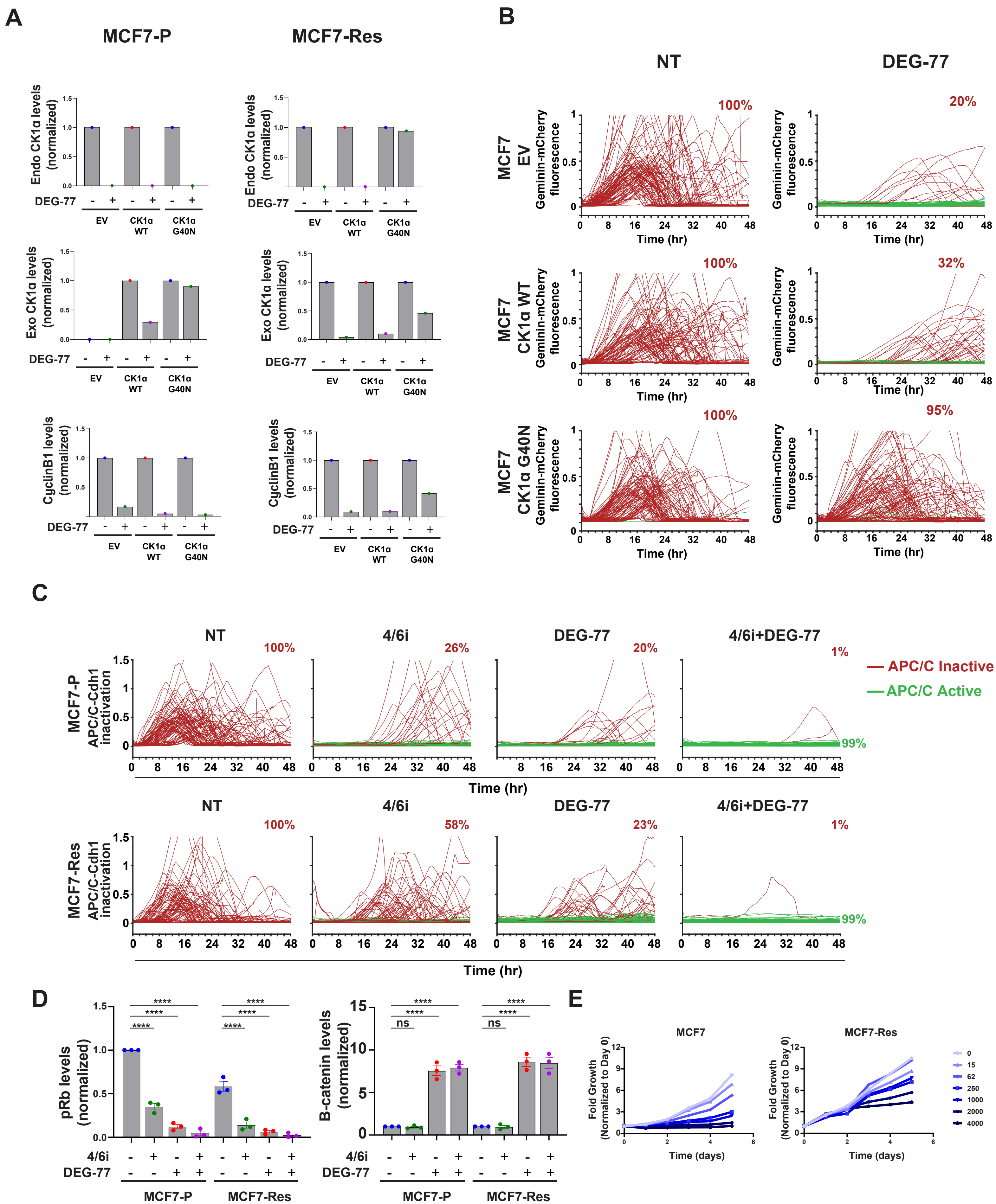

**Supplemental Fig. 8**

**(A)** Quantification of Western blots in Figure 6F showing endogenous CK1α (Endo), exogenous CK1α (Exo), and Cyclin B1 (cyB) levels in EV-, CK1α-WT-, and CK1α-G40N-expressing cells. **(B)** Single cell traces of mCherry-$Gem_{1\text{-}110}$ reporter levels (APC/C-Cdh1 activity) in control (NT), DEG-77 treated MCF7-Res cells stably expressing an empty vector (EV), Wild-type CK1α (CK1α-WT) or a Gly40 to Asparagine mutant form of CK1α (CK1α-G40N). Representative traces from three biological replicate experiments were quantitatively analyzed to derive the percentage of cells experiencing APC/C-Cdh1 inactivation during the imaging time (shown in red). DEG-77 treated CK1α-G40N show minimal impact on APC/C-Cdh1 inactivation efficiency compared to EV or CK1α-WT expressing cells. **(C)** Single cell traces of mCherry-$Gem_{1\text{-}110}$ reporter levels (APC/C-Cdh1 activity) in control (NT), CDK4/6i treated (4/6i) or DEG-77 treated MCF7-P or MCF7-Res cells. Representative traces from three biological replicate experiments shown. **(D)** Quantification of pRb and β-catenin levels as shown in Fig. 8D **(E)** Proliferation of MCF7-P and MCF7-Res cells treated with DEG-77 (0-4000 nM) for 5 days. Data (Mean ± SEM) are normalized to vehicle control.

# Supplemental Fig 9

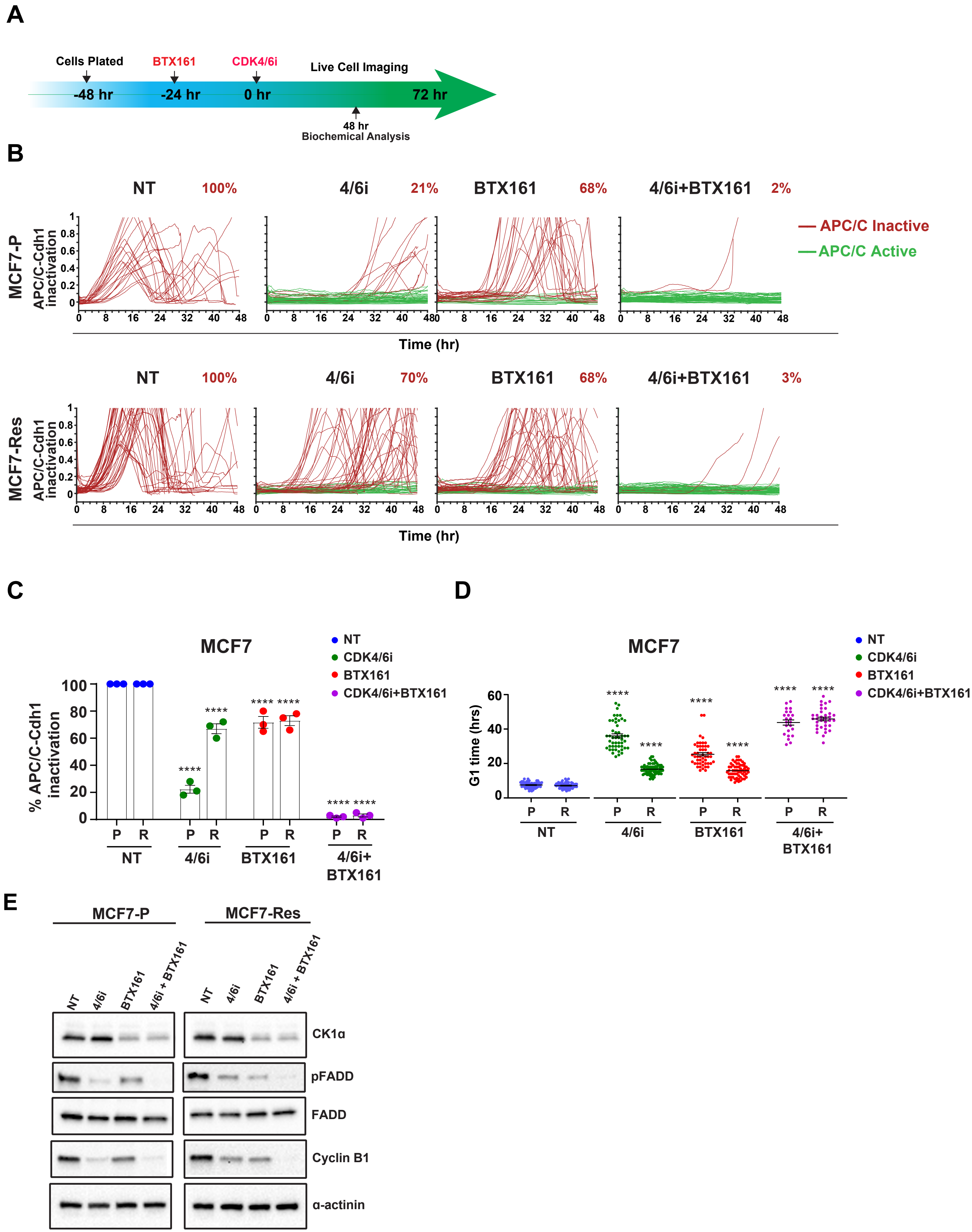

**Supplemental Fig. 9**

**(A)** Experimental outline for BTX161 and CDK4/6i mediated APC/C-Cdh1 inactivation in MCF7-P and MCF7-Res cells using the PIP-FUCCI reporter. **(B)** PIP-FUCCI live-cell imaging illustrating APC/C-Cdh1 inactivation dynamics and G1 phase duration in MCF7-P and MCF7-Res cells under treatment conditions with BTX161, CDK4/6i, and their combination. **(C)** Quantitative analysis (from above) showing that MCF7-P and MCF7-Res cells have similar response of APC/C-Cdh1 inactivation when treated with BTX161 alone whereas CDK4/6i alone demonstrates inefficient G1 to S cell cycle transition (APC/C-Cdh1 inactivation) in MCF7-P compared to MCF7-Res cells. Combination of both agents results in a greatly impaired ability to inactivate APC/C-Cdh1 (i.e. G1 arrest) in either cell line (panel C). **(D)** Scatter plot of G1 times shows a shorter G1 residence time in MCF7-Res cells treated with CDK4/6i alone, while both MCF7-P and MCF7-Res cells exhibit equivalent G1 times when treated with BTX161 alone. Combined treatment with both agents resulted in a protracted time in G1 due to an inability to inactivate APC/C-Cdh1. **(E)** Western blot analysis of MCF7-P and MCF7-Res cells showing levels of CK1α, Cyclin B1, phospho-FADD and FADD upon treatment with CDK4/6i and BTX161, alone or in combination.

# Supplemental Fig 10

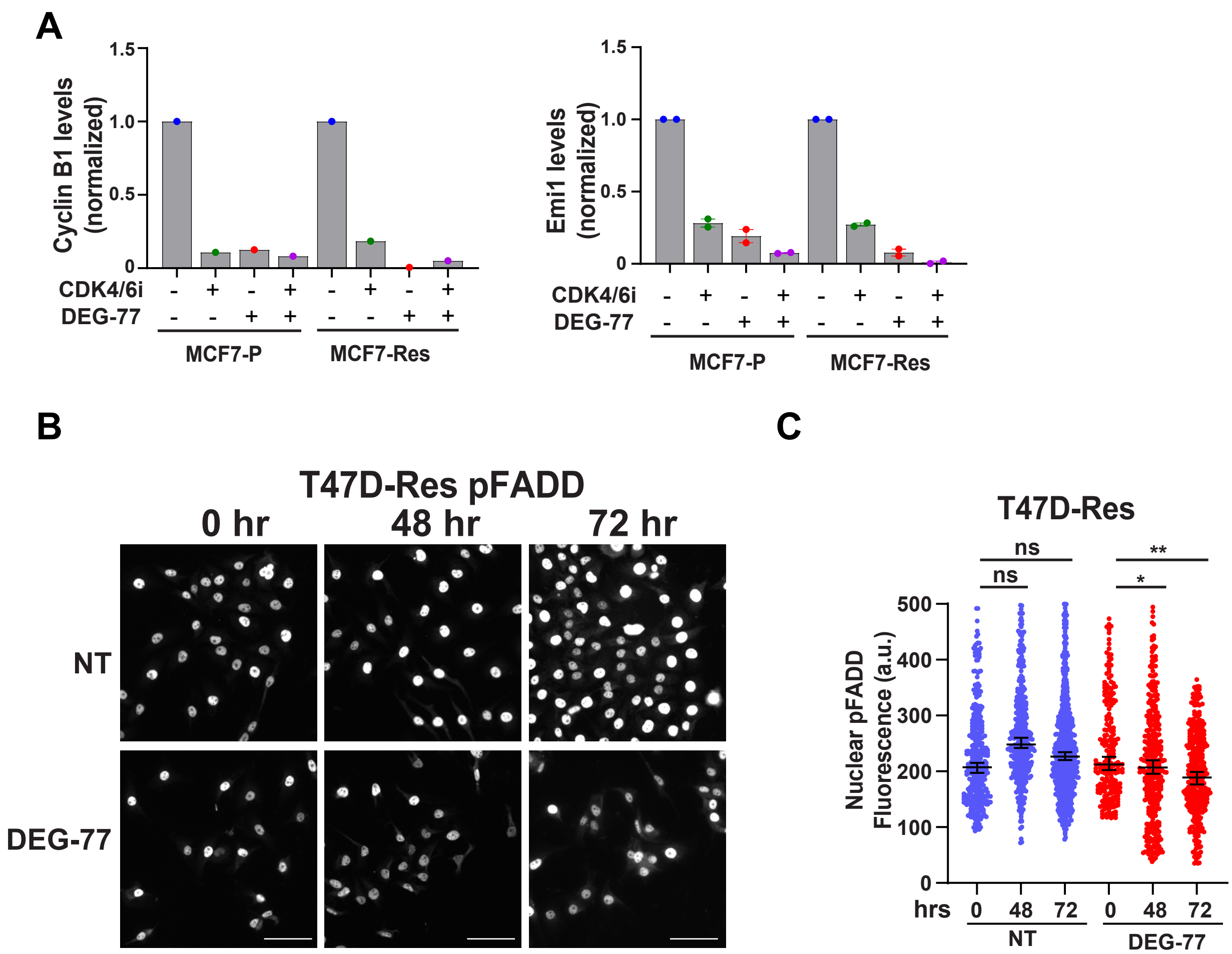

**Supplemental Fig. 10**

**(A)** Quantification of Western blots in Figure 8D showing Cyclin B1 (cyB), and Emi1 levels in MCF7-P and MCF7-Res cells. **(B-C)** Immunocytochemistry analysis demonstrates a decrease in pFADD levels in T47D-Res cells at 48 and 72 h post DEG-77 treatment. Quantification of immunocytochemistry shown in panel C. Scale bar 30μm.

# Supplemental Fig 11

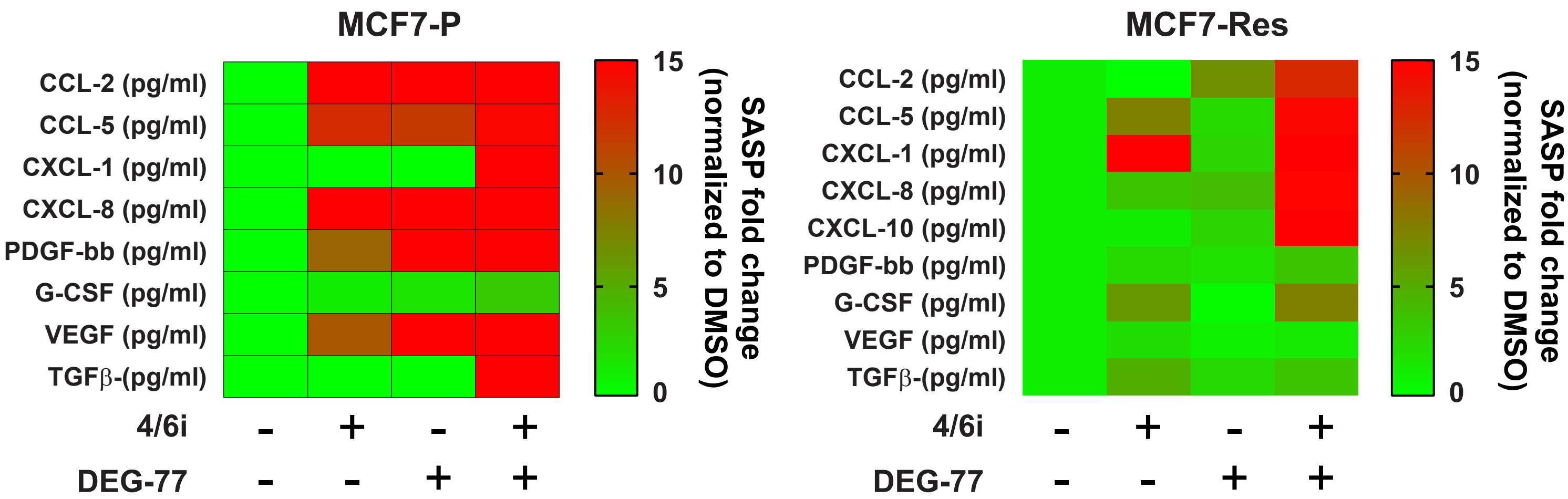

**Supplemental Fig. 11**

Analysis of proteins characteristic of a senescence-associated secretory phenotype (SASP) in MCF7-P and MCF7-Res cells. Single agent treatment of either CDK4/6i or DEG-77 resulted in a significant increase in the secretion of proteins associated with the SASP in MCF7-P cells but not in MCF7-Res cells. A combination of both agents led to pronounced SASP secretion in MCF7-P cells. In contrast, MCF7-Res cells exhibited a minimal increase in SASP protein secretion with either single agent, and a modest increase in select chemokines (but not growth factors) in response to combination therapy. The samples used in this study were derived from Figure 8, Panel H and I.

## Supplemental Fig. 12

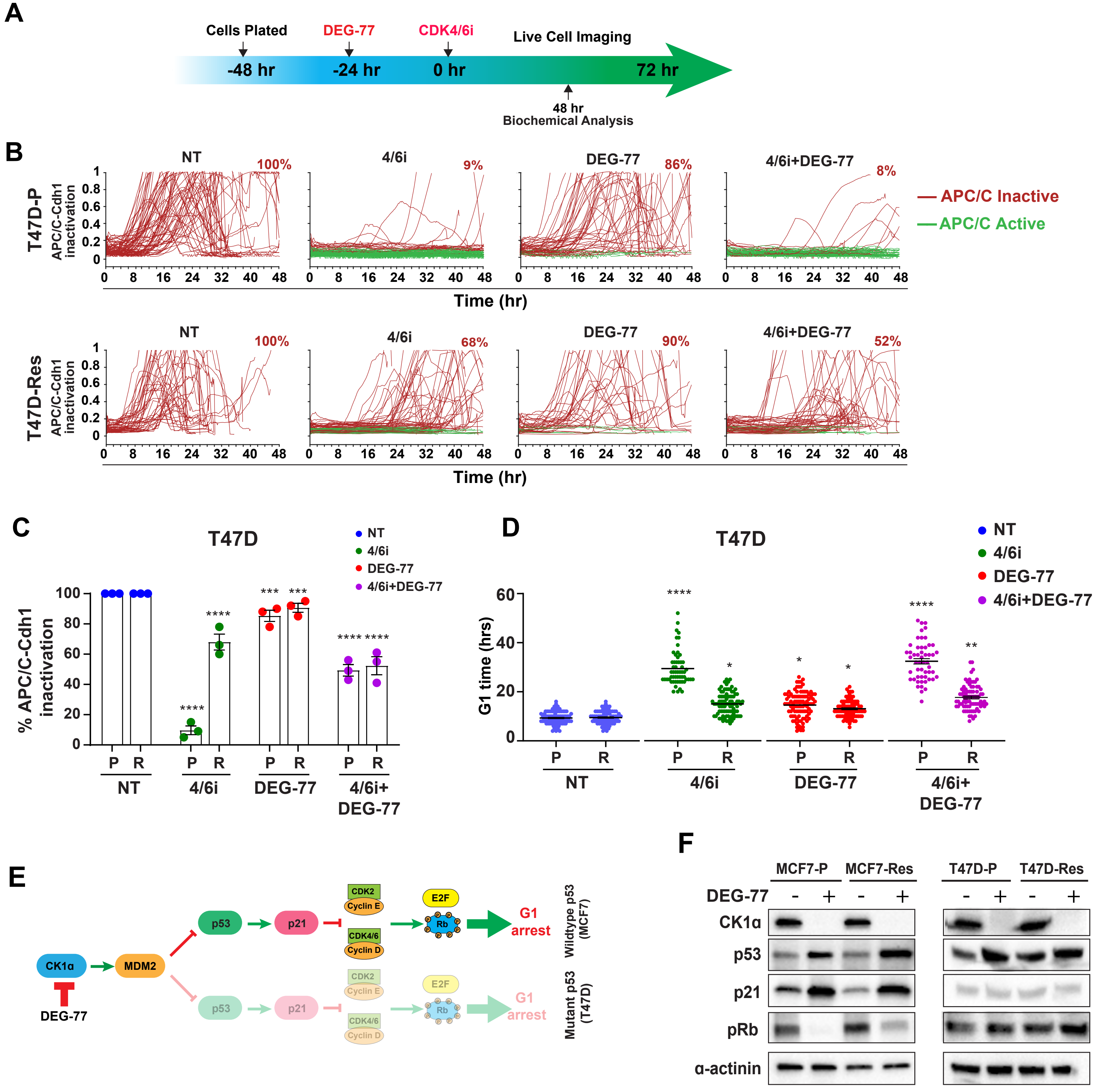

**Supplemental Fig 12**

**(A)** Experimental outline to investigate a role for p53-mediated p21 induction in the efficacy of DEG-77. APC/C-Cdh1 activity was evaluated by live-cell imaging as described above in T47D-P and T47D-Res cells treated with CDK4/6i, DEG-77 or a combination of. **(B-D)** Single cell traces of mCherry-Gem$_{1\text{-}110}$ levels post-mitosis as a surrogate for APC/C-Cdh1 inactivation during G1 to S transition in T47D-P and T47D-Res cells were acquired over 48 h (b). Representative traces from three biological replicate experiments were used to quantify the percentage of cells undergoing APC/C-Cdh1 inactivation under each condition in T47D-P (P) or T47-D-Res cells (R, panel C). Time required for APC/C-Cdh1 inactivation post-mitosis (G1 time) was also derived under each condition in each cell line (panel D). **(E)** DEG-77 mediates efficient CK1α degradation in T47D cells (panel F), yet did not robustly impact the percentage of cells experiencing APC/C-Cdh1 inactivation (panel D) and time in G1 (panel D), compared to MCF7 cells (Figure 8). We hypothesized that this may be due to the mutant p53 status of T47D while MCF7 cells are wild type for p53. CK1α activity stabilizes the p53-MDM2 complex such that the phosphorylated form of MDM2 (and MDMX) has a repressor function on the p53 pathway. In MCF7 cells, inhibition of CK1α activity by DEG-77 results in p53 accumulation thus promoting p21 expression. p21 inhibits cyclin E-CDK2 and cyclin D-CDK4/6 activity thereby contributing to DEG-77 mediated G1 arrest. The mutant p53 status of T47D cells negates the p53-p21 axis in response to DEG-77 treatment. This hypothesis was tested below. **(F)** Western blot analysis highlighting degradation of CK1α, upregulation of p53 and p21, as well as decrease in pRb levels (indicating cell cycle arrest) in MCF7-P and MCF7-Res cells post-DEG-77 treatment. In contrast, T47D cells demonstrate elevated p53 levels in untreated cells (indicative of mutant p53 status) with no significant change to p53 or p21 levels in response to DEG-77, despite robust CK1α degradation.

Drug concentrations CDK4/6i (ribociclib) = 200 nM, DEG-77 = 1 µM. Error bars represent mean ±SEM. *, $P < 0.05$; **, $P < 0.01$; ***, $P < 0.001$ and ****, $P < 0.0001$. Statistical significance were calculated by comparing individual or combination drug treated parental or resistant cell type with their respective NT condition.

## Supplemental Fig. 13

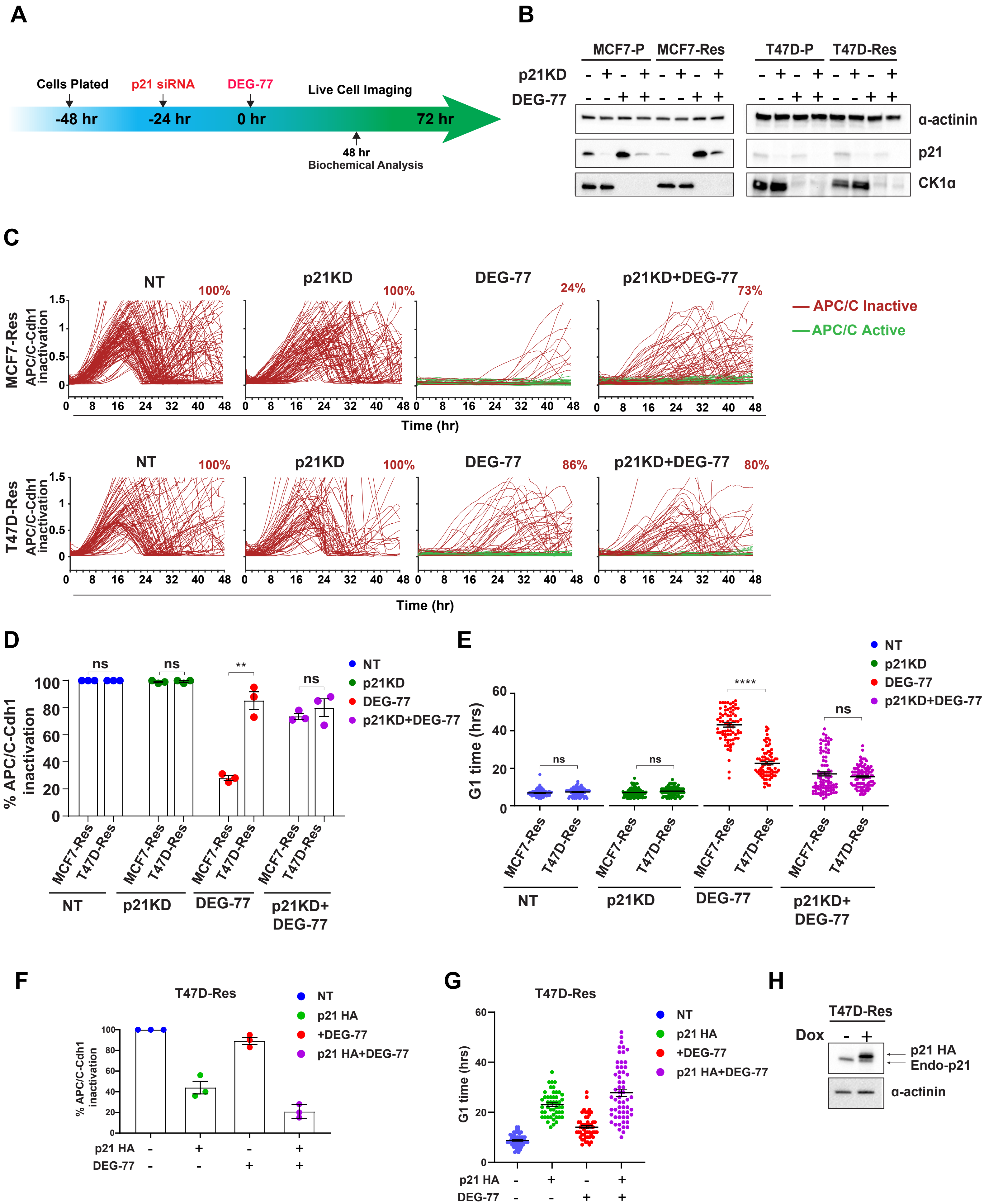

**Supplemental Fig. 13**

**(A)** To evaluate the contribution of p21 in the delayed G1 to S transition upon CK1α degradation, we conducted siRNA mediated knockdown studies in the presence and absence of DEG-77 as indicated. **(B)** Western blot analysis demonstrating CK1α degradation and p21 induction in MCF7-P and MCF7-Res cells. Efficient siRNA-mediated knockdown of p21 in DEG-77 treated MCF7 was accomplished. p21 levels were low in untreated cells and were not induced in response to CK1α degradation. **(C-E)** Single cell traces of mCherry-$Gem_{1-110}$ reporter levels (APC/C-Cdh1 activity) in control (NT), p21 siRNA treated (p21KD), DEG-77 treated as well as combined treatment (p21KD+DEG-77) of MCF7-Res and T47D-Res cells. Representative traces from three biological replicate experiments were quantitatively analyzed to derive the percentage of cells experiencing APC/C-Cdh1 inactivation (panel D) and time in G1 for >200 cells (panel E). **(F)** The bar graph shows the percentage of T47D-Res PIP-FUCCI cells that inactivated APC/C-Cdh1 under the specified conditions: untreated control (CT), doxycycline-induced HA tagged p21 overexpression alone (p21 HA), DEG-77 treatment alone (DEG-77), and the combination of p21 overexpression with DEG-77 (p21 HA + DEG-77).
**(G)** The bar graph shows the duration of the G1 phase (hours from mitosis to mCherry-Geminin accumulation) for cells that completed the G1/S transition under the same conditions as in F.

# Supplementary Fig. 14

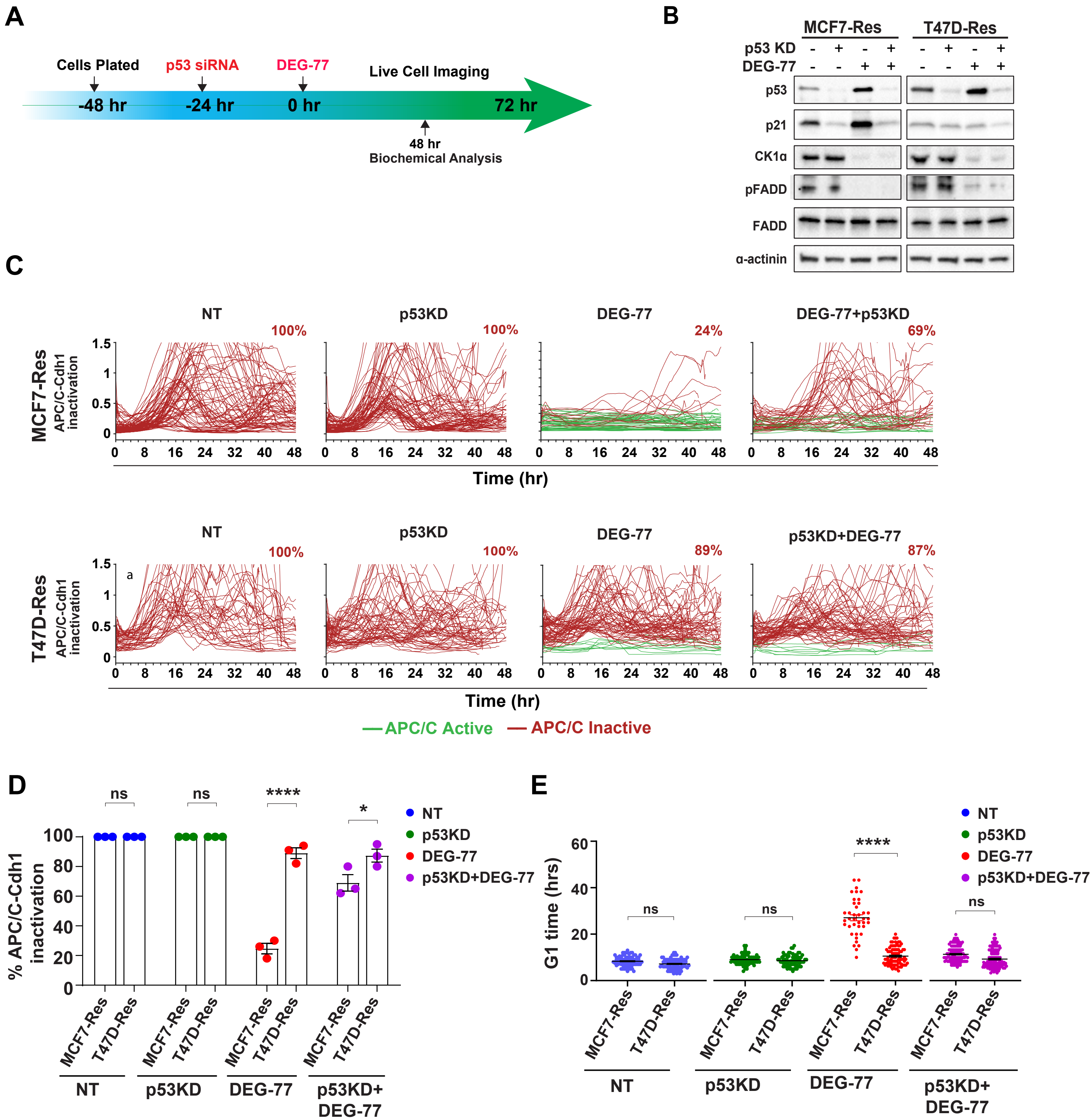

**Supplemental Fig. 14**

**(A)** Schematic of the experimental design for TP53 knockdown in MCF7-Resistant (p53 WT) and T47D-Resistant (p53 mutant) cells treated with DEG-77 and/or CDK4/6 inhibitor (4/6i). **(B)** Immunoblot analysis confirming efficient TP53 knockdown, loss of p21 induction by DEG-77, degradation of CK1α, reduction of phospho-FADD (Ser194), and stable total FADD levels. **(C)** Representative single-cell traces of mCherry-Geminin accumulation (reporting APC/C-Cdh1 inactivation) for the indicated conditions in MCF7-Res and T47D-Res cells. **(D)** Quantification of the percentage of cells that underwent APC/C-Cdh1 inactivation (G1/S transition) during the imaging period. Conditions: Control (CT), TP53 knockdown (p53 KD), DEG-77, and DEG-77 + p53 KD. **(E)** Quantification of G1 residence time (hours from mitosis to mCherry-Geminin accumulation) for cells that entered S phase under the conditions in (D).

For D and E, one-way ANOVA with Sidak correction was used for indicated comparisons. Data are mean ± SEM in 3 or more biological replicates.

## Supplemental Fig. 15

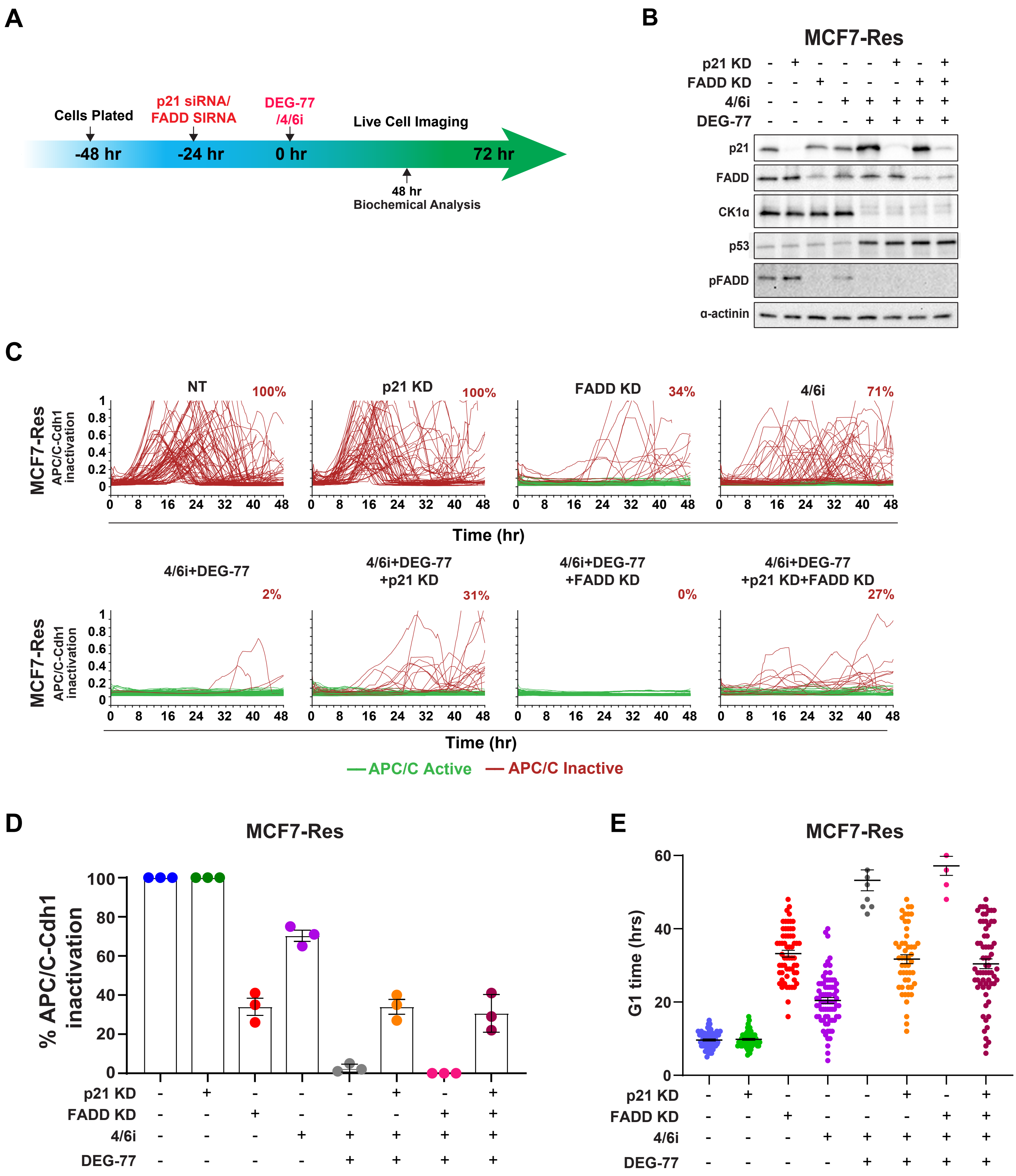

**Supplemental Fig. 15**

**(A)** Schematic of the combinatorial knockdown and treatment experiment in MCF7-Resistant cells. **(B)** Immunoblot analysis confirming efficient knockdown of p21 and FADD, and the molecular effects of DEG-77 treatment (degradation of CK1α, reduction of p-FADD, stabilization of p53). **(C)** Representative single-cell traces of mCherry-Geminin accumulation, reporting APC/C-Cdh1 inactivation and G1/S transition, for the indicated conditions. **(D-E)** Quantification of the percentage of cells that underwent APC/C-Cdh1 inactivation (D) and quantification of G1 time during the imaging period for each condition (E).

Drug concentrations: 4/6i (ribociclib), 200 nM; DEG-77, 1 μM; siRNA, 20 nM. For D and E, one-way ANOVA with Sidak multiple-comparisons correction was used for prespecified comparisons. Data are mean ± SEM from ≥3 biological replicates; >200 cells were analyzed per condition. *$P < 0.05$; **$P < 0.01$; ***$P < 0.001$; ****$P < 0.0001$.

# Supplimental Fig 16

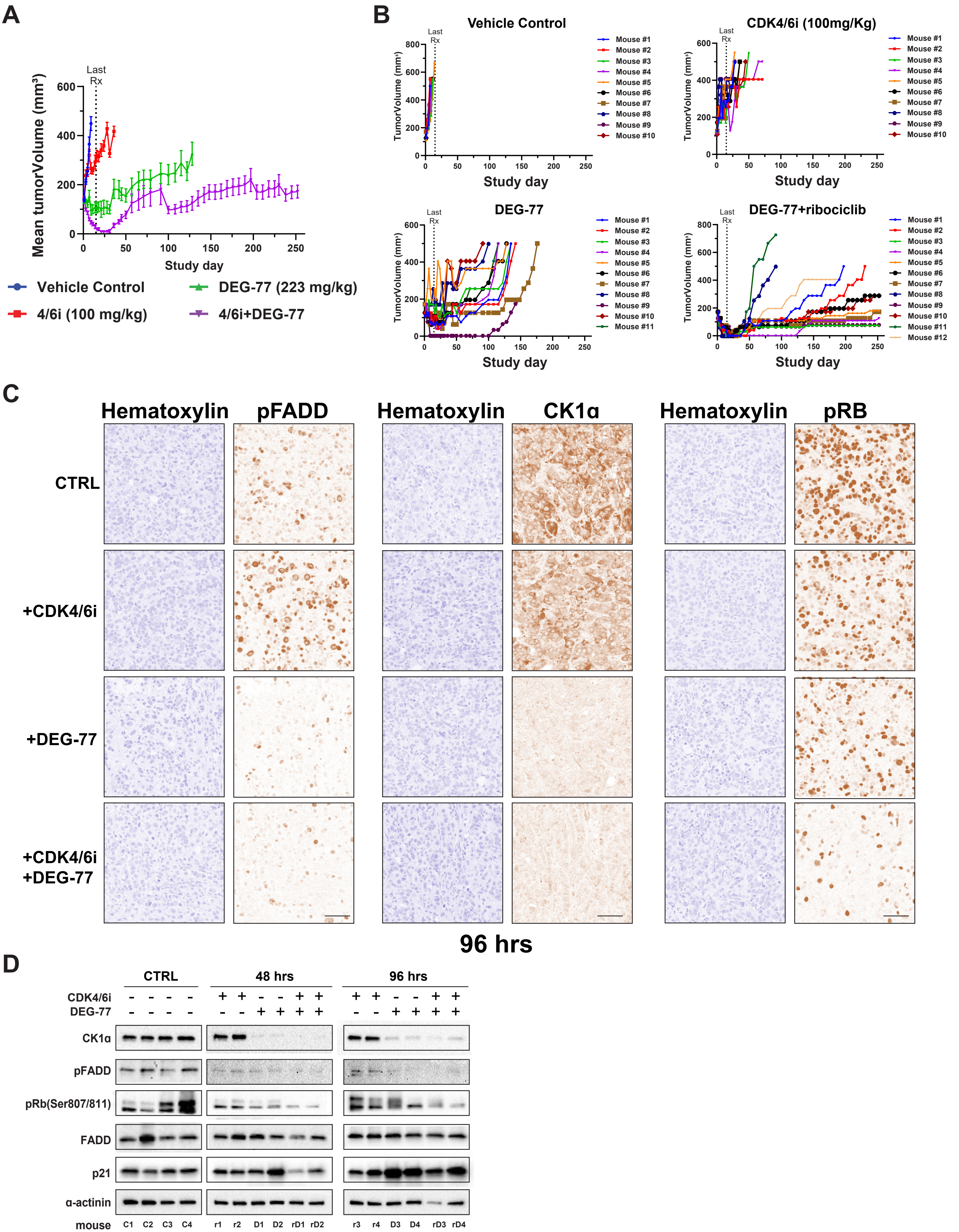

**Supplemental Fig. 16**

**(A)** Mean tumor volume (± SEM) for MCF7-Res xenografts treated with vehicle, ribociclib, DEG-77, or the combination, plotted from treatment start to the study endpoint (tumor volume ≥ 500 $mm^3$). The dashed line indicates the end of the 14-day treatment period. The combination of DEG-77 and ribociclib induced profound tumor regressions followed by significantly delayed regrowth compared to all other groups **(B)** Individual mice tumor volume for MCF7-Res xenografts treated with vehicle, ribociclib, DEG-77, or the combination, plotted from treatment start to the study endpoint (tumor volume ≥ 500 $mm^3$). The dashed line indicates the end of the 14-day treatment period. **C)** Immunohistochemistry analysis of resected tumors from mice implanted with MCF7-Res cells, 96 hrs post treatment with CDK4/6i, DEG-77 or their combination. Sections were stained with Hematoxylin counterstain, or using antibodies against pFADD, CK1$\alpha$ or pRB. Treatment of tumor bearing animals with a CDK4/6i resulted in a modest decrease in pRb levels, while treatment with DEG-77 resulted in a significant decrease in CK1$\alpha$, with a corresponding decrease in pFADD levels, and pRB staining. The combination of the two agents resulted in a dramatic decrease in pRb levels, CK1$\alpha$ and pRB levels as well (panel C). *Note:* The untreated control images are the same as those shown in Fig. 9F, as they served as the control for both the 48-hour (Fig. 9F) and 96-hour (this panel) time points. Scale bar – 50 μm **(D)** Western blot analysis of proteins extracted from resected tumor tissue. Four control tumor samples (CTRL) as well as paired tumor samples for each treatment condition at 48 h and 96 h post-treatment demonstrate that a reduction in CK1α levels correlate with a decrease in pFADD, and pRb levels in response to DEG-77 or combined DEG-77 and CDK4/6i treatment. As seen above by immunohistopathology, these results confirm that pRB levels were lowest when using combination therapy compared to either agent alone.

## Supplemental Methods

### Constructs

The constructs used in the study included:

- pLenti-PGK-Neo-PIP-FUCCI, a gift from Jean Cook (Addgene plasmid #118616; http://n2t.net/addgene:118616; RRID: Addgene_118616) (2).

- pLC-Flag-CSNK1A1 WT-Puro, a gift from Eva Gottwein (Addgene plasmid #123319; http://n2t.net/addgene:123319; RRID: Addgene_123319) and pLC-Flag-CSNK1A1 G40N-puro, a gift from Eva Gottwein (Addgene plasmid #123320; http://n2t.net/addgene:123320; RRID: Addgene_123320) (47).

-pTRIPZ-p21-HA was a gift from Michele Pagano (Addgene plasmid # 215110 ; http://n2t.net/addgene:215110 ; RRID:Addgene_215110) (59).

-pTRIPZ-Halo-FADD, as described in our previous studies (89).

### Lentiviral Constructs and Transduction

Stable cells expressing PIP-FUCCI were generated via lentivirus transduction. Lentiviruses were prepared by transfecting HEK293FT cells with pLenti constructs (6 µg), pMD2.G (0.8 µg), and pSPAX2 (4 µg) constructs (pMD2.G and pSPAX2 were a gift from Didier Trono, Addgene plasmid #12259 and #12260). The medium was changed 24 h post-transfection, and lentiviruses were harvested 48 h post-transfection. MCF7 and T47D cells were infected with 1 ml lentivirus particles mixed with 1 ml culture medium in six-well plates overnight. The infection medium was replaced with complete medium containing 500 µg/ml neomycin (G418) the next day. Total cell lysates were subjected to Western blot analysis to confirm protein expression. Single cell clones were obtained by serial dilution in 96-well plates, followed by outgrowth into larger dishes.

### siRNA Transfection

MCF7 and T47D cells were transfected with siRNAs using Lipofectamine RNAiMAX reagent (Invitrogen, 13778500) as per the manufacturer's instructions. SiRNAs were procured from Dharmacon SiGenome project: FADD siRNA #1 (D-003800-01-0005; targeting ORF of FADD), FADD siRNA #2 (D-003800-19-0005; targeting 3'UTR of FADD), Control siRNA (non-targeting #1) at a final concentration of 20 nM. The accuracy and validity of the knockdown were confirmed by using both siRNAs in key experiments. p21 knockdown was performed using the following: L-003471-00-0010, ON-TARGETplus Human CDKN1A (1026) siRNA – SMARTpool at a final concentration of 20 nM. TP53 was knocked down using following siRNA: ON-TARGETplus Human TP53(7157) siRNA – SMARTpool (L-003329-00-0005)

### Cell Lines

Human embryonic kidney (HEK) 293FT cells (Thermo Scientific) were employed for all lentivirus production experiments. MCF7 (HTB-22) and T47D (HTB-133) cells were purchased from

ATCC. MCF7 cells were cultured in Dulbecco's Modified Eagle's Medium (DMEM) (GIBCO) and T47D cells were cultured in RPMI-1640 (GIBCO), supplemented with 10% fetal bovine serum (FBS) and antibiotics (penicillin-streptomycin). All cell lines were maintained at 37°C in a humidified atmosphere of 5% $CO_2$. Experiments were conducted using early passage cells, which were passaged no more than 15 times. Mycoplasma contamination was routinely checked using the MycoAlert Mycoplasma Detection Kit (Lonza, LT07-118).

Resistant MCF7 and T47D cells were generated in our laboratory by chronic CDK4/6i ribociclib selection and subsequently tested for cross-resistance to palbociclib and abemaciclib. This strategy is consistent with published approaches using MCF7-derived CDK4/6i-resistant models. In our paired models, MCF7-Res cells showed 12–37-fold higher IC50 values than MCF7-P cells, depending on the CDK4/6 inhibitor tested (Fig. 2B).

**Cell Lysis and Immunoblotting**

Cells cultured in 100 mm tissue culture dishes were washed with PBS and subsequently scraped into a lysis buffer containing 50 mM HEPES (pH 7.4), 10 mM EDTA, 150 mM NaCl, 1% NP-40, 0.5% sodium deoxycholate, and 0.1% SDS, with freshly added protease and phosphatase inhibitor cocktails (Roche). The cell lysate was centrifuged at 16,800 g for 10 minutes at 4°C, and the supernatant was collected.

For immunoblotting, the cell lysates were boiled in 2X Laemmli buffer, and proteins were separated by SDS-PAGE using 4–20% Mini-PROTEAN® TGX™ Precast Gels (Bio-Rad, #4561093EDU). Following separation, the proteins were transferred onto PVDF membranes using the Bio-Rad Trans-Blot® Turbo™ Transfer System (Bio-Rad, #1704150EDU). The membranes were then probed with the respective antibodies, and immunocomplexes were detected using the Bio-Rad Clarity Western ECL Substrate (Bio-Rad, #1705061).

**Immunocytochemistry**

Immunocytochemistry assays were performed using cells fixed on round cover glass (#1.5 thickness, 10 mm; Thomas Scientific, 1217N78). MCF7 cells were grown on coverslips, fixed for 10 min with 3.7% paraformaldehyde, and quenched with 10 mM ammonium chloride. Cells were permeabilized with 0.1% Triton X-100 in PBS for 10 min, washed with PBS, and blocked twice: first in PBS with 2.5% goat serum and 0.2% Tween 20 for 10 min, followed by PBS with 0.4% fish skin gelatin and 0.2% Tween 20 for another 10 min. Cells were incubated with primary antibodies for 1 h at room temperature, washed with PBS containing 0.2% Tween 20, and incubated with Alexa Fluor 594 or 647 secondary antibodies for 45 min. The coverslips were washed again and mounted on glass slides using ProLong Diamond Antifade Mountant (Thermo Fisher Scientific, P36965).

**Senescence-Associated β-Galactosidase Staining**

Senescence-associated β-galactosidase (SA-β-gal) staining was performed with MCF7-P and MCF7-Res cells using the Senescence β-Galactosidase Staining Kit (Cell Signaling Technology, Cat. #9860) following the manufacturer's guidelines. MCF7 cells were cultured in 24-well plates and stained at days 7, and 15. Briefly, cells were fixed and incubated with the provided β-galactosidase staining solution. Following incubation, the cells were rinsed, and images were

captured using a Nikon Ti2 microscope equipped with a 10X objective. The presence of blue-stained cells indicated β-galactosidase activity, which is a marker of cellular senescence.

**Quantifying Cytokines and Growth Factors**

Conditioned media from the indicated cells were collected, after which the cells were trypsinized and counted using a cytometer (Bio-Rad TC20 automated cell counter). The media samples were normalized based on cell number by diluting with culture media. Aliquots (50 μL) of the conditioned media were then used for quantification using the Bio-Plex 200 system, following the manufacturer's protocol (Bio-Rad, CA, USA). The chemokines and cytokines detected include: CCL-2 (pg/ml), CCL-5 (pg/ml), CXCL-1 (pg/ml), CXCL-8 (pg/ml), CXCL-10 (pg/ml), PDGF-bb (pg/ml), G-CSF (pg/ml), VEGF (pg/ml), TGF-β (pg/ml).

**Live Cell Imaging**

MCF7 and T47D cells were plated 16 h prior to siRNA transfection in either CELLVIS 12-well #1.5 glass bottom dishes (CELLVIS, P121.5HN) or 8-well multichamber #1.5 glass bottom slides (LABTKII, 155409). Cells were plated at sub-confluent densities to maintain appropriate confluence until the end of the imaging period. Live cell imaging was conducted 24 h post-knockdown in phenol red-free DMEM medium supplemented with 10% FBS and antibiotics. Cells were imaged in a humidified chamber maintained at 37°C and 5% $CO_2$. Images were acquired every 10 min in GFP and mCherry channels using a Zeiss LSM800 Axio Observer.Z1/7 confocal microscope with a Plan-Apochromat 10X/0.45 M27 objective. Exposure time was kept under 100 ms per timepoint to minimize photobleaching and phototoxicity. Images were processed using custom ImageJ macro and MATLAB scripts.

**Image Processing**

Images acquired during live cell imaging were preprocessed to remove background using a Gaussian filter in ImageJ. In the absence of a nuclear channel, the maximum between mVenus and mCherry was used to track cells using TrackMate. Track information and intensities of each channel were provided to custom MATLAB scripts to produce intensity vs time graphs. Representative data from at least three independent experiments, each involving a minimum of 100 cells, are shown. ImageJ macro and MATLAB scripts along with instructions have been uploaded to GitHub.

**Cell Growth Analysis**

Cell growth was assessed using the NucSpot Live 650 (#40082, Biotium) nuclear staining dye to monitor proliferation over 5 days post-treatment. MCF7-P and MCF7-Res cells, including variants expressing empty vector (EV), CK1α-WT, or CK1α-G40N, were cultured in appropriate growth media and treated with varying concentrations of DEG-77 or CDK4/6i (ribociclib) according to experimental requirements. Stained cells were incubated for 5 days, with images captured at regular intervals using an automated microscopic imaging system. Microscopic images were analyzed using Nikon GA3 based automated image processing software to quantify cell density, indicated by nuclear staining. This quantification served as a measure of cell proliferation, with results expressed as fold growth relative to the baseline at the start of treatment.

## *In vivo* xenograft studies

All procedures related to animal handling, care and treatment were performed under an approved protocol (PRO00010288) according to the guidelines set forth by the University of Michigan Institutional Animal Care and Use Committee (IACUC) and following the guidance of the Association for Assessment and Accreditation of Laboratory Animal Care (AAALAC). Animals were housed per institutional guidelines (12-h light–dark cycles in ambient temperatures of 68–75 °F with 30–70% humidity) and were maintained under pathogen-free conditions with food and water provided ad libitum.

Female 6–8-week-old CIEA NOG mice (NOD.Cg-*Prkdcscid Il2rgtm1Sug*/JicTac from Taconic) were used for this experiment. Three days prior to cell implantation, the mice received 17β-estradiol pellets subcutaneously via trocar (0.18 mg/pellet 60-day release, Innovative Research of America catalog # SE-121). Mice were inoculated subcutaneously in the right axilla with 5 x $10^6$ cells suspended in 100 µl of a 1:1 ratio of serum-free medium to Matrigel. Upon reaching a mean tumor volume of ~100 to 150 $mm^3$, mice were randomized into treatment arms before initiation of treatment on day 1 of study. Subcutaneous tumor volume and body weights were measured 2–3 times a week. Tumor volumes were calculated by measuring two perpendicular diameters with calipers and using the formula, tumor volume = (length x width2)/2. Mice were treated for 14 days and monitored at least twice weekly until individual mouse tumor burdens reached IACUC-approved limitations (500 $mm^3$, since MCF7 is an ulcer-prone model). The ΔT/ΔC value was used for animal studies, quantitative measure of tumor growth inhibition in our study. This value is calculated as follows- ΔT: The change in tumor volume in the treatment group (initial tumor volume subtracted from the final tumor volume), ΔC: The change in tumor volume in the control group over the same period. Quantitative data are presented as the mean ± s.e.m. All animals treated were included in the analyses. Data collection and analysis were not performed blind to the conditions of the experiments. Evaluation of efficacy parameters, including calculations of ΔT/ΔC, increase in lifespan, and objective responses were calculated.

DEG-77 was prepared in 100% DMSO at a concentration of 200nM. Ribociclib was prepared daily as a fine suspension in 0.5% methylcellulose at a concentration of 10 mg/ml. Both compounds were administered according to individual mouse body weight (0.2 ml per 20 g).

For pharmacodynamic studies, when tumors reach a mean tumor volume of ~150 $mm^3$, mice were randomized into treatment arms and treated with the vehicle or test article. At the indicated time points, mice were euthanized, and tumors were removed and divided. One half of the tumor was snap-frozen in liquid nitrogen and stored at −80 °C for western blot analysis. The other half of the tumor was fixed in 10% neutral buffered formalin and paraffin processed and embedded for IHC analysis.

## Patient samples

Tumor biopsies at diagnosis as well as post-recurrence while under CDK4/6i and ET treatment were procured and processed as approved by the University of Michigan Institutional Review Board (IRB00005467). All available paired biopsies meeting inclusion criteria were analyzed. Inclusion required ER+/HER2– breast cancer, treatment with a CDK4/6 inhibitor, and availability

of matched pre-treatment and post-progression biopsy tissue. No paired samples meeting these criteria were excluded.

### Histology and Immunohistochemistry Staining

Histology and immunohistochemistry work were performed by the Unit for Laboratory Animal Medicine Pathology Core at the University of Michigan, Ann Arbor (RRID:SCR_018823)

Paraffin processing and Sectioning:

Briefly, formalin-fixed tissues were processed through graded alcohols and cleared with xylene followed by infiltration with molten paraffin using an automated VIP6 tissue processor (TissueTek, Sakura-Americas, Hayward, CA). Tissues were embedded in paraffin blocks (Histostar Embedding Station, Thermo Scientific, Waltham, MA), sectioned at 4 μm thickness on a rotary microtome (RM2255, Leica Biosystems, Buffalo Grove, IL), mounted on glass slides, and deparaffinized prior to staining by heating to 60°C for 1 hour.

IHC Staining:

Deparaffinized slides underwent heat-induced epitope retrieval (HIER) in a pressure chamber (Decloaking Chamber, Biocare Medical, Pacheco, CA) for approximately 40 minutes at a maximum temperature of 127°C in retrieval solutions described in the Supplemental Table 4. Immunohistochemical staining was performed on an automated stainer (IntelliPATH FLX, Biocare Medical) and included blocking of endogenous peroxidases, primary antibody incubation, and polymer-based detection. Primary antibody dilutions, antigen retrieval solution, detection reagents, and incubation times were as listed below. Diaminobenzidine (DAB) was applied for 5 minutes as the chromogen (IPK5010G80, Biocare Medical) and DAB-enhancer was applied for 1 minute (DAB sparkle, DS830, Biocare Medical). Following immunostaining, samples were counterstained with hematoxylin for 5 minutes, dehydrated and cleared through graded alcohols and xylene, and coverslipped. Negative reagent controls followed the same procedure with naïve serum (Universal Negative, IP498G20, Biocare) in place of primary antibody.

Immunohistochemical Quantification:

All IHC slides were evaluated blinded to sample identity and clinical outcome. For each sample, the staining intensity of adjacent non-tumor cells (e.g., highest staining signal from the slide background, stromal cells or benign epithelium) served as an internal negative control to establish the sample-specific background signal. For quantification, four random, non-necrotic high-power fields (HPFs, 40x) encompassing >4000 cells of viable tumor tissue were scored per sample. Within each HPF, tumor cells were scored based on definitive subcellular staining (nuclear for p-FADD, cytoplasmic/membranous for p-AKT) using the following intensity scale relative to the internal negative control: 0 (negative; signal indistinguishable from or lower than control), 1+ (low), 2+ (moderate), and 3+ (high). The percentage of positive tumor cells (those scoring ≥1+) was recorded per HPF and averaged to generate one value per sample. Total FADD and total AKT staining were quantified using the same methodology.

Supplemental Table 1- Clinical characteristics and treatment history for the 11 ER+/HER2– breast cancer cases

| Patient's study number | Receptor status | Endocrine therapy used before CDK4/6i resistance | CDK4/6i used | CDK4/6i treatment duration in months | CDK4/6i treatment line # | Outcome | Time to progression on CDK4/6i from CDK4/6i start date in months | Time to progression on CDK4/6i from Dx in months | Time to death or LTFU from CDK4/6i start in months | Time to death or LTFU from Dx in months | Time from Dx to CDK4/6i start in months |
|---|---|---|---|---|---|---|---|---|---|---|---|
| **R1** | ER+ PR+ HER2- | Tamoxifen Letrozole Leuprolide | Palbociclib | 21.5 | 3 | D | 25.3 | 100.4 | 33.1 | 108.2 | 75.1 |
| **R2** | ER+ PR+ HER2- | Anastrozole Exemestane Fulvestrant | Palbociclib | 16.2 | 3 | A | 15.5 | 133 | 77.9 | 195.5 | 117.5 |
| **R3** | ER+ PR- HER2- | Letrozole | Palbociclib | 25.78 and 11.6 | 2 and 4 | LTFU | 47.9 and 15.0 | 49.1 | 55.6 and 22.7 | 56.7 | 1.2 and 34.1 |
| **R4** | ER+ PR+ HER2- | Letrozole | Palbociclib | 47.2 | 2 | D | 35.4 | 36.4 | 48.10 | 49.0 | 1.0 |
| **R5** | ER+ PR+ HER2- | Exemestane Fulvestrant Goserelin | Palbociclib | 8.5 | 3 | D | 8.5 | 47.4 | 35.9 | 74.8 | 38.9 |
| **R6** | ER+ PR+ HER2- | Letrozole | Ribociclib | 22.8 | 2 | D | 22.0 | 46.6 | 85.3 | 109.9 | 24.6 |
| **R7** | ER+ PR- HER2- | Letrozole | Palbociclib | 20.1 | 1 | D | 19.6 | 20.2 | 19.6 | 20.2 | 0.6 |
| **R8** | ER+ PR+ HER2- | Goserelin Letrozole Leuprolide Fulvestrant | Ribociclib | 21.3 | 2 | D | 21.5 | 26.2 | 23 | 27.7 | 4.7 |
| **NR1** | ER+ PR+ HER2- | Anastrozole Fulvestrant | Palbociclib | 4 | 1 | D | 4.9 | 21.3 | 10.2 | 26.6 | 16.4 |
| **NR2** | ER+ PR+ HER2- | Exemestane Anastrozole Tamoxifen Fulvestrant | Palbociclib | 4.4 | 7 | D | 4.9 | 59.6 | 10.1 | 64.9 | 54.7 |
| **NR3** | ER+ PR+ HER2- | Letrozole | Palbociclib | 3.5 | 4 | D | 4.1 | 130.7 | 14.1 | 140.7 | 126.6 |

Abbreviation: D: Deceased, LTFU: lost to follow up, A: Alive (as of 1/25/2026)

Supplemental Table 2 - Antibodies used for immunoblotting and immunofluorescence

| | Target (Specificity) | Catalog # | Host | Clonality | Vendor | Dilution | Application |
|---|---|---|---|---|---|---|---|
| 1 | Cyclin B1 | sc-245 | Mouse | Monoclonal | Santa Cruz Biotechnology | 1:1000 (WB) | WB |
| 2 | FADD | 2782 | Rabbit | Polyclonal | Cell Signaling Technology | 1:1000 (WB) 1:100 (IF) | WB,IF |
| 3 | Cyclin A2 | 4656 | Mouse | Monoclonal | Cell Signaling Technology | 1:1000 (WB) | WB |
| 4 | α-Actinin | 6487 | Rabbit | Monoclonal | Cell Signaling Technology | 1:1000 (WB) | WB |
| 5 | Phospho-Rb (Ser807/811) | 8516 | Rabbit | Monoclonal | Cell Signaling Technology | 1:1000 (WB) 1:350 (IF) | WB, IF |
| 6 | Phospho-FADD (Ser194) | 2781 | Rabbit | Polyclonal | Cell Signaling Technology | 1:1000 (WB) 1:100 (IF) | WB, IF |
| 7 | Rb | 9309 | Mouse | Monoclonal | Cell Signaling Technology | 1:1000 (WB) 1:350 (IF) | WB, IF |
| 8 | CK1α | 2655 | Rabbit | Monoclonal | Cell Signaling Technology | 1:1000 (WB) | WB |
| 9 | Helios (IKZF2) | 42427 | Rabbit | Monoclonal | Cell Signaling Technology | 1:1000 (WB) | WB |
| 10 | FBXO5 (Emi1) | 37-6600 | Mouse | Monoclonal | Thermo Fisher | 1:1000 (WB) | WB |
| 11 | Phospho-Akt (Ser473) | 4060 | Rabbit | Monoclonal | Cell Signaling Technology | 1:1000 (WB) | WB |
| 12 | Akt (pan) | 4691 | Rabbit | Monoclonal | Cell Signaling Technology | 1:1000 (WB) | WB |
| 13 | PRAS40 | 2610 | Rabbit | Polyclonal | Cell Signaling Technology | 1:1000 (WB) | WB |
| 14 | pPRAS40 (Thr246) | 2997 | Rabbit | Monoclonal | Cell Signaling Technology | 1:1000 (WB) | WB |
| 15 | S6 Ribosomal Protein | 2217 | Rabbit | Monoclonal | Cell Signaling Technology | 1:1000 (WB) | WB |
| 16 | Phospho-S6 (Ser235/236) | 4858 | Rabbit | Monoclonal | Cell Signaling Technology | 1:1000 (WB) | WB |
| 17 | Mouse IgG (H+L), AF405 conjugate | A48258 | Goat | Polyclonal | Thermo Fisher | 1:1000 | IF |
| 18 | Rabbit IgG (H+L), AF647 conjugate | A32787 | Goat | Polyclonal | Thermo Fisher | 1:1000 | IF |
| 19 | Mouse IgG (H+L), HRP conjugate | 715-035-151 | Goat | Polyclonal | Jackson | 1:10000(WB) | WB |
| 20 | Rabbit IgG (H+L), HRP conjugate | 711-035-152 | Goat | Polyclonal | Jackson | 1:10000(WB) | WB |

Supplemental Table 3 - Inhibitors and compounds used in this study

| Reagent | Source | Catalog Number | Working Concentration |
| --- | --- | --- | --- |
| Palbociclib | Med Chem Express | HY-50767 | 200 nM |
| Ribociclib | Med Chem Express | HY-15777 | 200 nM |
| Abemaciclib | Med Chem Express | HY-16297A | 200 nM |
| Fulvestrant | Med Chem Express | HY-13636 | 100 nM |
| BTX161 | Med Chem Express | HY-120084 | 20 µM |
| DEG-77 | WuXi AppTec (custom) | N/A | 1 µM |
| Alpelisib (BYL-719) | Med Chem Express | HY-15244 | 300 nM |

Supplemental Table 4 - Antibodies and conditions for immunohistochemistry

| Primary antibodies[a] | Antibody type | Dilution & incubation time[b] | Antigen retrieval, detection[c,] & DAB Enhancer [d] |
|---|---|---|---|
| phosphorylated FADD,<br>#2781 CST | Rabbit polyclonal | 1:75 (for mouse tissue), 30 min<br>1:50 (for human tissue), 30 min | HIER: CB917<br>Detection: RMR622, 30 min<br>DAB Sparkle DS830, 1 min |
| Fas-associated death domain<br>#2782 CST | Rabbit polyclonal | 1:100 (for mouse tissue), 1 hour<br>1:50 (for human tissue), 1 hour | HIER: DV2004<br>Detection: RMR622, 30 min<br>DAB Sparkle DS830, 1 min<br>Human only |
| Phosphorylated retinoblastoma, clone D20B12, #9661 CST | Rabbit monoclonal | 1:200, 1 hour | HIER: DV2004<br>Detection: RMR622, 30 min |
| casein kinase I,<br>#2655 CST | Rabbit polyclonal | 1:75, 2 hours | HIER: CB917<br>Detection: RMR622, 30 min<br>DAB Sparkle DS830, 1 min |
| AKT, clone C67E7<br>#4691 CST | Rabbit Monoclonal | 1:100 1hr | HIER: DV2004<br>Detection: RMR622, 30 min |
| Phosphorylated AKT<br>clone D9E<br>#4060 CST | Rabbit Monoclonal | 1:50, 2 hour | HIER: CB917<br>Detection: RMR622, 30 min, DAB Sparkle DS830, 1 min |

a. CST: Cell Signaling Technology, Danvers, MA; Santa Cruz: Santa Cruz Biotechnology,

b. All antibody dilutions performed in modified PBS (DaVinci Green, #PD900, Biocare Medical, Carlsbad, CA)

c. HIER: heat-induced epitope retrieval; DV2004: Diva Decloaker, citrate-based pH 6.0, Biocare Medical; CB917: EDTA-based decloaker, pH 8.5, Biocare Medical; RMR622: Rabbit-on-Rodent HRP-polymer, Biocare Medical, MM620: Mouse-on-mouse HRP Polymer, Biocare Medical

d. DAB Enhancer (Dab Sparkle, #DS830, Biocare Medical, Carlsbad, CA)
