# Supplementary material for: Targeting casein kinase 1α-mediated FADD phosphorylation restores sensitivity in select CDK4/6 inhibitor-resistant breast cancer models
Source: J Clin Invest. 2026 Jul 28;136(18):e199483. doi: 10.1172/JCI199483 (PMC13574148; doi:10.1172/JCI199483)

Fig 2 Full unedited blot

Full unedited blot for Fig 2I

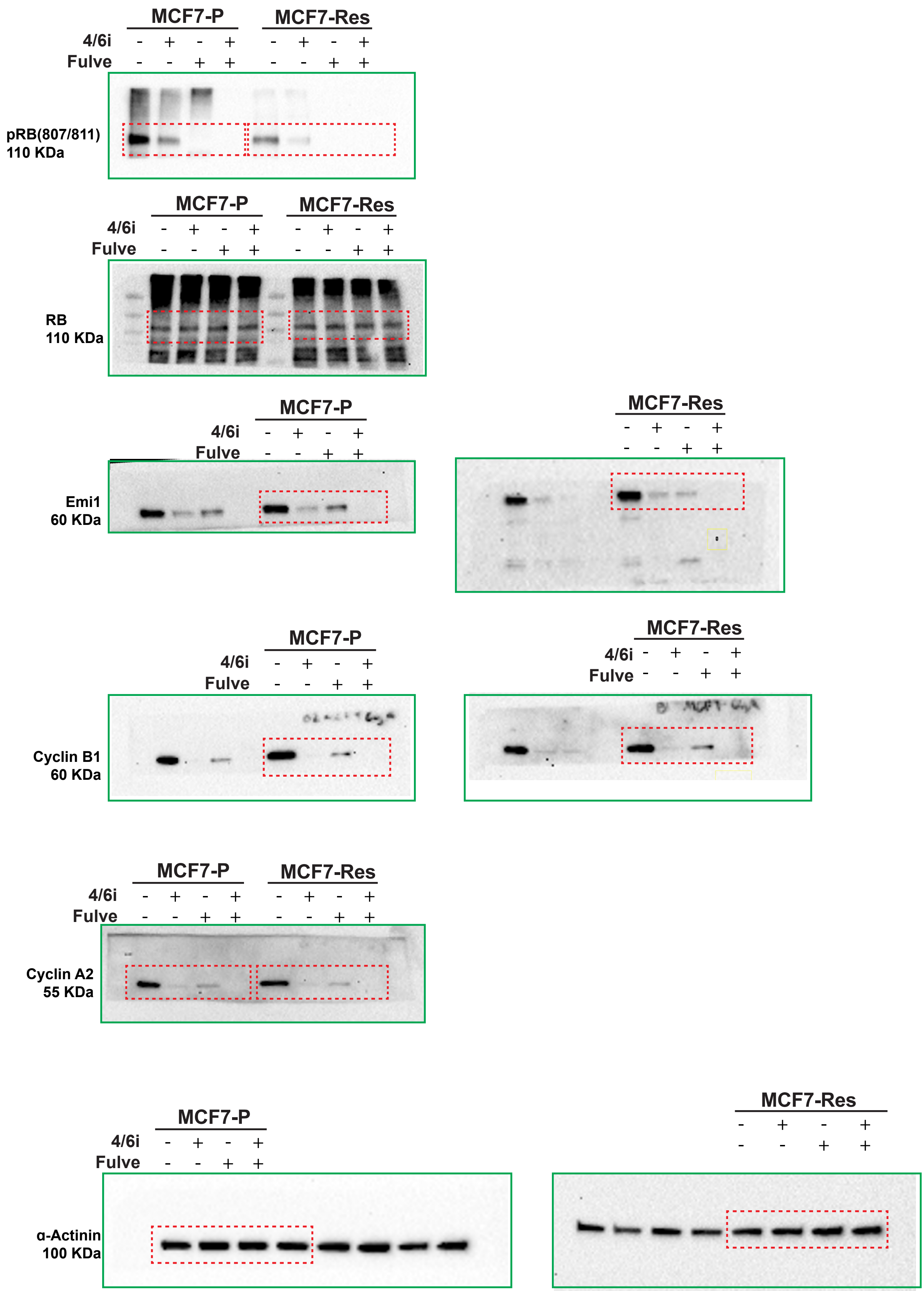

Fig 3 Full unedited blot

Full unedited blot for Fig 3G

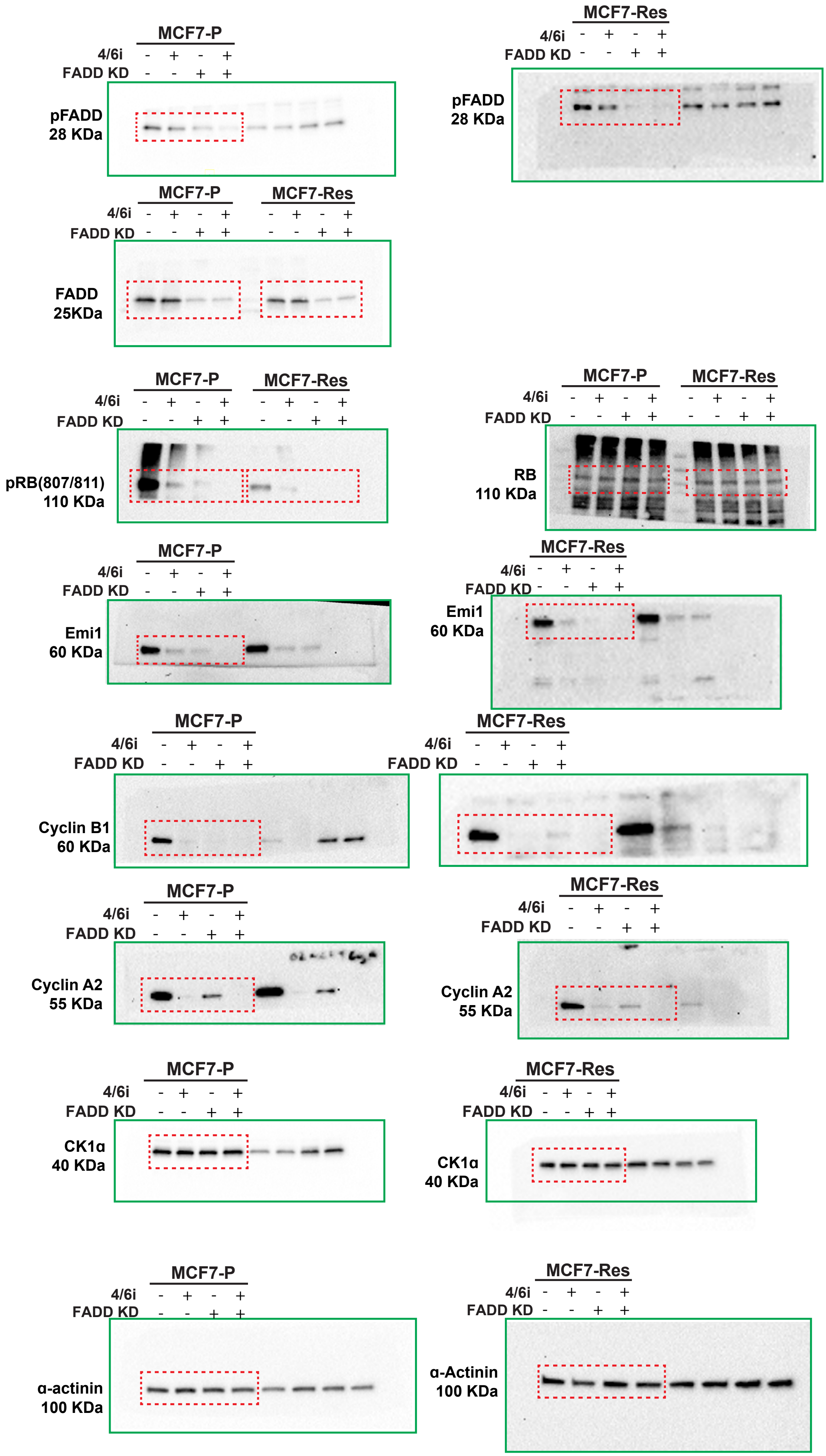

Fig 4 Full unedited blot

Full unedited blot for Fig 4F

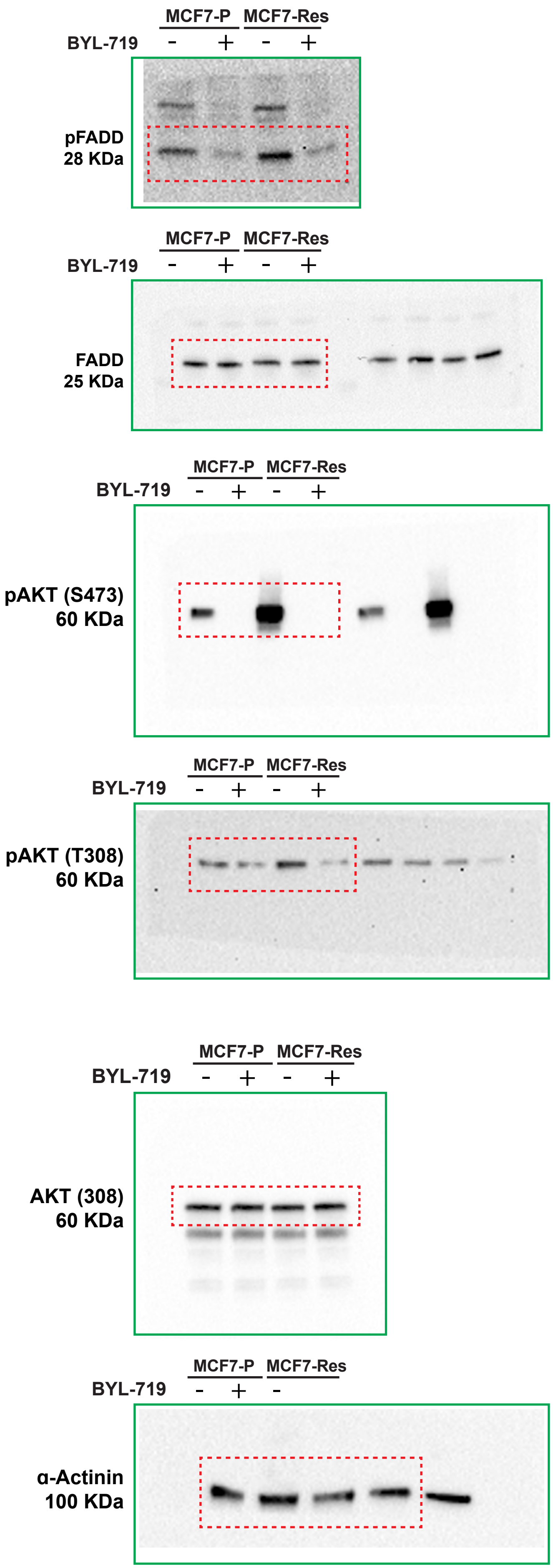

Full unedited blot for Fig 4F

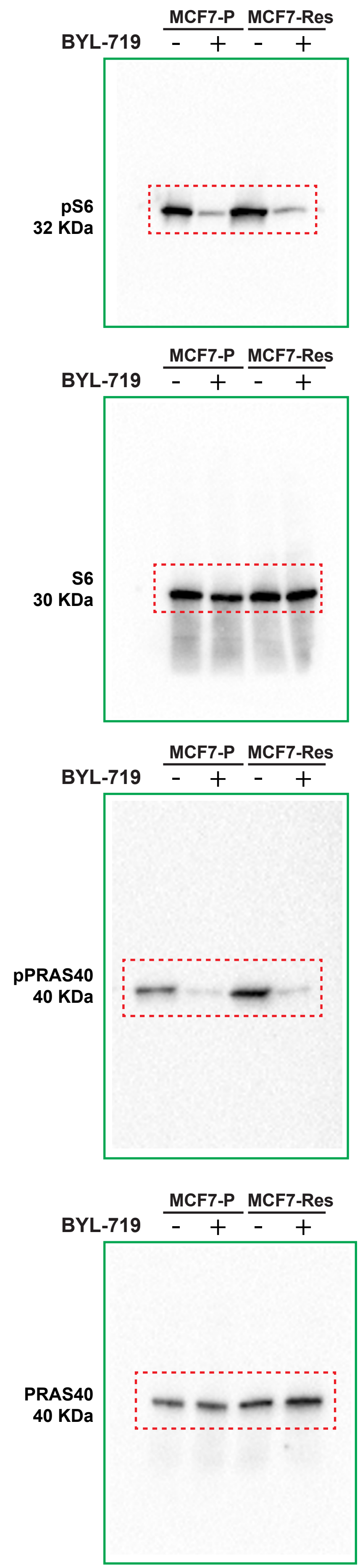

Fig 7 Full unedited blot

Full unedited blot for Fig 7B

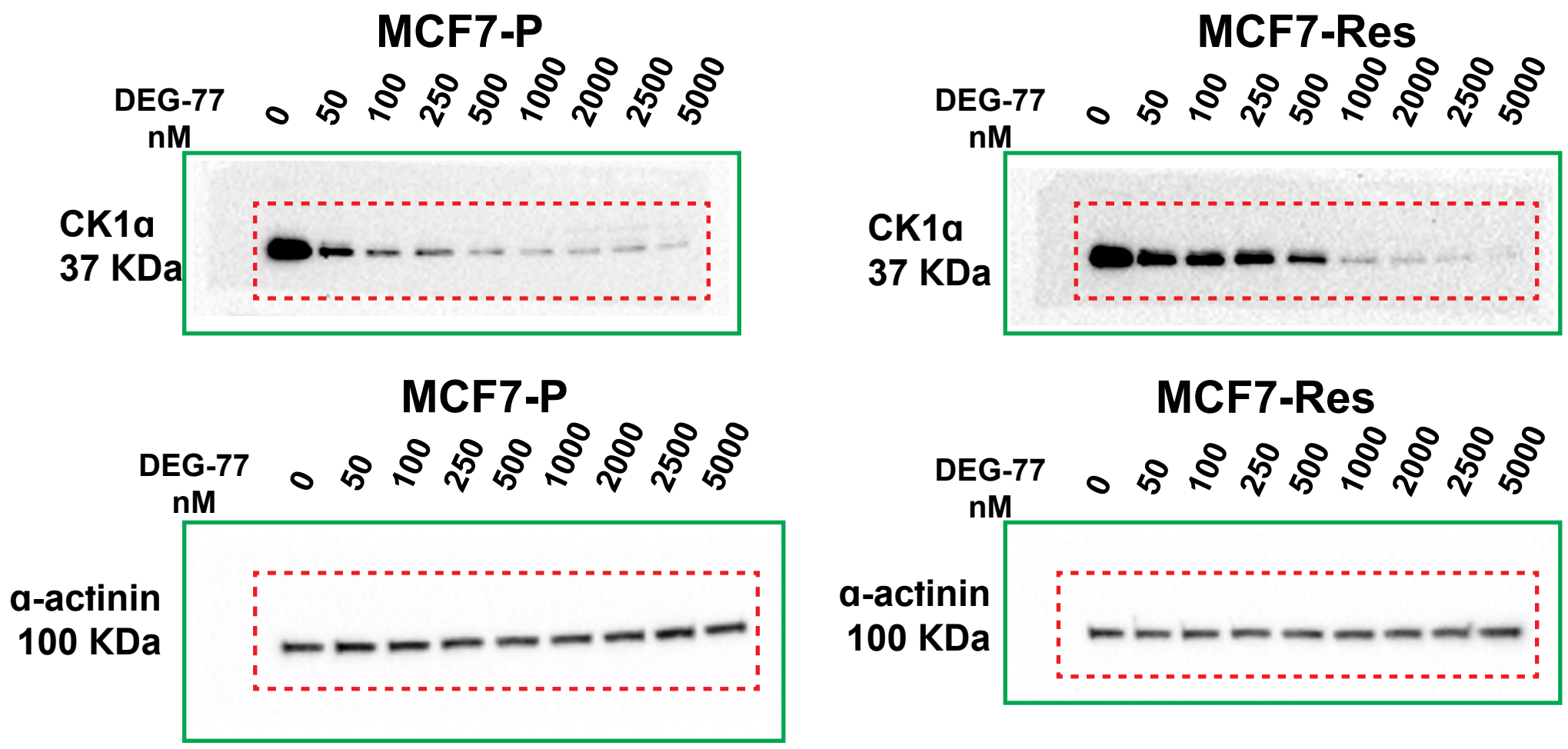

Full unedited blot for Fig 7F

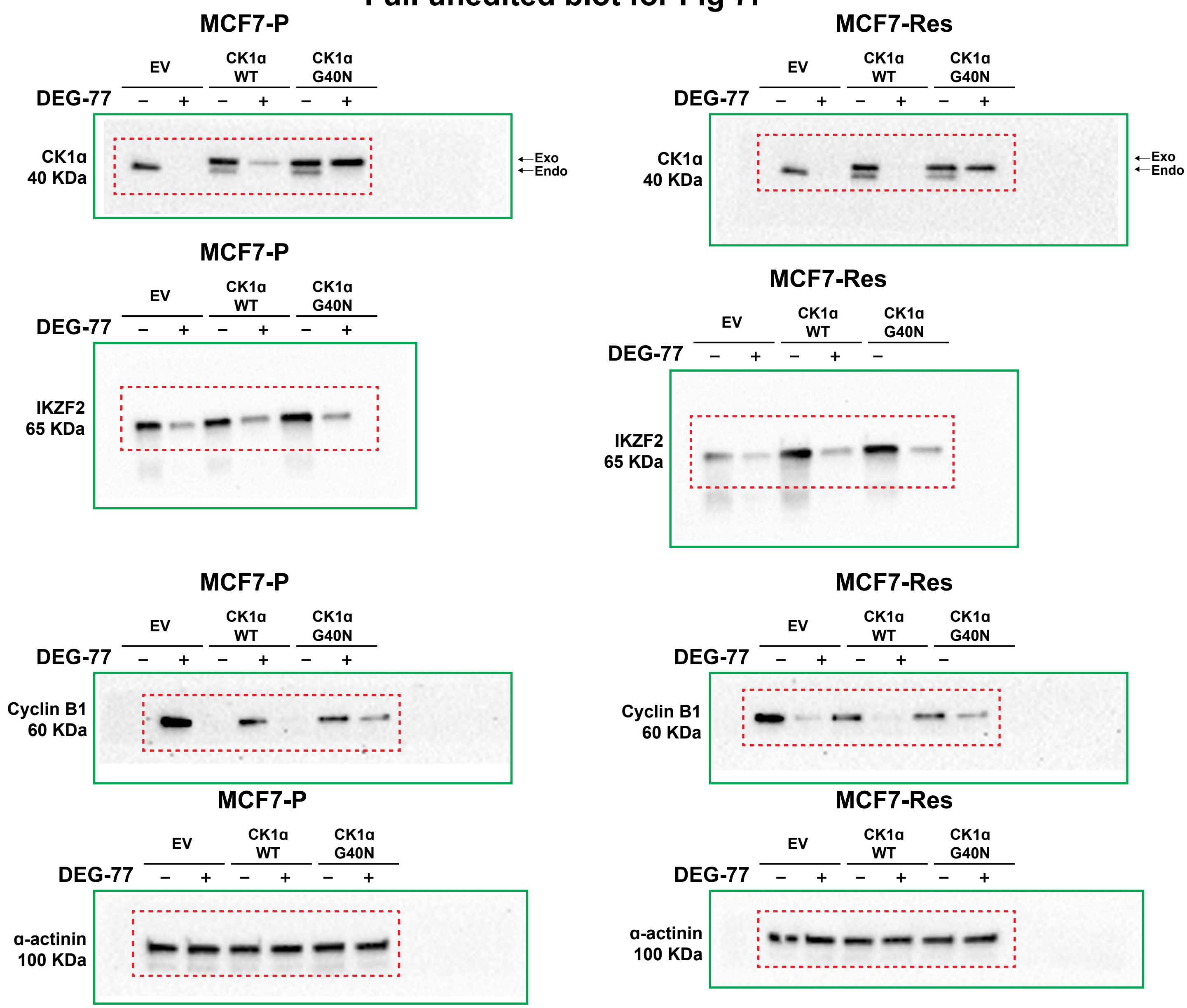

Fig 8 Full unedited blot

Full unedited blot for Fig 8D

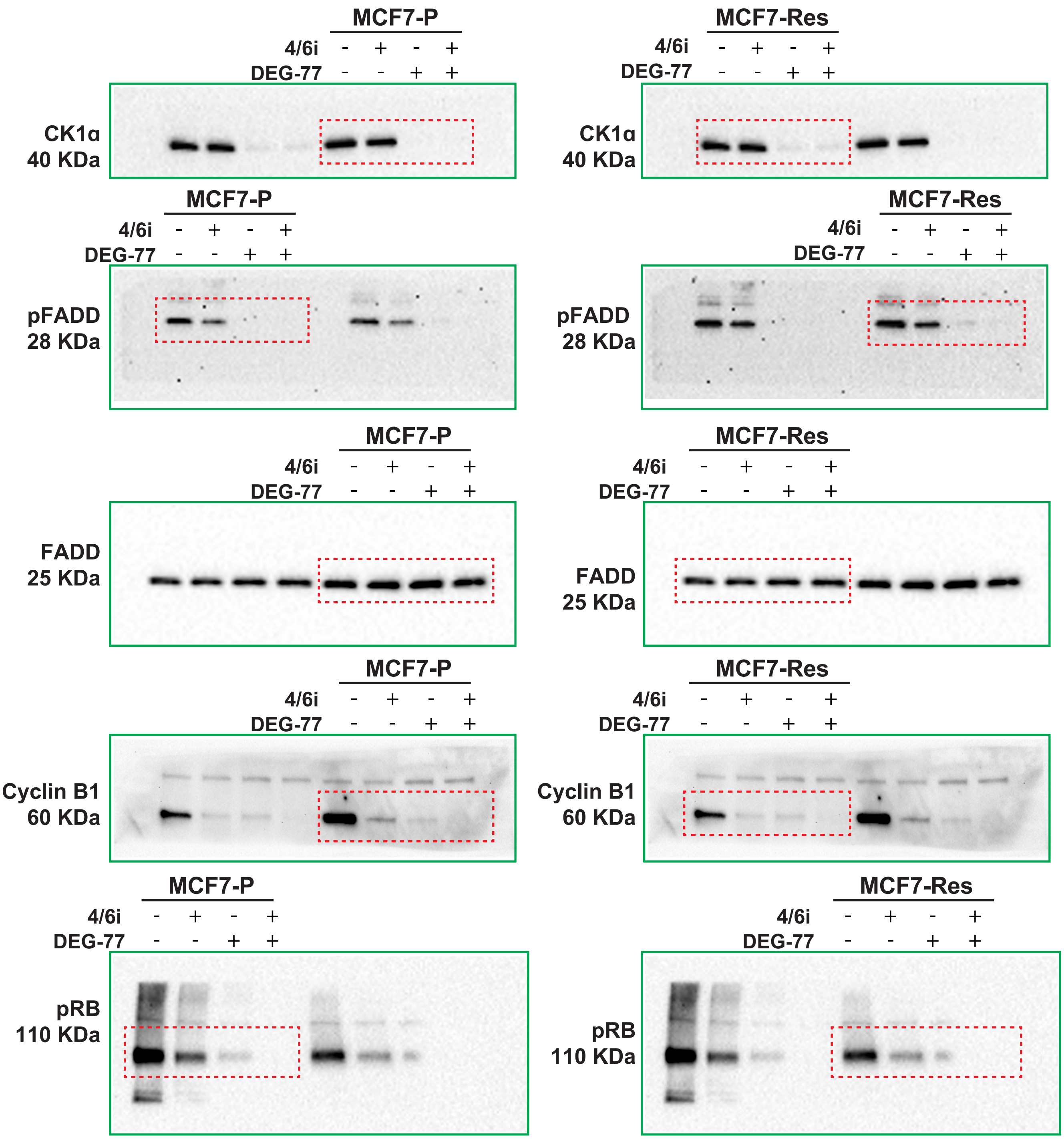

Fig 8 Full unedited blot (cont.)

Full unedited blot for Fig 8D

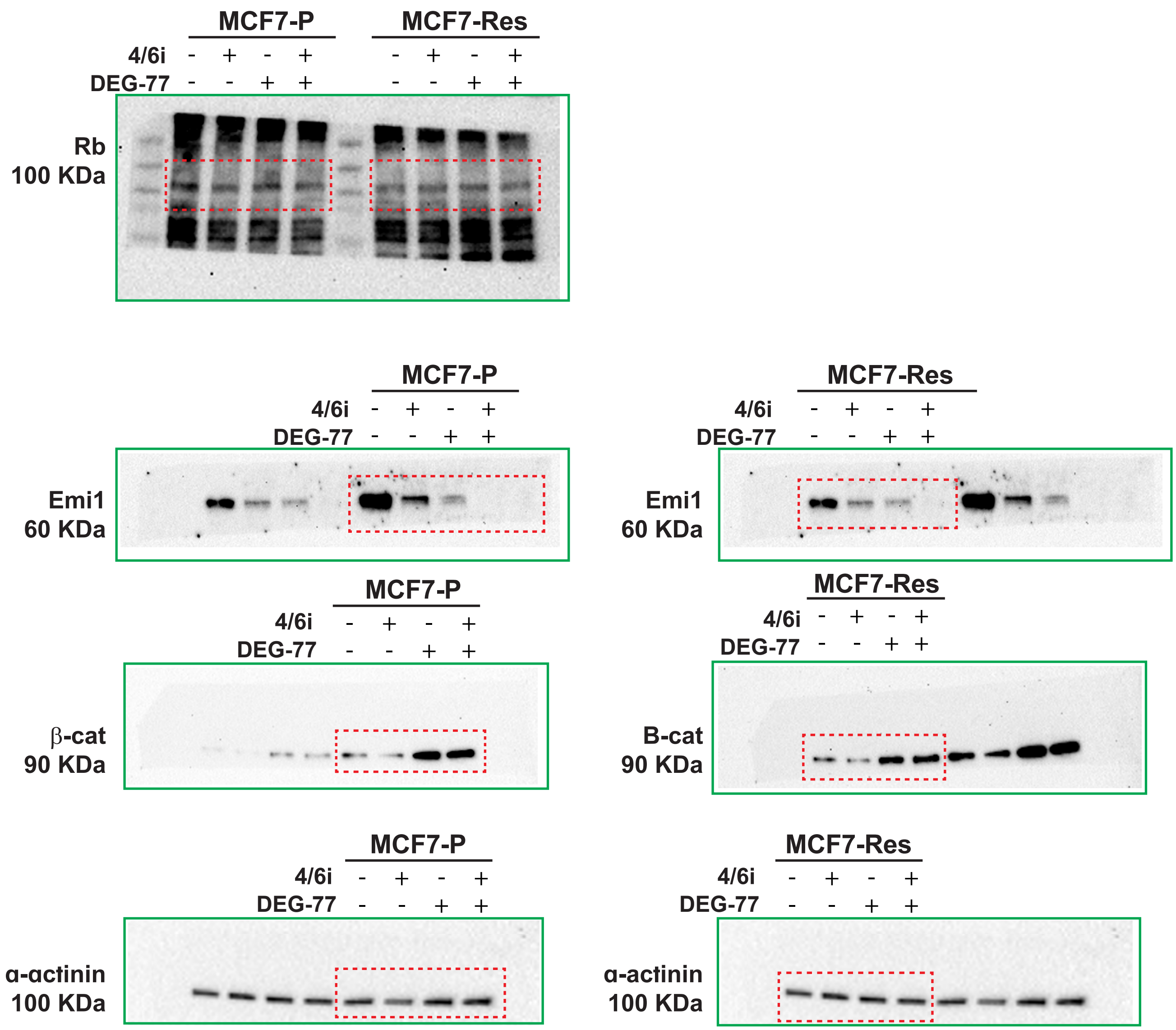

Supplementary Fig 2 Full unedited blot

Full unedited blot for Suppl Fig 2G

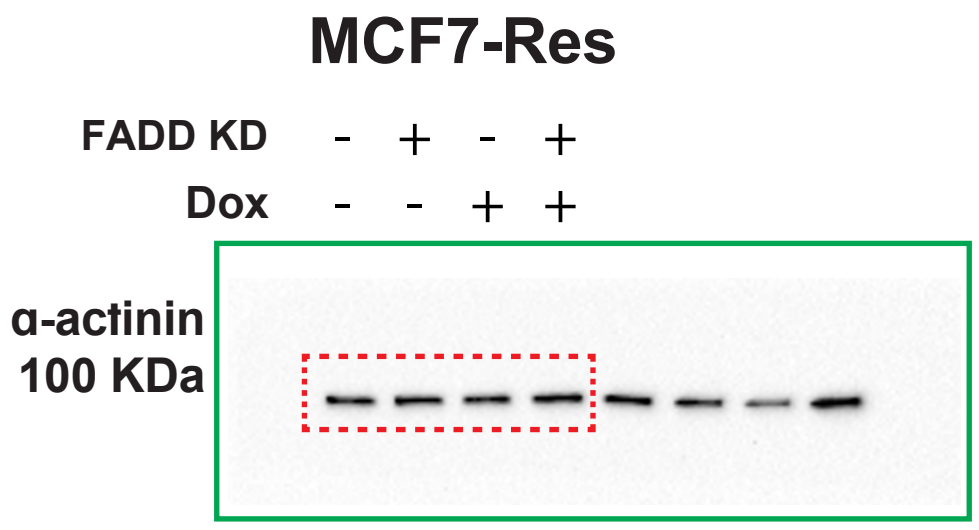

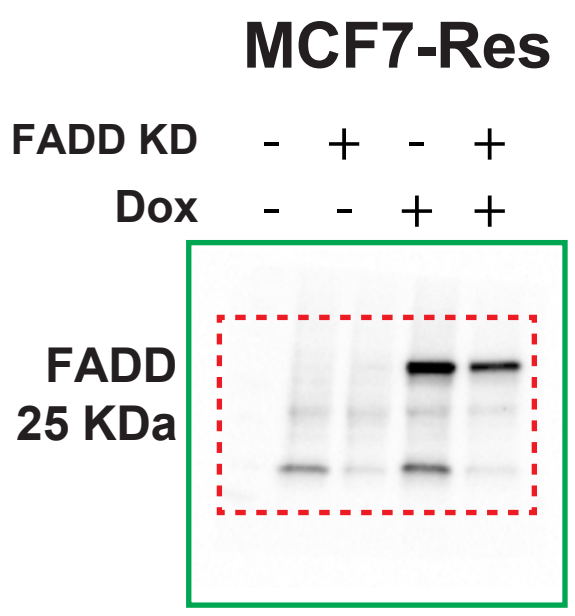

Supplementary Fig 9 Full unedited blot

Full unedited blot for Suppl Fig 9E

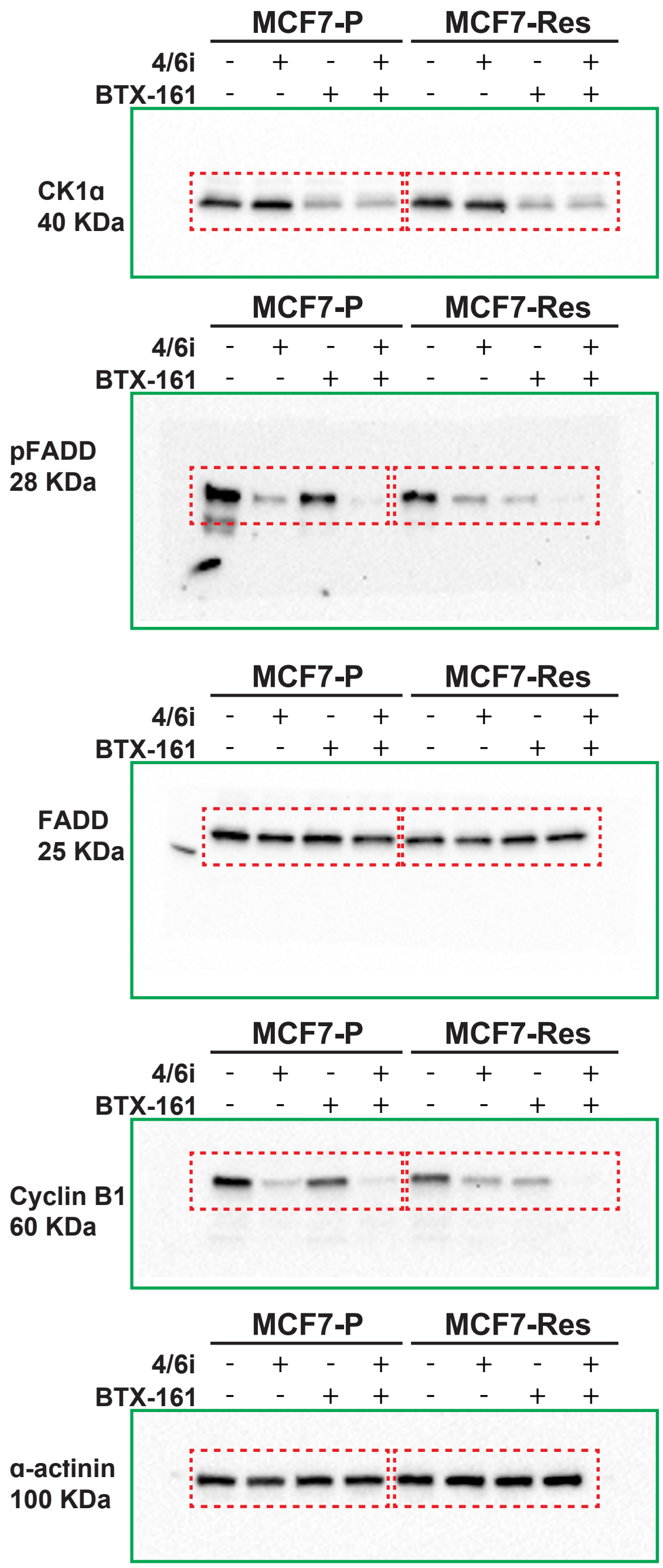

Supplementary Fig 12 Full unedited blot

Full unedited blot for Suppl Fig 12F

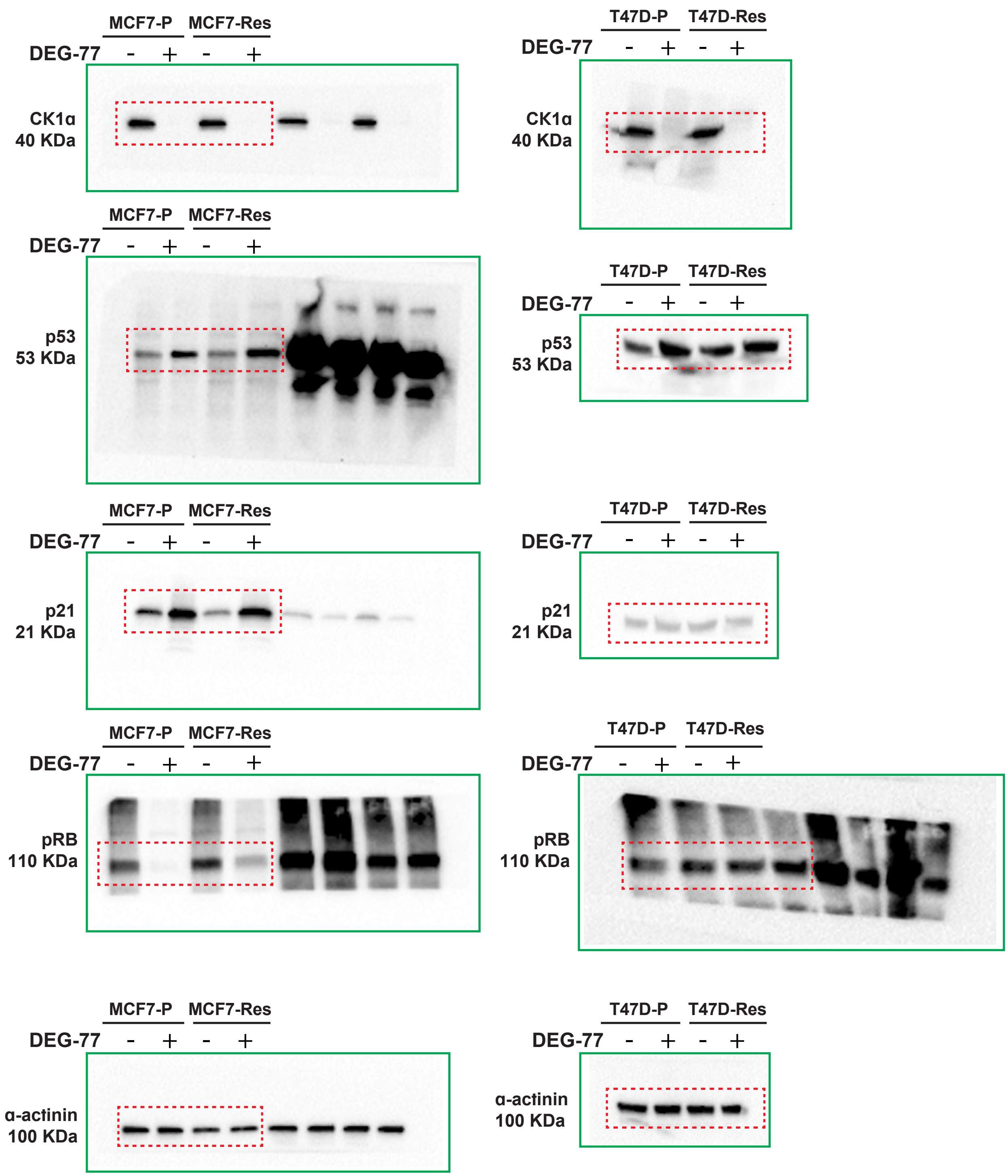

Supplementary Fig 13 Full unedited blot

Full unedited blot for Suppl Fig 13B

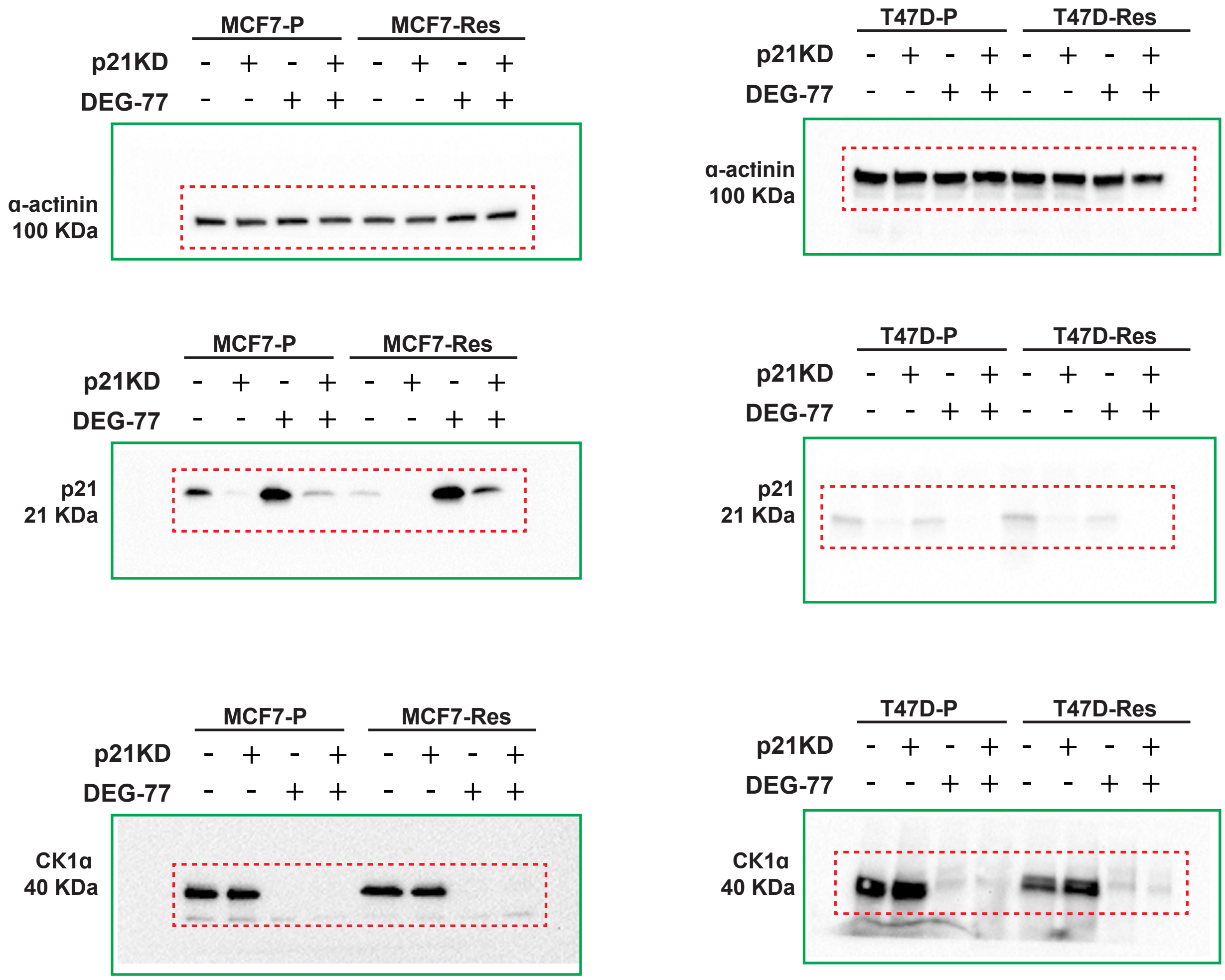

Full unedited blot for Suppl Fig 13H

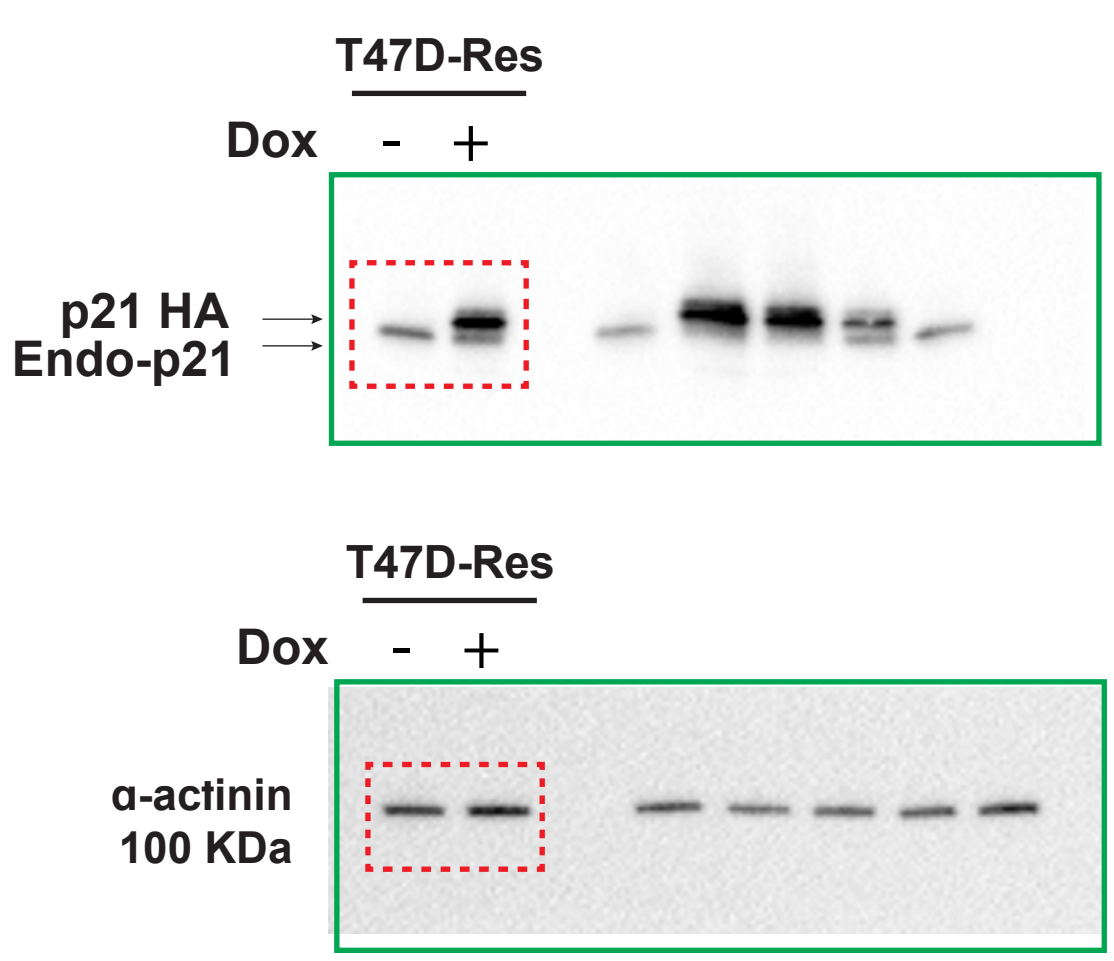

Supplementary Fig 14 Full unedited blot

Full unedited blot for Suppl Fig 14B

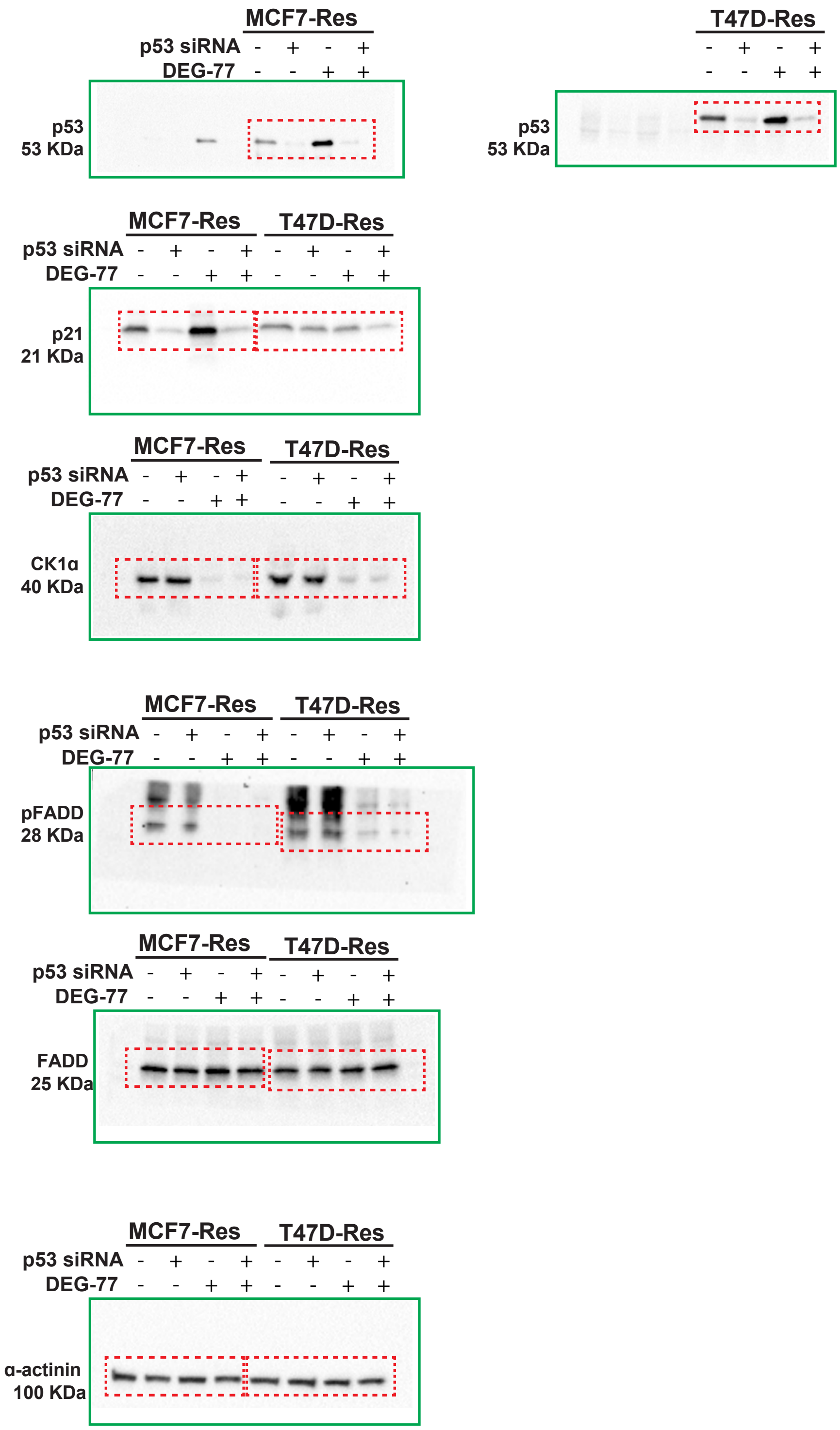

Supplementary Fig 15 Full unedited blot

Full unedited blot for Suppl Fig 15B

MCF7-Res

p21 siRNA	-	+	-	-	-	+	-	+
FADD siRNA	-	-	+	-	-	-	+	+
4/6i	-	-	-	+	+	+	+	+
DEG-77	-	-	-	-	+	+	+	+

p21
21 KDa

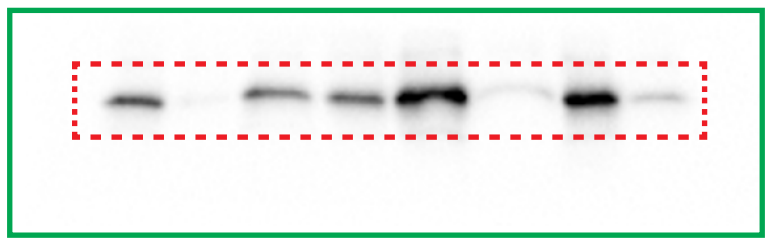

MCF7-Res

p21 siRNA	-	+	-	-	-	+	-	+
FADD siRNA	-	-	+	-	-	-	+	+
4/6i	-	-	-	+	+	+	+	+
DEG-77	-	-	-	-	+	+	+	+

FADD
25 KDa

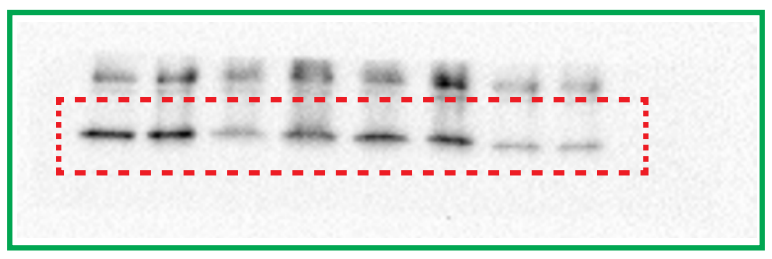

MCF7-Res

p21 siRNA	-	+	-	-	-	+	-	+
FADD siRNA	-	-	+	-	-	-	+	+
4/6i	-	-	-	+	+	+	+	+
DEG-77	-	-	-	-	+	+	+	+

CK1α
40 KDa

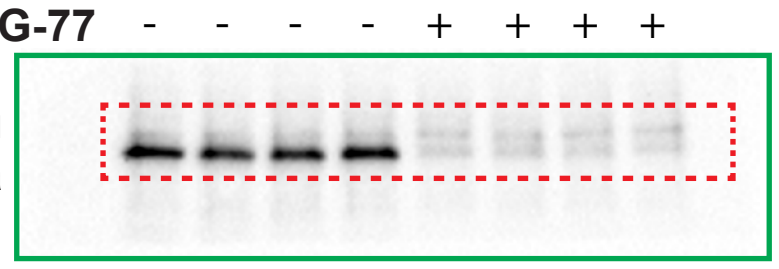

MCF7-Res

p21 siRNA	-	+	-	-	-	+	-	+
FADD siRNA	-	-	+	-	-	-	+	+
4/6i	-	-	-	+	+	+	+	+
DEG-77	-	-	-	-	+	+	+	+

p53
53 KDa

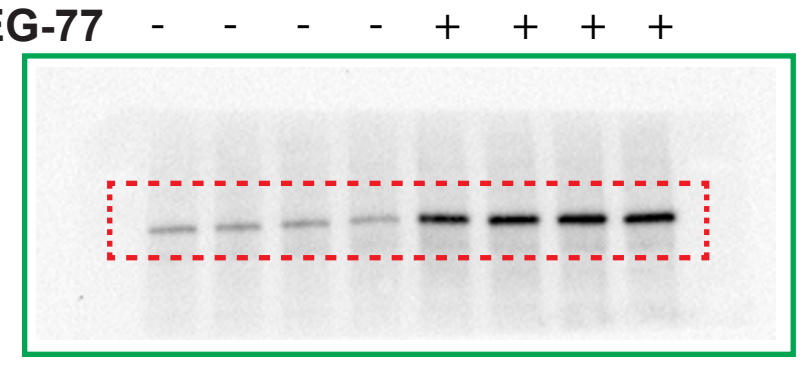

MCF7-Res

p21 siRNA	-	+	-	-	-	+	-	+
FADD siRNA	-	-	+	-	-	-	+	+
4/6i	-	-	-	+	+	+	+	+
DEG-77	-	-	-	-	+	+	+	+

pFADD
28 KDa

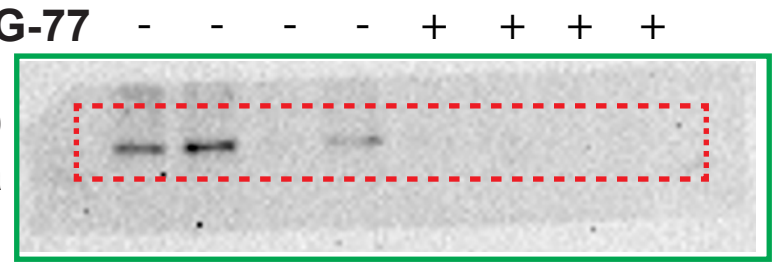

MCF7-Res

p21 siRNA	-	+	-	-	-	+	-	+
FADD siRNA	-	-	+	-	-	-	+	+
4/6i	-	-	-	+	+	+	+	+
DEG-77	-	-	-	-	+	+	+	+

α-actinin
100 KDa

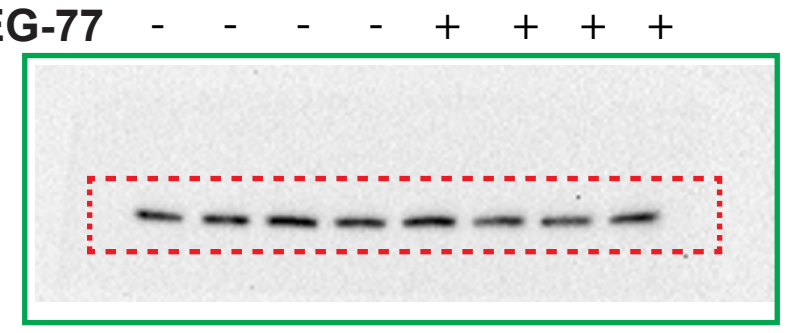

Supplementary Fig 16 Full unedited blot

Full unedited blot for Suppl Fig 13D

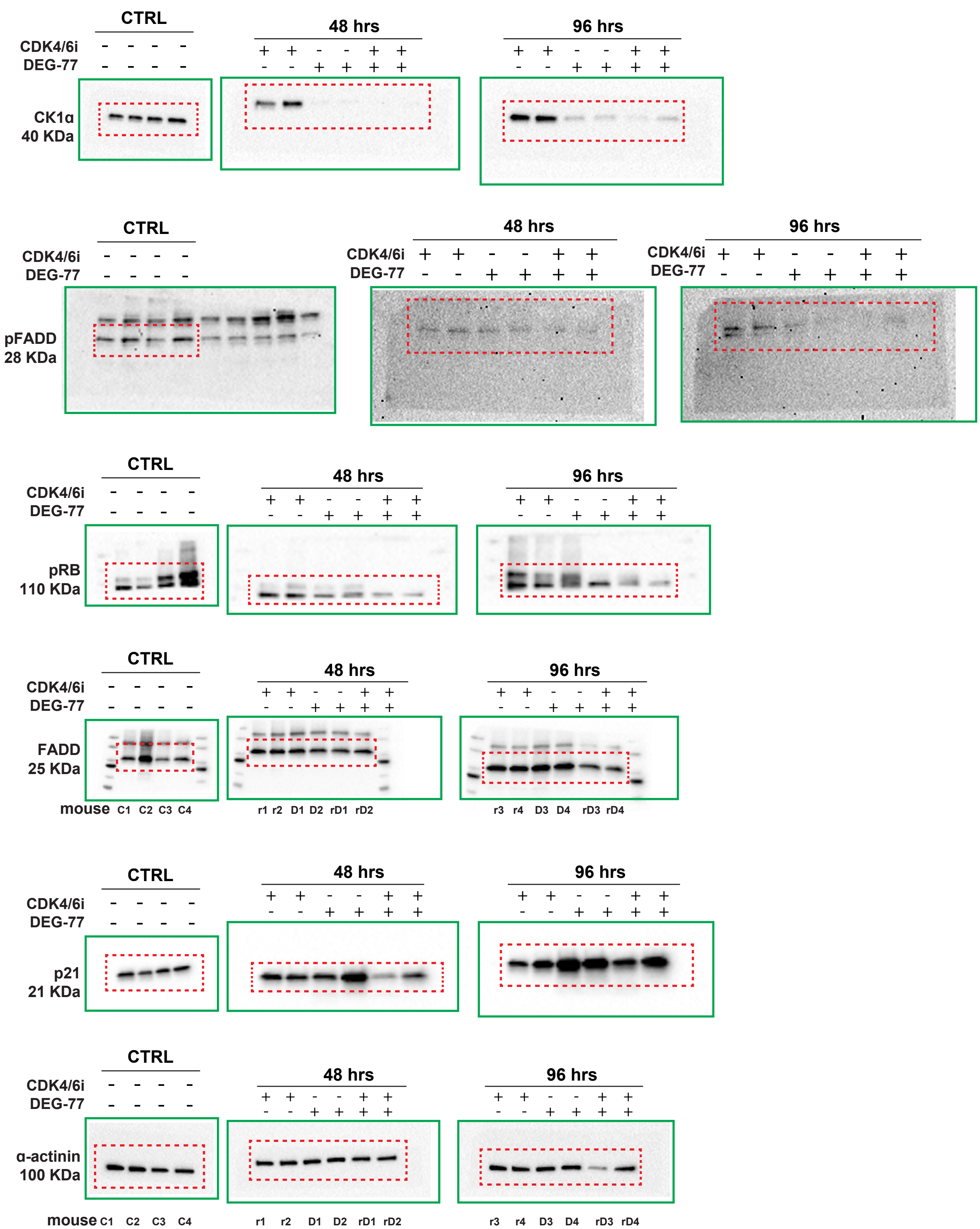

Supplement: Supplementary file 2 [file jci-136-199483-s126.pdf]
